# Bone marrow stromal cells enhance multiple myeloma cells proliferation through regulating LncRNA OVAAL/ENPP1 axis

**DOI:** 10.1515/biol-2025-1322

**Published:** 2026-05-14

**Authors:** Zhenjie Cai, Xiuli Chen, Heyong Zheng, Rongrong Zheng, Rong Zheng, Wuqiang Lin

**Affiliations:** Department of Hematology, The First Hospital of Putian City, 351100, Putian, China; The School of Clinical Medicine, Fujian Medical University, 350122, Fuzhou, China

**Keywords:** multiple myeloma, bone marrow stromal cells, OVAAL, LncRNA

## Abstract

Previous research has established the pivotal role of bone marrow stromal cells (BMSCs) in supporting multiple myeloma (MM) pathogenesis. However, the precise molecular mechanisms underlying this role remain elusive. To investigate this, we established a co-culture system of MM cells (U266) with BMSCs (HS-5), demonstrating that HS-5 significantly promoted U266 proliferation. Subsequent RNA sequencing identified HS-5-induced differentially expressed long non-coding RNAs (lncRNAs) and mRNAs. Pathway enrichment analysis, clinical sample validation, and *in vitro* functional assays were then employed to elucidate the mechanistic basis of HS-5-mediated MM cell proliferation. A total of 79 downregulated RNAs and 619 upregulated RNAs induced by HS-5 were identified. Pathway enrichment analysis of these mRNAs indicated that the pantothenate and coenzyme A biosynthesis pathway functions as a convergence hub enriched by both up-regulated and down-regulated mRNAs, with ENPP1 being enriched in this pathway. Further lncRNA data analysis and validation experiments established lncRNAs OVAAL’s targeting of ENPP1 and HS-5-mediated regulation of OVAAL. Additional *in vitro* experiments showed that OVAAL knockdown reduce the levels of LDH, GLU, and ATP and increase the levels of ROS in MM cells, which were reversed by co-culture with HS-5 cells. Knockdown of ENPP1 significantly suppresseed MM cell proliferation. This suppression could be rescued by co-culture with HS-5 stromal cells. This work indicates that BMSCs drive MM progression via OVAAL upregulation, which activates the ENPP1-mediated cells proliferation and metabolic reprogramming.

## 1 Introduction

Multiple myeloma (MM), a malignant plasma cell disorder characterized by marked genomic heterogeneity [1], exhibits highly variable clinical outcomes. It represents one of the most prevalent hematologic malignancies, accounting for over 10 % of blood cancers and approximately 1 % of all cancers [2]. The American Cancer Society estimates MM as the second most common hematologic malignancy in the US, projecting 35,780 new cases and 12,540 deaths for 2024 [3]. Despite improved response and survival outcomes driven by novel therapeutics and combinations, most patients face indefinite treatment [4], 5]. Nevertheless, therapeutic resistance and relapse remain pervasive, inevitably leading to refractory disease. Thus, elucidating MM pathogenesis to identify novel molecular targets and optimize treatment regimens is crucial for clinical advancement.

The aggregation of MM cells within the bone marrow substantiates the supportive role of the tumor microenvironment in MM clonal growth. The bone marrow microenvironment comprises both cellular and non-cellular components. Cellular constituents include bone marrow stromal cells (BMSCs), vascular endothelial cells, osteoblasts, osteoclasts, and various immune cells. ‌BMSCs, constituting 60–70 % of the cellular compartment within this microenvironment, are a critical element.‌ They regulate hematopoiesis by modulating the balance between self-renewal and differentiation of hematopoietic stem and progenitor cells through mechanisms such as cell-to-cell contact and cytokine signaling [6], 7]. Compared to exosomes from healthy BMSCs, those derived from BMSCs in MM demonstrate increased cargo of cytokines, oncoproteins, and adhesion molecules [8]. Notably, BMSCs promote MM cell homing, survival, proliferation, and drug resistance via paracrine secretion of factors including interleukin-6, vascular endothelial growth factor, fibroblast growth factor, and tumor necrosis factor-α [9], 10]. These observations underscore the pivotal role of BMSCs in supporting MM pathogenesis, though the exact molecular mechanisms remain incompletely defined.

To address this, we co-cultured MM cells U266 with BMSCs HS-5 and observed that HS-5 promoted U266 cell proliferation. Subsequently, RNA sequencing identified HS-5-induced differentially expressed long non-coding RNAs (lncRNAs) and messenger RNAs (mRNAs). Further pathway enrichment analysis, clinical sample validation, and *in vitro* validation were conducted to reveal the potential molecular mechanisms underlying HS-5-mediated promotion of MM cell proliferation.

## 2 Materials and methods

### 2.1 Patients

MM patients admitted to the First hospital of Putian City from January 2024 to June 2024 were enrolled and confirmed according to the criteria of International Myeloma Working Group. Patients meeting any of the following criteria were excluded [1]: Diagnosis of solitary plasmacytoma [2]; Presence of other immunoglobulin proliferative disorders [3]; Concurrent severe cardiovascular or cerebrovascular diseases. Bone marrow samples were collected from peripheral sites, and mononuclear cells were isolated for subsequent analysis. Normal controls consisted of age-matched bone marrow donations from volunteers with no history of hematologic malignancies or solid tumors, and without severe cardiac, hepatic, or renal dysfunction.

**Informed consent:** Informed consent has been obtained from all individuals included in this study.

**Ethical approval:** The research related to human use has been complied with all the relevant national regulations, institutional policies and in accordance with the tenets of the Helsinki Declaration, and has been approved by the Ethics Committee of the First Hospital of Putian City (No. 2023-076).

### 2.2 Cell culture

The human multiple myeloma cell line U266 and MM.1S were purchased from Shanghai Zhong Qiao Xin Zhou Biotechnology Co., Ltd (ZQ0619) and cultured in RPMI 1640 medium (Procell, PM150110) supplemented with 15 % fetal bovine serum (FBS, Excell Bio, FSP500), and 1 % penicillin/streptomycin. The human bone marrow stromal cell line HS-5 was purchased from Procell (CL-0798) and cultured in DMEM medium (Procell, PM150210) supplemented with 10 % FBS (Excell Bio, FSP500), and 1 % penicillin/streptomycin. All the cells were maintained in a humidified atmosphere with 5 % CO_2_ at 37 °C.

### 2.3 Co-culture of U266 cells with HS-5 cells

HS-5 cells at logarithmic phase were seeded into 6-wells at 8 × 10^4^ cells/well or 1.6 × 10^5^ cells/well. Upon confluence, each group of U266 cells (8 × 10^4^ cells/well) were added. Thereafter, the cells were cultured for 24, 48, and 72 h in a humidified atmosphere with 5 % CO_2_ at 37 °C.

### 2.4 Cell counting kit-8

Following the required cell culture period, cells were collected, resuspended in 500 μL of culture medium containing cell counting kit-8 (CCK-8), and transferred into a 12-well plate according to experimental groups. After incubation at 37 °C for 2 h, 100 μL aliquots of supernatant were collected and transferred to a 96-well plate. Optical density (OD) values were measured at 450 nm using a microplate reader. Cell viability was calculated using the following formula: Cell viability = (OD_(experimental)_−OD_(blank)_)/(OD_(control)_−OD_(blank)_).

### 2.5 RNA-sequencing library preparation and ‌high-throughput sequencing

Total RNA (1 μg per sample) was treated with RQ1 DNase (M6101, Promega) for genomic DNA removal. Directional RNA-seq libraries were constructed using the VAHTS^®^ Universal V8 RNA-seq Library Prep Kit for Illumina^®^ (NR605, Vazyme) according to manufacturer specifications. Polyadenylated mRNAs were enriched with VAHTS mRNA Capture Beads (N401, Vazyme), followed by fragmentation and double-stranded cDNA synthesis. After end repair and 3′ adenylation, adapters were ligated using VAHTS RNA Multiplex Oligos Set 1 for Illumina (N323, Vazyme). Ligated products underwent PCR amplification, purification, and quantification, with final libraries stored at −80 °C. Strand specificity was maintained through dUTP incorporation in the second cDNA strand, which blocks amplification during PCR. Library quality control was performed before loading onto the Illumina NovaSeq XPlus platform. Paired-end sequencing (150 nucleotide reads) was conducted following standard Illumina protocols.

### 2.6 Data analysis

For mRNA analysis, raw sequencing data underwent initial quality control using Trimmomatic (v0.36), where low-quality reads were removed and adapter-contaminated sequences were trimmed. The resulting clean reads were processed with custom scripts to remove duplication artifacts from library preparation and sequencing. Following sub-cluster generation, multiple sequence alignment produced consensus sequences for each sub-cluster. These deduplicated reads were then aligned to the human reference genome (GRCh37/hg19) using STAR (v2.5.3a) with default parameters. Gene-level quantification was performed by featureCounts (Subread-1.5.1) counting exon-mapped reads, followed by fragments per kilobase million calculation. Differential expression analysis was conducted with edgeR (v3.12.1), applying significance thresholds of *p* < 0.05 and absolute fold-change >2. Finally, KOBAS (v2.1.1) performed KEGG pathway enrichment analysis on differentially expressed mRNAs using a significance cutoff of *p* < 0.05.

For lncRNA analysis, novel lncRNAs were predicted. Briefly, mapped reads were assembled into transcripts using StringTie (v1.3.2). These novel transcripts were then filtered, and their coding potential was assessed through a comprehensive four-method analysis: CPC2 (beta), CPAT (v1.2.4), CNCI (v2), and Pfam (v27.0). Transcripts consistently predicted as non-coding by all four tools/platforms were designated as novel lncRNAs. Expression quantification for both novel lncRNAs and genome-annotated known lncRNAs was performed using featureCounts (Subread-1.5.1/Bioconductor), followed by RPKM calculation. Differentially expressed lncRNAs between groups were identified using edgeR (v3.12.1), applying significance thresholds of *p*-value < 0.05 and absolute fold-change > 2. Co-expression patterns between differentially expressed mRNAs and lncRNAs were analyzed using weighted correlation network analysis (WGCNA v1.51), applying thresholds of weight value > 0.6 and signed scale-free topology fit (*R* ^2^) > 0.6.

### 2.7 Real-time quantitative polymerase chain reaction (RT-qPCR)

Total RNA was extracted using TRIzol, quantified (A260/A280 > 1.8), and reverse-transcribed to cDNA (HiScript^®^ II Q RT SuperMix, VAZYME, R223-01). qPCR was performed in triplicate using SYBR Green Master Mix (VAZYME, Q311-02) with gene-specific primers (Table 1). Cycling conditions: 95 °C/10 min; 40 cycles of 95 °C/15 s, 60 °C/60 s, 95 °C/15 s; 60 °C/60 s and 95 °C/15 s. Melt curve analysis confirmed specificity. Relative quantification used the 2^−ΔΔCt^ method with GAPDH normalization.

**Table 1:** Gene-specific primers for RT-qPCR.

Gene	Primer	Sequence (5′-3′)	PCR products (bp)
Homo GAPDH	Forward	ATG​GGG​AAG​GTG​AAG​GTC​GGA​GT	125
	Reverse	TAG​TTG​AGG​TCA​ATG​AAG​GGG​TC	
Homo UPB1	Forward	CTA​TGC​CCT​TTG​CCT​TCT​GTA	157
	Reverse	CCA​TGC​TCG​CTG​TCT​CGT​TCC	
Homo ENPP1	Forward	AGG​GTT​GAT​GGT​ATG​GTT​GGT	289
	Reverse	GGT​AAG​GTT​TGA​AGT​GCT​GGT	
Homo LINC01235	Forward	AGG​AGA​ACA​GAG​AGC​AAA​G	101
	Reverse	GAA​GAT​GAT​GAT​GGA​GTA​G	
Homo OVAAL	Forward	GGA​ACT​GAG​GCA​GCT​GTA​TGG	171
	Reverse	TTG​ATG​GGT​CTG​AAA​GGA​AAC	
Homo FOXD3-AS1	Forward	CAA​CAA​AGG​GAC​GAG​AGA​CGC	255
	Reverse	TGG​GGG​AGG​GGG​AAA​AAG​ACA	
Homo ENPP3	Forward	GCC​ACA​GAA​AGA​AAT​GGA​GTA	223
	Reverse	GGT​GAG​GGA​TGA​TAA​AGG​GTA	

### 2.8 U266 and MM.1S cells transfection

MM.1S or U266 cells in the logarithmic growth phase were harvested and adjusted to a concentration of 2.5 × 10^5^ cells/mL in culture medium. Subsequently, 2 mL of the cell suspension were seeded per well into a 6-well plate and cultured overnight at 37 °C in a humidified incubator with 5 % CO_2_. Two hours prior to transfection, the medium was replaced with serum-free medium. For transfection complex preparation, 10 μL of OVAAL small interfering (si) RNA (87, 704, 547, and 1,072) were diluted to 100 μL with serum-free Opti-MEM in one tube, while 5 μL of Lipofectamine™ 2000 were separately diluted to 100 μL with Opti-MEM in another tube. The diluted Lipofectamine™ 2000 was then combined with the diluted small fragments, mixed gently, and the resulting transfection cocktail was added dropwise to the respective wells. Cells were maintained in the 37 °C, 5 % CO_2_ incubator, and after 4–6 h of incubation, the transfection mixture was aspirated and replaced with normal culture medium. Transfection efficiency was assessed 24 h following the initiation of transfection.

### 2.9 Western blot

Total protein was extracted from each experimental group using RIPA lysis buffer (Meilunbio, MA0151) and quantified via BCA assay (GBCBIO, G3422). Proteins (40 μg per group) were electrophoresed and transferred to PVDF membranes (Millipore, USA). After blocking with 5 % skim milk (2 h), membranes were incubated overnight at 4 °C with primary antibodies targeting ENPP1 (ABCLONAL, A17876) and GAPDH (Proteintech, 60004-1-Ig; loading control). Subsequently, membranes were incubated with HRP-conjugated secondary antibody and protein signals were detected using chemiluminescence reagents (Affinity, KF8003).

### 2.10 Flow cytometry for reactive oxygen species (ROS) assay

Following treatment, cells were collected by centrifugation at 1,500 rpm for 5 min, followed by resuspending in phosphate-buffered saline (PBS). The cells were then centrifuged again at 1,500 rpm for 5 min, and were resuspended in 1 mL of serum-free medium containing dihydroethidium (Beyotime, S0063) diluted 1:1000 and incubated at 37 °C for 20 min. Subsequently, the cells were washed three times with serum-free medium. Finally, the cell pellet was resuspended in 500 μL of PBS and subjected to a flow cytometry (Beckmancoulter, cytoFLEX).

### 2.11 Biochemical assay

The levels of lactate dehydrogenase (LDH, Nanjing Jiancheng Bioengineering Institute, A020-2-1), adenosine triphosphate (ATP, Solarbio, BC0305), and glucose (GLU, Nanjing Jiancheng Bioengineering Institute, A154-1-1) were assayed using corresponding detection kit according to manufacturer’s instruction.

### 2.12 Statistical analysis

All statistical analyses were performed using GraphPad Prism 9.0. Data represents the mean ± standard deviation (SD) of three replicates (*n* = 3). Comparisons between two groups were performed using Student’s *t*-test, while comparisons among more than two groups were performed using the one-way analysis of variance followed by Tukey’s post-hoc test. Statistical significance was defined as *p*-value < 0.05.

## 3 Results

### 3.1 BMSCs promotes MM cells proliferation through regulation pantothenate and CoA biosynthesis

**Figure 1:**
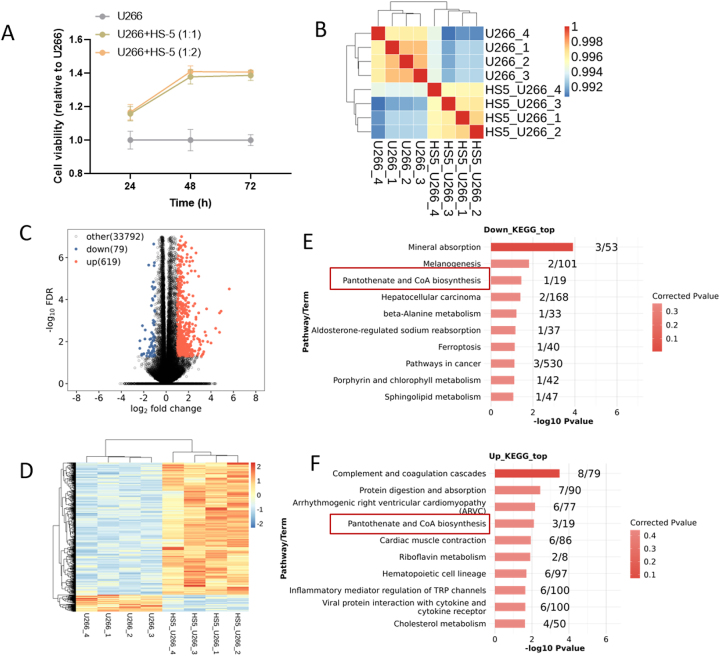
RNA data analysis of RNA sequencing. (A) The cell proliferation detection using CCK-8. (B) Sample correlation analysis. (C) Volcano plot of differentially expressed RNAs between U266 cells and co-cultured system. (D) heatmap clustering of differentially expressed RNAs between U266 cells and co-cultured system. Pathway enrichment analysis of downregulated (E) and upregulated (F) differentially expressed mRNAs.

The cell proliferation detection using CCK-8 demonstrated the pro-proliferative effect of BMSCs on MM cells (Figure 1A, standardized to the value of the U266 group at the 24-h time point). ‌Since the pro-proliferative effect at a U266 and HS-5 concentration ratio of 1:1 was comparable to that at 1:2, a ratio of 1:1 was employed for subsequent experiments. To further reveal the molecular mechanisms by which BMSCs act on MM cells, RNA sequencing was performed. Data analysis revealed that intra-group sample correlation was significantly greater than inter-group correlation, with correlation coefficients exceeding 0.995, indicating sound experimental design and sampling (Figure 1B). Differential RNA analysis identified 79 downregulated RNAs (blue points) and 619 upregulated RNAs (red points) induced by HS-5 (Figure 1C, Supplementary 1).‌ Heatmap cluster analysis demonstrated that the differentially expressed RNA effectively separated the two sample groups (Figure 1D). Moreover, pathway enrichment analysis of the differentially expressed mRNA indicated that the pantothenate and CoA biosynthesis pathway represented a convergence point enriched by both upregulated and downregulated mRNAs (Figure 1E and F). Key enriched genes included downregulated DPYS and upregulated UPB1, ENPP3, and ENPP1 (Supplementary 2 and 3), and ‌upregulated genes tentatively identified as key regulatory genes were selected for subsequent analysis.

### 3.2 LncRNA OVAAL is a major element of BMSCs-mediated regulation in MM

**Figure 2:**
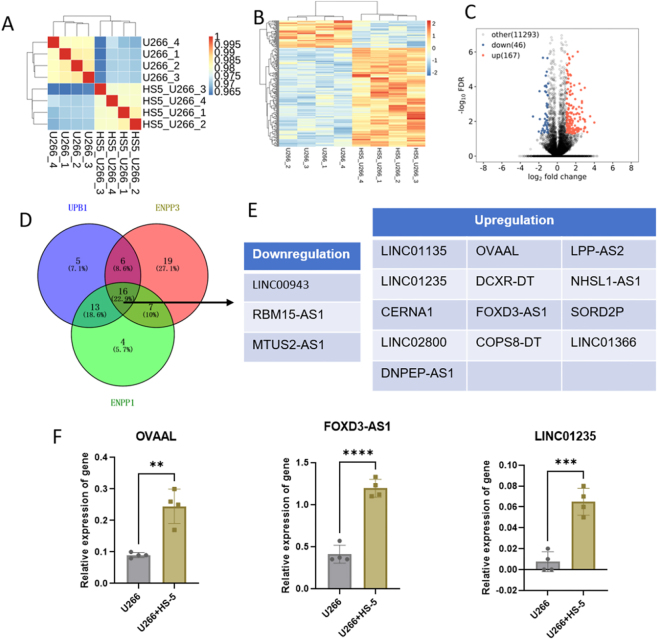
LncRNA data analysis of RNA sequencing. (A) Sample correlation analysis. (B) Heatmap clustering of differentially expressed lncRNAs between U266 cells and co-cultured system. (C) Volcano plot of differentially expressed lncRNAs between U266 cells and co-cultured system. (D) Venn diagram of differentially expressed lncRNAs targeting UPB1, ENPP3, and ENPP1. (E) The specific differentially expressed lncRNAs concurrently targeting UPB1, ENPP3, and ENPP1. (F) The relative expression of OVAAL, FOXD3-AS1, and LINCO1235 in U266 cells and co-cultured system detected using RNA sequencing. **p < 0.01; ***p < 0.001; ****p < 0.0001.

**Figure 3:**
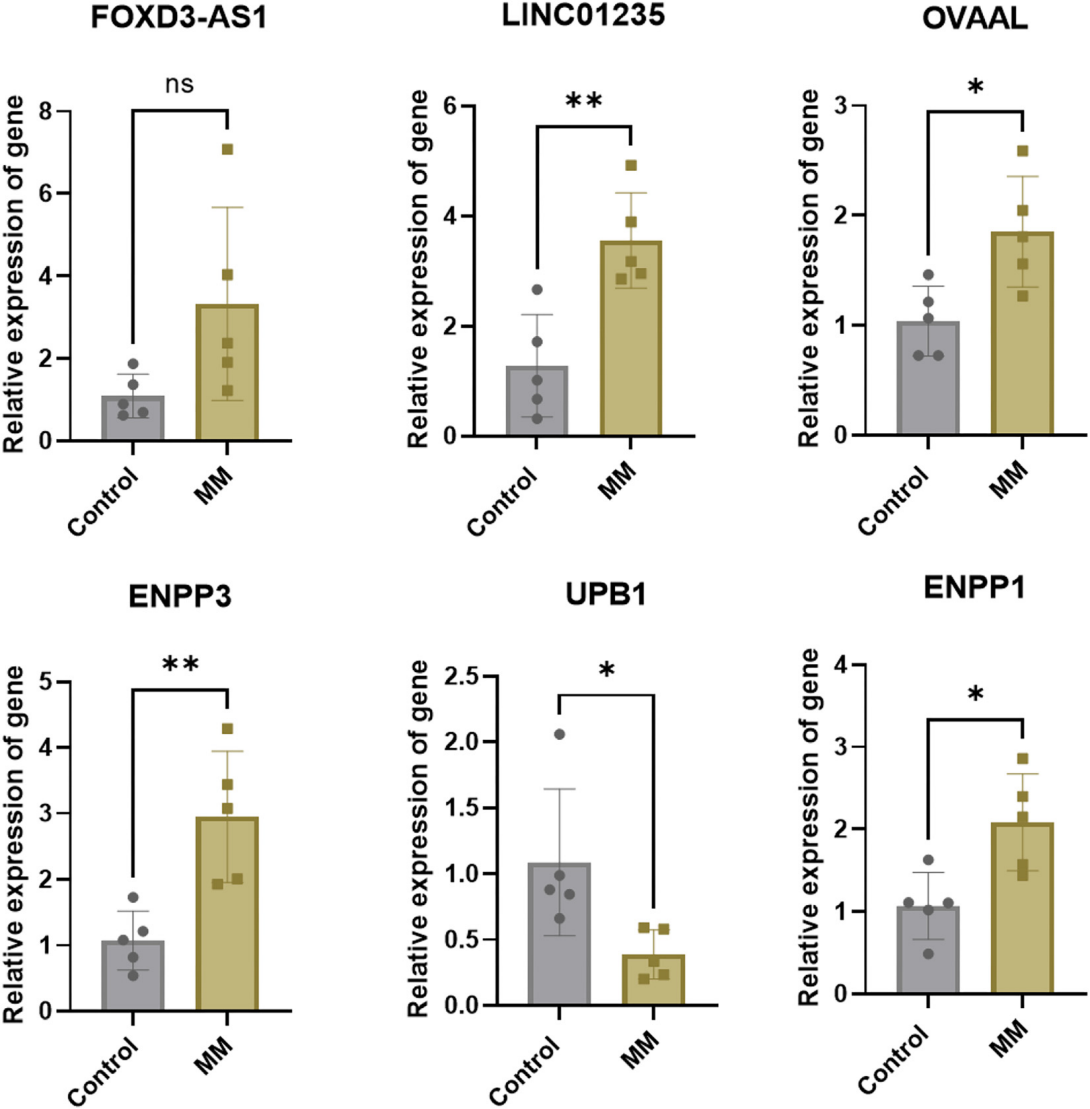
Clinical bone marrow mononuclear cells samples were employed to detect the relative expression of OVAAL, FOXD3-AS1, LINCO1235, UPB1, ENPP3, and ENPP1 in MM patients and healthy controls. *p < 0.05; **p < 0.01; ns, no significant.

**Figure 4:**
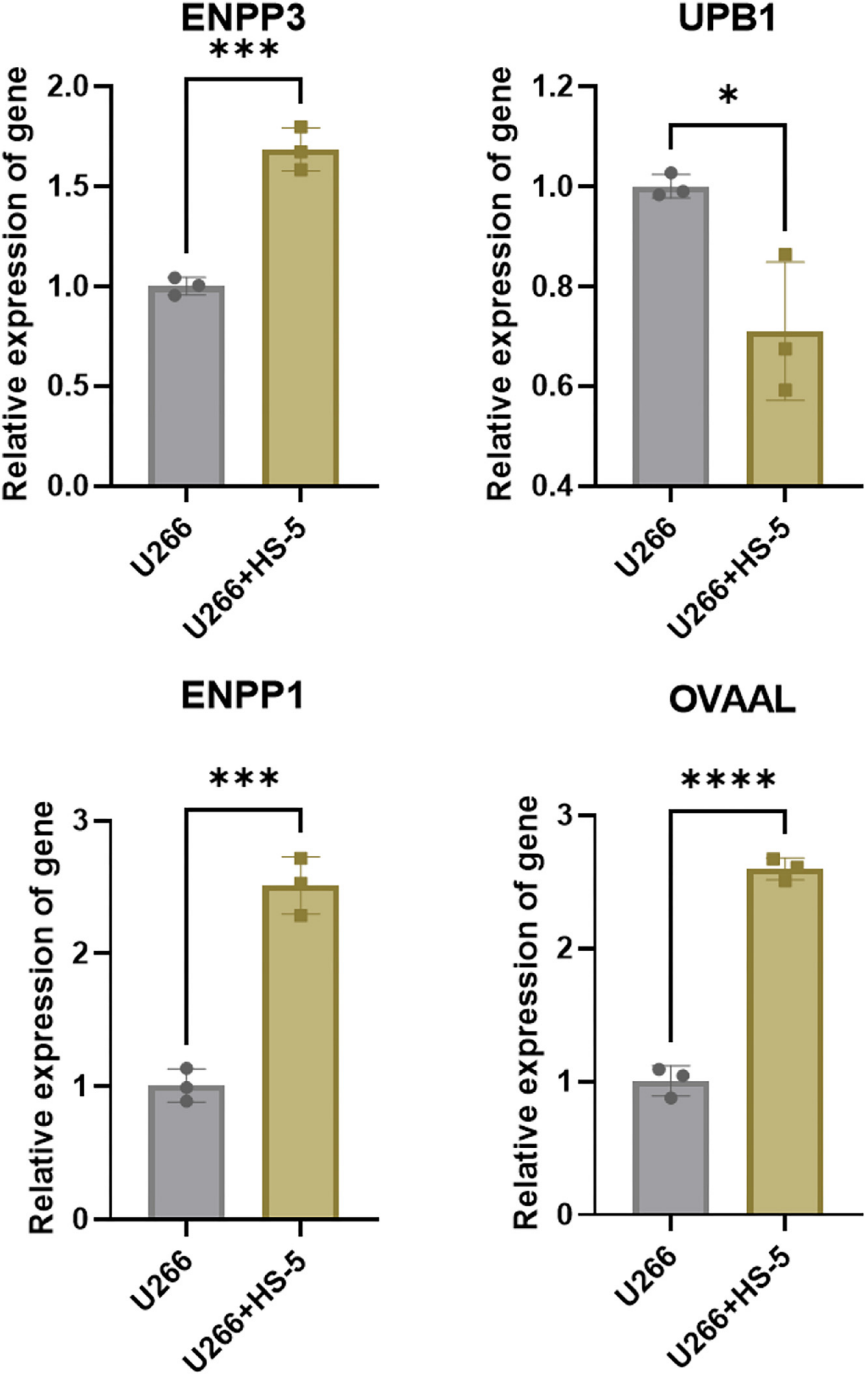
The relative expression of OVAAL, UPB1, ENPP3, and ENPP1 were validated in U266 cells and co-cultured system. *p < 0.05; ***p < 0.001; ****p < 0.0001.

To identify upstream regulatory lncRNAs targeting mRNAs, we analyzed lncRNA sequencing data. Similarly, correlation clustering (Figure 2A) and differential lncRNA heatmap clustering (Figure 2B) revealed distinctions between the two groups. HS-5 cell induction resulted in 46 upregulated and 167 downregulated lncRNAs (Figure 2C). Co-expression analysis demonstrated that 40 differentially expressed lncRNAs targeted UPB1, 38 targeted ENPP3, and 40 targeted ENPP1 (Figure 2D). Intersection analysis identified 16 differentially expressed lncRNAs concurrently targeting UPB1, ENPP3, and ENPP1, comprising 3 downregulated and 13 upregulated lncRNAs (Figure 2E). Based on their documented roles in tumorigenesis, we prioritized the well-studied lncRNAs OVAAL, FOXD3-AS1, and LINC01235 (Figure 2F). Clinical bone marrow mononuclear cells sample analysis revealed that while lncRNA FOXD3-AS1 expression showed no statistically significant difference in MM patients compared to control volunteers, OVAAL, LINC01235, ENPP3, and ENPP1 were significantly upregulated, and UPB1 was significantly downregulated (Figure 3). Due to the long sequence length of LINC01235, which resulted in low detection sensitivity and challenges in primer design, we ultimately selected OVAAL as the focus for further investigation. Further validation through cell co-culture demonstrated the ability of BMSCs to induce elevated expression of OVAAL, ENPP3, and ENPP1, while decreased expression of UPB1 (Figure 4).

### 3.3 BMSCs regulates MM cell proliferation and metabolic reprogramming by regulating LncRNA OVAAL/ENPP1 axis

**Figure 5:**
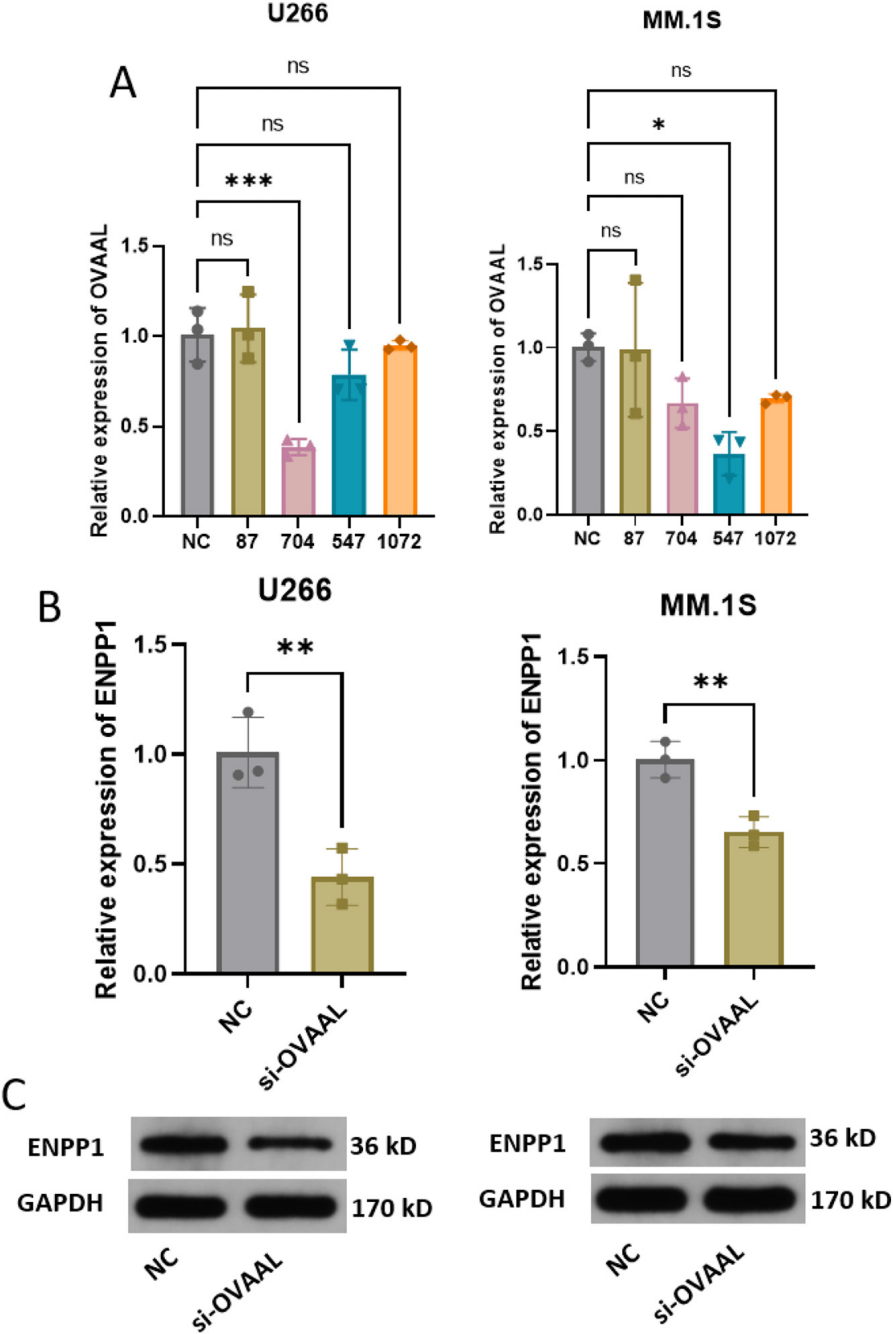
The interference of OVAAL expression results in ENPP1 expression suppression. (A) The relative expression of OVAAL detection using RT-qPCR. (B) The relative expression of ENPP1 detection using RT-qPCR. (C) The expression of ENPP1 protein detection using western blot. *p < 0.05; **p < 0.01; ***p < 0.001; ns, no significant.

**Figure 6:**
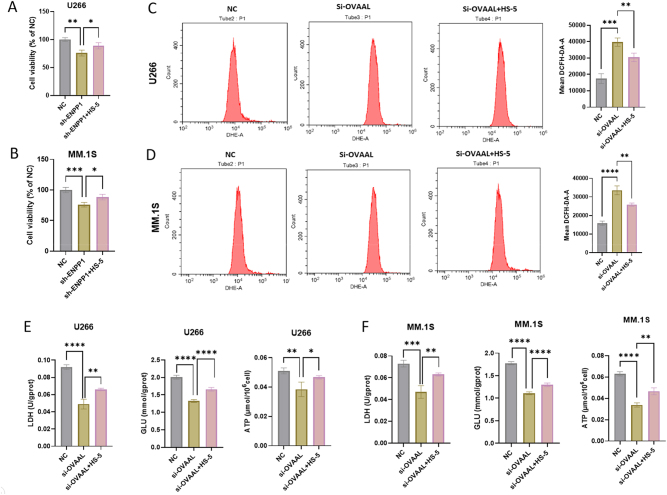
The interference of OVAAL expression results in a metabolic reprogramming MM cell. The cell proliferation of U266 (A) and MM.1S (B) detection using CCK-8. The level of ROS in U266 (C) and MM.1S (D) cells detection using flow cytometry. The levels of LDH, GLU, and ATP in U266 (E) and MM.1S (F) cells. *p < 0.05; **p < 0.01; ***p < 0.001; ****p < 0.0001. ROS, reactive oxygen species; LDH, lactate dehydrogenase; ATP, adenosine triphosphate; GLU, glucose.

To validate OVAAL’s function, we suppressed its expression in MM cells using specific siRNAs (si-704 in U266 cells and si-547 in MM.1S cells) (Figure 5A). Functional assays demonstrated that OVAAL inhibition attenuated ENPP1 expression in both U266 and MM.1S cells (Figure 5B and C). ENPP1 downregulation inhibited proliferation of both U266 and MM.1S cells, which was rescued by co-culture with HS-5 cells (Figure 6A and B). Furthermore, OVAAL knockdown increased the levels of ROS (Figure 6C and D), and reduced the levels of LDH, GLU, and ATP levels (Figure 6E and F) in MM cells, which were reversed by co-culture with HS-5 cells.

## 4 Discussion

As highlighted in the provided recent studies, BMSCs are central to the MM microenvironment, driving pathology through diverse mechanisms. Notably, research demonstrated that direct MM-BMSC contact via the CD40-CD40L axis is a critical signaling hub. This interaction inhibits osteogenesis by downregulating SENP1 in BMSCs to disrupt primary cilia formation [11]. Intriguingly, BMSCs exhibit a dual role in regulating ferroptosis in MM cells: they can protect MM cells by upregulating SENP3 to stabilize the anti-ferroptotic protein GPX4 [12], yet under other conditions, they can sensitize MM cells to ferroptosis by enhancing iron accumulation and lanosterol biosynthesis via the same CD40/CD40L pathway [13]. These findings underscore the complex, context-dependent regulation of non-apoptotic cell death by the niche. In current study, the findings suggested the pro-proliferative effect of BMSCs on MM cell. Mechanically, RNA sequencing implicated the pantothenate and CoA biosynthesis pathway as playing a pivotal role in this process. Coenzyme A (CoA) is indispensable for mitochondrial and peroxisomal catabolism of nutrients, generating reducing equivalents, energy, and biosynthetic precursors [14], [15], [16], [17]. Beyond its catabolic functions, CoA directly facilitates essential anabolic processes–including lipid, glycan, and haem biosynthesis [18]. Its biosynthesis requires ATP, cysteine, and pantothenate (more colloquially as vitamin B_5_), and pantothenate is exclusively dedicated to CoA biosynthesis [19]. Due to the fundamental role of CoA in metabolism, dysregulation of its synthesis likely contributes to multiple common diseases, including cancer where altered metabolite processing drives initiation and progression [18], 20]. Emerging evidence specifically linked CoA synthesis perturbations to tumorigenesis [21] and therapeutic resistance [22], [23], [24], [25]. As the obligate precursor of CoA, pantothenate critically enables glycolytic carbon entry into the Krebs cycle and facilitates the α-ketoglutarate-to-succinate conversion within this cycle. GLU and glutamine serve as primary carbon sources feeding this metabolic flux [26], 27]. Notably, Kreuzaler et al., through correlative mass spectrometry imaging, demonstrated pantothenate accumulation in MYC-high regions of human and murine mammary tumors. Here, its conversion to CoA drives Krebs cycle activity, fueling breast cancer oncogenesis via metabolic reprogramming [28]. In this work, the potential regulatory effect of BMSCs on the pantothenate and CoA biosynthesis pathway in MM cells was only identified through transcriptomic sequencing. Targeting this pathway might represent a promising strategy to disrupt BMSC-supported MM growth by inhibiting pro-tumor metabolic crosstalk. However, further *in vitro* and *in vivo* experiments are required for validation.

We further observed that ENPP1 is a key differentially expressed mRNA enriched in the pantothenate and CoA biosynthesis pathway. ENPP1 has been found to be associated with poor prognosis in various cancers, including ovarian carcinoma [29], bladder cancer [30], and breast cancer [31]. Previous studies on ENPP1 in tumors have primarily focused on its immunomodulatory roles. Emerging evidence indicates that ENPP1 hydrolyzes the immunostimulatory metabolite 2′,3′-cGAMP, while concurrently promoting adenosine production within the tumor microenvironment. This enzymatic activity suppresses cGAS-STING-dependent immune activation, ultimately establishing an immunosuppressive niche that drives tumor immune evasion and metastatic progression. [32], 33]. This work revealed a novel implication of ENPP1 in tumor metabolic regulation. Subsequent lncRNA analysis and validation experiments established OVAAL’s targeting of ENPP1 and HS-5-mediated regulation of OVAAL. ENPP1 downregulation inhibited proliferation of MM cells. The role of OVAAL in tumor metabolism has been experimentally validated. OVAAL stabilized pyruvate carboxylase, accelerating oxaloacetate-aspartate flux to enhance pyrimidine biosynthesis, thereby promoting gastric cancer cell proliferation and 5-fluorouracil resistance [34]. In addition, we found that OVAAL knockdown reduced the levels of LDH, GLU, and ATP and increased the levels of ROS in MM cells, which are associated with CoA metabolism [18], 35], 36]. These effects were reversed by co-culture with HS-5 cells. Collectively, this work indicated that BMSCs might enhance MM cell proliferation and metabolic reprogramming by up-regulating OVAAL/ENPP1 axis.

## 5 Conclusions

In this work, we co-cultured MM cell line U266 with BMSCs HS-5 and observed that HS-5 promoted U266 cell proliferation. Subsequent RNA sequencing identified HS-5-induced differentially expressed long non-coding RNAs (lncRNAs) and messenger RNAs (mRNAs). Pathway enrichment analysis revealed that the pantothenate and CoA biosynthesis pathway served as an intersection enriched by both up-regulated and down-regulated mRNAs. We further identified three up-regulated mRNAs (UPB1, ENPP3, and ENPP1), that enriched in pantothenate and CoA biosynthesis pathway, and their common upstream lncRNA. Additional validation using *in vitro* experiments demonstrated that BMSCs may enhance MM cell proliferation by up-regulating OVAAL to modulate the ENPP1. While our *in vitro* and clinical validation demonstrate BMSC-driven MM proliferation via the OVAAL-ENPP1-pantothenate axis, further ‌*in vivo*‌ and ‌large-cohort prospective clinical studies‌ are warranted to ‌strengthen and supplement‌ these conclusions.

## Supplementary Material

Supplementary Material

Supplementary Material

Supplementary Material
