# Supplementary material for: Bone marrow stromal cells enhance multiple myeloma cells proliferation through regulating LncRNA OVAAL/ENPP1 axis
Source: Open Life Sci. 2026 May 14;21(1):20251322. doi: 10.1515/biol-2025-1322 (PMC13170762; doi:10.1515/biol-2025-1322)
Supplement: Supplementary file 1 — Supplementary Material [file j_biol-2025-1322_suppl_001.docx]

| Supplementary 1 Differentially expressed RNAs between U266 and co-cultured system. | | | | |  |  |  |  |  |
| --- | --- | --- | --- | --- | --- | --- | --- | --- | --- |
| Gene | baseMean | log2FoldChange | lfcSE | stat | pvalue | padj | Symbol | Chr | GeneType |
| ENSG00000287709.3 | 13.271453 | 1.25248897 | 0.45525436 | 2.75118502 | 0.00593801 | 0.02260427 | ENSG00000287709 | chr5 | lncRNA |
| ENSG00000073711.11 | 21.7440471 | 1.05750919 | 0.38499073 | 2.74684328 | 0.00601719 | 0.022848 | PPP2R3A | chr3 | protein_coding |
| ENSG00000236719.5 | 10.9140664 | 1.34790535 | 0.47693907 | 2.82615839 | 0.004711 | 0.01863567 | OVAAL | chr1 | lncRNA |
| ENSG00000198535.5 | 47.8291614 | 1.29835094 | 0.23123956 | 5.61474414 | 1.97E-08 | 3.40E-07 | C2CD4A | chr15 | protein_coding |
| ENSG00000230387.5 | 5.04405484 | 2.80032942 | 0.88318775 | 3.17070682 | 0.00152069 | 0.00715346 | ENSG00000230387 | chr20 | lncRNA |
| ENSG00000197712.12 | 17.4646828 | 1.18253411 | 0.42105174 | 2.80852445 | 0.00497691 | 0.01951208 | FAM114A1 | chr4 | protein_coding |
| ENSG00000277301.1 | 6.68555204 | 1.74689676 | 0.69478423 | 2.5143011 | 0.01192686 | 0.04034606 | ENSG00000277301 | chr20 | lncRNA |
| ENSG00000171236.10 | 12.04627 | 1.52637164 | 0.50898 | 2.99888332 | 0.00270971 | 0.01165358 | LRG1 | chr19 | protein_coding |
| ENSG00000163545.11 | 41.4155277 | 1.17530785 | 0.25633677 | 4.58501469 | 4.54E-06 | 4.46E-05 | NUAK2 | chr1 | protein_coding |
| ENSG00000235169.11 | 10.7960356 | 1.58470636 | 0.4871149 | 3.25324964 | 0.00114093 | 0.0055732 | SMIM1 | chr1 | protein_coding |
| ENSG00000168135.4 | 38.9503325 | 1.41074986 | 0.26559173 | 5.31172365 | 1.09E-07 | 1.58E-06 | KCNJ4 | chr22 | protein_coding |
| ENSG00000258620.2 | 10.3993716 | 2.18348439 | 0.63847625 | 3.41983653 | 0.00062659 | 0.00333801 | ENSG00000258620 | chr14 | lncRNA |
| ENSG00000224790.3 | 38.6971835 | -1.0315426 | 0.27504404 | -3.7504635 | 0.00017651 | 0.00112922 | HLCS-AS1 | chr21 | lncRNA |
| ENSG00000253239.1 | 14.3682051 | 1.23954188 | 0.42743319 | 2.89996636 | 0.00373203 | 0.01529914 | IGLVI-70 | chr22 | IG_V_pseudogene |
| ENSG00000259459.6 | 2.69486657 | 3.23940373 | 1.29543322 | 2.50063351 | 0.01239714 | 0.0416566 | LINC02568 | chr15 | lncRNA |
| ENSG00000233912.1 | 21.0486239 | 1.06876087 | 0.3591503 | 2.97580389 | 0.00292222 | 0.01242575 | ENSG00000233912 | chr3 | lncRNA |
| ENSG00000287670.1 | 24.6359124 | 1.43085055 | 0.33607339 | 4.25755387 | 2.07E-05 | 0.00017104 | ENSG00000287670 | chr2 | lncRNA |
| ENSG00000223969.7 | 14.3967199 | 1.23415113 | 0.46799427 | 2.63710735 | 0.00836164 | 0.03010417 | ENSG00000223969 | chr7 | lncRNA |
| ENSG00000165879.9 | 59.9914643 | 1.51377443 | 0.24412032 | 6.20093578 | 5.61E-10 | 1.36E-08 | FRAT1 | chr10 | protein_coding |
| ENSG00000185267.10 | 22.584345 | 1.17264945 | 0.33529092 | 3.49740887 | 0.0004698 | 0.00262254 | CDNF | chr10 | protein_coding |
| ENSG00000187595.18 | 23.1239438 | 1.13983994 | 0.34235899 | 3.32937051 | 0.00087043 | 0.00440048 | ZNF385C | chr17 | protein_coding |
| ENSG00000126561.18 | 23.0866719 | 1.05121813 | 0.33388841 | 3.14841157 | 0.0016416 | 0.00762878 | STAT5A | chr17 | protein_coding |
| ENSG00000260979.1 | 19.3468429 | -1.1089771 | 0.40202405 | -2.7584846 | 0.005807 | 0.02219445 | USP7-AS1 | chr16 | lncRNA |
| ENSG00000277688.1 | 9.69521648 | -1.3316258 | 0.5176679 | -2.5723555 | 0.01010091 | 0.03538974 | ENSG00000277688 | chr17 | lncRNA |
| ENSG00000130158.14 | 22.7946178 | 1.55024652 | 0.36539876 | 4.24261566 | 2.21E-05 | 0.00018177 | DOCK6 | chr19 | protein_coding |
| ENSG00000239713.9 | 31.7245693 | 1.14554162 | 0.31957818 | 3.58454269 | 0.00033767 | 0.00197366 | APOBEC3G | chr22 | protein_coding |
| ENSG00000230798.7 | 52.9629719 | 1.46998318 | 0.22968692 | 6.39994282 | 1.55E-10 | 4.24E-09 | FOXD3-AS1 | chr1 | lncRNA |
| ENSG00000204540.11 | 5.17256148 | 1.98305361 | 0.80228468 | 2.47175805 | 0.01344505 | 0.04455374 | PSORS1C1 | chr6 | protein_coding |
| ENSG00000172403.11 | 17.2440495 | 1.18367237 | 0.44940878 | 2.63384347 | 0.00844244 | 0.03035368 | SYNPO2 | chr4 | protein_coding |
| ENSG00000006468.15 | 57.3600247 | 1.07884927 | 0.21192775 | 5.09064646 | 3.57E-07 | 4.65E-06 | ETV1 | chr7 | protein_coding |
| ENSG00000291097.1 | 21.6112458 | 1.0304324 | 0.35865437 | 2.87305132 | 0.00406528 | 0.01645126 | HLA-L | chr6 | lncRNA |
| ENSG00000253666.4 | 99.8453539 | 1.01730059 | 0.20711044 | 4.9118749 | 9.02E-07 | 1.06E-05 | ENSG00000253666 | chr8 | lncRNA |
| ENSG00000261488.1 | 15.2148885 | 1.5671799 | 0.45573593 | 3.43878946 | 0.00058432 | 0.00315504 | TBILA | chr3 | lncRNA |
| ENSG00000269984.1 | 15.53253 | -1.0423105 | 0.41735187 | -2.4974383 | 0.01250942 | 0.04196043 | ENSG00000269984 | chr3 | lncRNA |
| ENSG00000090104.12 | 1011.48895 | 1.0646686 | 0.08679685 | 12.2662123 | 1.38E-34 | 1.04E-31 | RGS1 | chr1 | protein_coding |
| ENSG00000225079.2 | 2.99462425 | 2.78440686 | 1.12966241 | 2.46481323 | 0.01370847 | 0.04524909 | FTH1P22 | chr1 | processed_pseudogene |
| ENSG00000213262.3 | 16.2887925 | 1.34126123 | 0.424123 | 3.16243453 | 0.00156456 | 0.00732882 | VDAC2P3 | chr1 | processed_pseudogene |
| ENSG00000293439.1 | 4.58807116 | 2.57223428 | 0.96530407 | 2.66468811 | 0.00770598 | 0.0281079 | ENSG00000293439 | chr15 | lncRNA |
| ENSG00000175906.5 | 19.5128956 | 2.27118557 | 0.405056 | 5.60709025 | 2.06E-08 | 3.54E-07 | ARL4D | chr17 | protein_coding |
| ENSG00000054179.12 | 16.9975592 | 1.89808757 | 0.48250438 | 3.93382453 | 8.36E-05 | 0.00058119 | ENTPD2 | chr9 | protein_coding |
| ENSG00000271387.1 | 13.3511389 | 1.17758738 | 0.43521435 | 2.70576413 | 0.00681474 | 0.02536098 | C1orf21-DT | chr1 | lncRNA |
| ENSG00000128917.8 | 20.8410643 | 1.00718619 | 0.33953794 | 2.9663436 | 0.00301364 | 0.01276819 | DLL4 | chr15 | protein_coding |
| ENSG00000231329.8 | 2.62414881 | 4.00233141 | 1.3346566 | 2.99877243 | 0.0027107 | 0.01165544 | ENSG00000231329 | chr6 | lncRNA |
| ENSG00000142408.7 | 6.26729806 | 2.13173099 | 0.72523774 | 2.93935474 | 0.00328896 | 0.0137771 | CACNG8 | chr19 | protein_coding |
| ENSG00000005001.10 | 11.7268124 | 1.50283254 | 0.52328669 | 2.87191047 | 0.00407999 | 0.0164918 | PRSS22 | chr16 | protein_coding |
| ENSG00000267457.1 | 4.8133123 | -2.0103459 | 0.79465166 | -2.5298455 | 0.01141128 | 0.03895778 | ENSG00000267457 | chr17 | lncRNA |
| ENSG00000180251.5 | 2.86889206 | 3.35463937 | 1.27305724 | 2.63510489 | 0.00841113 | 0.03025141 | SLC9A4 | chr2 | protein_coding |
| ENSG00000106278.12 | 37.7052601 | 1.09716295 | 0.25759923 | 4.25918561 | 2.05E-05 | 0.00016987 | PTPRZ1 | chr7 | protein_coding |
| ENSG00000289635.2 | 46.3655075 | 1.04924067 | 0.23103263 | 4.54152581 | 5.58E-06 | 5.36E-05 | ENSG00000289635 | chr11 | lncRNA |
| ENSG00000166592.13 | 115.244697 | 2.3138275 | 0.19512023 | 11.8584704 | 1.94E-32 | 1.03E-29 | RRAD | chr16 | protein_coding |
| ENSG00000160791.14 | 24.6461828 | 1.14215476 | 0.31802802 | 3.59136518 | 0.00032895 | 0.00193072 | CCR5 | chr3 | protein_coding |
| ENSG00000172638.13 | 50.1461561 | 1.212647 | 0.22775628 | 5.32431871 | 1.01E-07 | 1.49E-06 | EFEMP2 | chr11 | protein_coding |
| ENSG00000233251.8 | 52.9797825 | 1.04968526 | 0.2512419 | 4.17798655 | 2.94E-05 | 0.00023342 | ENSG00000233251 | chr2 | lncRNA |
| ENSG00000197467.17 | 5.91098216 | 2.07176524 | 0.72793204 | 2.84609707 | 0.00442587 | 0.01770096 | COL13A1 | chr10 | protein_coding |
| ENSG00000166816.15 | 54.1273107 | 1.82591762 | 0.24772013 | 7.37088922 | 1.69E-13 | 8.21E-12 | LDHD | chr16 | protein_coding |
| ENSG00000254870.5 | 10.526894 | 1.36328458 | 0.48525237 | 2.80943413 | 0.00496287 | 0.01947197 | ATP6V1G2-DDX39B | chr6 | protein_coding |
| ENSG00000214694.13 | 6.65268383 | 2.45698677 | 0.71742493 | 3.42473014 | 0.00061541 | 0.00328882 | ARHGEF33 | chr2 | protein_coding |
| ENSG00000205436.8 | 10.3155066 | 1.51145236 | 0.52721066 | 2.86688503 | 0.00414534 | 0.01672074 | EXOC3L4 | chr14 | protein_coding |
| ENSG00000154269.15 | 4.3661528 | 4.76475764 | 1.18693938 | 4.01432264 | 5.96E-05 | 0.00043252 | ENPP3 | chr6 | protein_coding |
| ENSG00000211670.2 | 32.7056161 | 1.10369276 | 0.28408564 | 3.8850706 | 0.0001023 | 0.0006949 | IGLV3-9 | chr22 | IG_V_gene |
| ENSG00000182240.16 | 17.5567913 | 1.27812226 | 0.38598548 | 3.31132215 | 0.00092856 | 0.00465207 | BACE2 | chr21 | protein_coding |
| ENSG00000158106.14 | 22.7917966 | 1.20515844 | 0.33913673 | 3.55360635 | 0.00037999 | 0.00217938 | RHPN1 | chr8 | protein_coding |
| ENSG00000168016.15 | 269.09907 | 1.19439335 | 0.12210351 | 9.78180994 | 1.35E-22 | 2.61E-20 | TRANK1 | chr3 | protein_coding |
| ENSG00000110237.5 | 37.3016934 | 1.18823026 | 0.27866287 | 4.26404222 | 2.01E-05 | 0.000167 | ARHGEF17 | chr11 | protein_coding |
| ENSG00000117525.14 | 8.63893496 | 1.70690118 | 0.61110216 | 2.79315194 | 0.00521972 | 0.020311 | F3 | chr1 | protein_coding |
| ENSG00000257622.1 | 12.8176229 | -1.1030168 | 0.44339914 | -2.4876386 | 0.01285943 | 0.04293664 | ENSG00000257622 | chr14 | lncRNA |
| ENSG00000113916.18 | 203.91677 | 1.07212781 | 0.12052094 | 8.8957806 | 5.80E-19 | 6.92E-17 | BCL6 | chr3 | protein_coding |
| ENSG00000117245.13 | 8.95830128 | 1.97995259 | 0.57140033 | 3.4650883 | 0.00053006 | 0.00290513 | KIF17 | chr1 | protein_coding |
| ENSG00000173227.14 | 46.8272861 | 1.95064024 | 0.33003591 | 5.91038793 | 3.41E-09 | 6.92E-08 | SYT12 | chr11 | protein_coding |
| ENSG00000114200.10 | 31.672331 | 1.20297747 | 0.28143348 | 4.27446471 | 1.92E-05 | 0.00015994 | BCHE | chr3 | protein_coding |
| ENSG00000265784.1 | 44.9674455 | -1.1417818 | 0.23578389 | -4.8424929 | 1.28E-06 | 1.45E-05 | ENSG00000265784 | chr17 | lncRNA |
| ENSG00000169136.13 | 3654.08936 | -1.1022618 | 0.06548291 | -16.832815 | 1.40E-63 | 4.23E-60 | ATF5 | chr19 | protein_coding |
| ENSG00000213213.14 | 62.1100159 | 1.20508251 | 0.23577221 | 5.11121528 | 3.20E-07 | 4.23E-06 | CCDC183 | chr9 | protein_coding |
| ENSG00000171798.19 | 10.1535957 | 2.09293968 | 0.58241267 | 3.59356826 | 0.00032618 | 0.00191558 | KNDC1 | chr10 | protein_coding |
| ENSG00000010818.11 | 337.671508 | 1.11762416 | 0.10101552 | 11.0638861 | 1.88E-28 | 7.08E-26 | HIVEP2 | chr6 | protein_coding |
| ENSG00000289614.2 | 13.1451381 | 1.41241257 | 0.45961026 | 3.07306581 | 0.00211872 | 0.00946074 | ENSG00000289614 | chr1 | lncRNA |
| ENSG00000136689.20 | 21.2945881 | 1.46886403 | 0.36454692 | 4.02928664 | 5.59E-05 | 0.00040884 | IL1RN | chr2 | protein_coding |
| ENSG00000089012.14 | 18.2927005 | 2.24465593 | 0.43948438 | 5.10747606 | 3.26E-07 | 4.30E-06 | SIRPG | chr20 | protein_coding |
| ENSG00000165140.12 | 12.2776247 | 1.74918855 | 0.50393996 | 3.47102574 | 0.00051847 | 0.00284756 | FBP1 | chr9 | protein_coding |
| ENSG00000183762.13 | 10.9329515 | 2.11014436 | 0.61311993 | 3.44165024 | 0.00057818 | 0.00312906 | KREMEN1 | chr22 | protein_coding |
| ENSG00000241644.2 | 17.884639 | 1.74342336 | 0.40919797 | 4.26058656 | 2.04E-05 | 0.0001692 | INMT | chr7 | protein_coding |
| ENSG00000116981.4 | 5.85418834 | 1.81884138 | 0.68098379 | 2.67090259 | 0.00756476 | 0.02769809 | NT5C1A | chr1 | protein_coding |
| ENSG00000184363.10 | 17.8804193 | 1.19753593 | 0.42845738 | 2.79499428 | 0.00519006 | 0.02022381 | PKP3 | chr11 | protein_coding |
| ENSG00000107331.18 | 40.1541065 | 1.03712031 | 0.26384351 | 3.93081613 | 8.47E-05 | 0.00058737 | ABCA2 | chr9 | protein_coding |
| ENSG00000077943.8 | 210.253149 | -1.1166843 | 0.1202759 | -9.2843557 | 1.63E-20 | 2.44E-18 | ITGA8 | chr10 | protein_coding |
| ENSG00000134278.16 | 32.6383883 | 1.37718737 | 0.29912643 | 4.60403103 | 4.14E-06 | 4.10E-05 | SPIRE1 | chr18 | protein_coding |
| ENSG00000167723.15 | 385.871777 | -1.0667881 | 0.09255633 | -11.525826 | 9.78E-31 | 4.21E-28 | TRPV3 | chr17 | protein_coding |
| ENSG00000269755.1 | 6.12413552 | 1.91066568 | 0.69726224 | 2.7402397 | 0.00613944 | 0.02323281 | ENSG00000269755 | chr19 | protein_coding |
| ENSG00000143847.16 | 67.072089 | 1.37370396 | 0.23515868 | 5.84160427 | 5.17E-09 | 1.00E-07 | PPFIA4 | chr1 | protein_coding |
| ENSG00000181322.15 | 21.3417986 | 1.14274504 | 0.34913101 | 3.27311239 | 0.0010637 | 0.00524585 | NME9 | chr3 | protein_coding |
| ENSG00000169129.16 | 12.9952556 | 1.61142492 | 0.46846082 | 3.43982855 | 0.00058208 | 0.00314456 | AFAP1L2 | chr10 | protein_coding |
| ENSG00000229191.1 | 19.973745 | 1.48384875 | 0.3974415 | 3.73350226 | 0.00018884 | 0.00119757 | ENSG00000229191 | chr1 | lncRNA |
| ENSG00000213889.11 | 68.7938423 | 1.26982269 | 0.21124411 | 6.01116268 | 1.84E-09 | 3.95E-08 | PPM1N | chr19 | protein_coding |
| ENSG00000262172.1 | 20.5817899 | -1.2932849 | 0.43019366 | -3.0062854 | 0.00264461 | 0.01142947 | ENSG00000262172 | chr17 | lncRNA |
| ENSG00000166165.14 | 703.386624 | 1.48345913 | 0.1085559 | 13.6653944 | 1.63E-42 | 2.30E-39 | CKB | chr14 | protein_coding |
| ENSG00000267737.1 | 12.0152553 | 1.37887184 | 0.45937024 | 3.0016569 | 0.00268515 | 0.01156442 | ENSG00000267737 | chr17 | lncRNA |
| ENSG00000167964.13 | 90.5221897 | 1.04731538 | 0.22347197 | 4.6865626 | 2.78E-06 | 2.88E-05 | RAB26 | chr16 | protein_coding |
| ENSG00000254231.3 | 14.0546558 | -1.349286 | 0.42641447 | -3.1642595 | 0.00155478 | 0.00728786 | WWP1-AS1 | chr8 | lncRNA |
| ENSG00000225177.6 | 22.4250222 | 1.36770242 | 0.33152754 | 4.12545644 | 3.70E-05 | 0.00028467 | NHSL1-AS1 | chr6 | lncRNA |
| ENSG00000124575.7 | 12.298017 | 1.20238039 | 0.47143803 | 2.55045271 | 0.01075831 | 0.03716124 | H1-3 | chr6 | protein_coding |
| ENSG00000165801.10 | 39.0345861 | 1.84741921 | 0.34198882 | 5.40198722 | 6.59E-08 | 1.01E-06 | ARHGEF40 | chr14 | protein_coding |
| ENSG00000128242.13 | 13.7808704 | 1.30479831 | 0.43440883 | 3.00361832 | 0.0026679 | 0.01150846 | GAL3ST1 | chr22 | protein_coding |
| ENSG00000214510.10 | 4.07846936 | 2.40852264 | 0.96550946 | 2.49456142 | 0.01261129 | 0.04222706 | SPINK13 | chr5 | protein_coding |
| ENSG00000242960.1 | 10.9327788 | -1.2421029 | 0.47775037 | -2.5998993 | 0.00932511 | 0.03305436 | FTH1P23 | chr3 | processed_pseudogene |
| ENSG00000162490.7 | 11.9112538 | 1.27696115 | 0.46971142 | 2.71860787 | 0.00655573 | 0.02454416 | DRAXIN | chr1 | protein_coding |
| ENSG00000165300.8 | 50.5040693 | 1.07049771 | 0.24250609 | 4.41431262 | 1.01E-05 | 9.08E-05 | SLITRK5 | chr13 | protein_coding |
| ENSG00000169862.21 | 6.66538528 | 1.89197089 | 0.65875907 | 2.87202253 | 0.00407854 | 0.01648911 | CTNND2 | chr5 | protein_coding |
| ENSG00000121797.10 | 60.7569366 | 1.49047043 | 0.22852415 | 6.52215709 | 6.93E-11 | 2.04E-09 | CCRL2 | chr3 | protein_coding |
| ENSG00000104833.12 | 48.0378457 | 2.67148313 | 0.30688494 | 8.70516211 | 3.17E-18 | 3.38E-16 | TUBB4A | chr19 | protein_coding |
| ENSG00000142552.8 | 44.4538943 | 2.31986518 | 0.28364248 | 8.17883555 | 2.87E-16 | 2.25E-14 | RCN3 | chr19 | protein_coding |
| ENSG00000171914.17 | 53.8571684 | 1.02646272 | 0.24828659 | 4.13418505 | 3.56E-05 | 0.00027588 | TLN2 | chr15 | protein_coding |
| ENSG00000271605.6 | 18.1370479 | 2.08254441 | 0.39871257 | 5.22317224 | 1.76E-07 | 2.44E-06 | MILR1 | chr17 | protein_coding |
| ENSG00000146166.17 | 31.2289768 | 1.55079081 | 0.31302289 | 4.95424089 | 7.26E-07 | 8.82E-06 | LGSN | chr6 | protein_coding |
| ENSG00000287878.1 | 4.54898052 | 2.27592578 | 0.83510573 | 2.72531453 | 0.00642403 | 0.0241281 | ENSG00000287878 | chr3 | lncRNA |
| ENSG00000218416.4 | 31.6956296 | -1.5561305 | 0.29705841 | -5.2384666 | 1.62E-07 | 2.28E-06 | GPC1-AS1 | chr2 | lncRNA |
| ENSG00000124839.13 | 22.1647573 | 1.25959503 | 0.34516032 | 3.64930426 | 0.00026295 | 0.00159302 | RAB17 | chr2 | protein_coding |
| ENSG00000100024.15 | 50.006751 | 1.79907816 | 0.2601164 | 6.91643483 | 4.63E-12 | 1.74E-10 | UPB1 | chr22 | protein_coding |
| ENSG00000211452.12 | 6.44943194 | 2.17039667 | 0.7300598 | 2.97290261 | 0.00294998 | 0.01253623 | DIO1 | chr1 | protein_coding |
| ENSG00000123329.20 | 180.063653 | -2.3098009 | 0.38964085 | -5.9280254 | 3.07E-09 | 6.29E-08 | ARHGAP9 | chr12 | protein_coding |
| ENSG00000170231.16 | 73.2160701 | 1.40900485 | 0.19557996 | 7.20423941 | 5.84E-13 | 2.57E-11 | FABP6 | chr5 | protein_coding |
| ENSG00000234975.6 | 67.3768684 | -1.045321 | 0.19754543 | -5.2915475 | 1.21E-07 | 1.74E-06 | FTH1P2 | chr1 | processed_pseudogene |
| ENSG00000080618.17 | 9.65148598 | 1.77394887 | 0.53672631 | 3.30512746 | 0.00094933 | 0.00473925 | CPB2 | chr13 | protein_coding |
| ENSG00000261838.5 | 38.4837535 | 1.00088127 | 0.2633443 | 3.8006566 | 0.00014431 | 0.00094531 | ENSG00000261838 | chr16 | lncRNA |
| ENSG00000148737.18 | 14.2771154 | 1.36896277 | 0.4703987 | 2.91021804 | 0.00361177 | 0.01487256 | TCF7L2 | chr10 | protein_coding |
| ENSG00000163082.10 | 16.6765782 | -1.07926 | 0.40592111 | -2.6587926 | 0.00784212 | 0.0285404 | SGPP2 | chr2 | protein_coding |
| ENSG00000144730.19 | 14.3844294 | 1.32377462 | 0.42235526 | 3.13426812 | 0.00172283 | 0.00794506 | IL17RD | chr3 | protein_coding |
| ENSG00000007402.12 | 18.020324 | 1.33987994 | 0.3890037 | 3.44438871 | 0.00057235 | 0.00309992 | CACNA2D2 | chr3 | protein_coding |
| ENSG00000279154.1 | 20.2138317 | 1.32029318 | 0.36888768 | 3.57911977 | 0.00034475 | 0.0020084 | ENSG00000279154 | chr9 | TEC |
| ENSG00000152154.12 | 102.819674 | 1.29539691 | 0.18044803 | 7.17878099 | 7.03E-13 | 3.04E-11 | TMEM178A | chr2 | protein_coding |
| ENSG00000163517.15 | 62.4873101 | 1.2374102 | 0.21871259 | 5.65770003 | 1.53E-08 | 2.69E-07 | HDAC11 | chr3 | protein_coding |
| ENSG00000118523.6 | 31.6669409 | 1.74343197 | 0.30481037 | 5.71972651 | 1.07E-08 | 1.92E-07 | CCN2 | chr6 | protein_coding |
| ENSG00000145685.14 | 16.2921732 | 1.39202349 | 0.4100741 | 3.39456575 | 0.00068738 | 0.00360364 | LHFPL2 | chr5 | protein_coding |
| ENSG00000196576.16 | 40.6493691 | 1.02228591 | 0.2666275 | 3.83413524 | 0.00012601 | 0.00083999 | PLXNB2 | chr22 | protein_coding |
| ENSG00000244414.8 | 21.3926697 | 1.15059054 | 0.3405812 | 3.3783149 | 0.00072932 | 0.00379524 | CFHR1 | chr1 | protein_coding |
| ENSG00000233728.1 | 61.6403845 | -1.1716819 | 0.26778396 | -4.3754745 | 1.21E-05 | 0.00010686 | ENSG00000233728 | chr1 | lncRNA |
| ENSG00000156966.7 | 19.5756905 | 1.96813595 | 0.49814042 | 3.95096621 | 7.78E-05 | 0.0005454 | B3GNT7 | chr2 | protein_coding |
| ENSG00000253736.5 | 10.2536389 | 1.77707016 | 0.52870101 | 3.36120062 | 0.00077604 | 0.00399801 | ENSG00000253736 | chr5 | lncRNA |
| ENSG00000273820.2 | 12.6188881 | 1.33319128 | 0.44715766 | 2.9814792 | 0.0028686 | 0.01222236 | USP27X | chrX | protein_coding |
| ENSG00000219607.4 | 43.6535777 | 1.53295891 | 0.25239598 | 6.07362658 | 1.25E-09 | 2.77E-08 | PPP1R3G | chr6 | protein_coding |
| ENSG00000138735.16 | 81.4425967 | 1.02182785 | 0.19006228 | 5.37627902 | 7.60E-08 | 1.15E-06 | PDE5A | chr4 | protein_coding |
| ENSG00000127946.17 | 20.4728388 | 1.21373598 | 0.38337641 | 3.16591198 | 0.00154598 | 0.00725676 | HIP1 | chr7 | protein_coding |
| ENSG00000136160.17 | 3380.8394 | -1.4029409 | 0.08166777 | -17.178637 | 3.84E-66 | 1.62E-62 | EDNRB | chr13 | protein_coding |
| ENSG00000168427.9 | 13.6026159 | 1.46440532 | 0.46627912 | 3.14061955 | 0.00168591 | 0.00779807 | KLHL30 | chr2 | protein_coding |
| ENSG00000100342.22 | 71.8704529 | 1.06312936 | 0.24815292 | 4.28417018 | 1.83E-05 | 0.00015428 | APOL1 | chr22 | protein_coding |
| ENSG00000162755.14 | 21.1234497 | 1.08896507 | 0.34419267 | 3.16382414 | 0.00155711 | 0.00729553 | KLHDC9 | chr1 | protein_coding |
| ENSG00000135925.9 | 753.082026 | -1.083827 | 0.08090679 | -13.395995 | 6.38E-41 | 7.93E-38 | WNT10A | chr2 | protein_coding |
| ENSG00000100453.14 | 34.0913976 | 3.70239572 | 0.41413579 | 8.94005261 | 3.89E-19 | 4.72E-17 | GZMB | chr14 | protein_coding |
| ENSG00000261822.1 | 32.2822225 | -1.1538465 | 0.31104684 | -3.7095587 | 0.00020762 | 0.00130068 | ENSG00000261822 | chr15 | lncRNA |
| ENSG00000171368.12 | 49.6946314 | 1.54160287 | 0.23791668 | 6.47959127 | 9.20E-11 | 2.65E-09 | TPPP | chr5 | protein_coding |
| ENSG00000204634.13 | 55.380604 | 1.22518276 | 0.22888754 | 5.35277174 | 8.66E-08 | 1.30E-06 | TBC1D8 | chr2 | protein_coding |
| ENSG00000028137.19 | 10.6646554 | 1.54688878 | 0.56757712 | 2.72542482 | 0.00642188 | 0.02412481 | TNFRSF1B | chr1 | protein_coding |
| ENSG00000161609.10 | 10.7319451 | 1.41867399 | 0.51154281 | 2.77332409 | 0.00554868 | 0.0213105 | KASH5 | chr19 | protein_coding |
| ENSG00000230316.8 | 53.6918456 | 1.13250032 | 0.23599273 | 4.79887793 | 1.60E-06 | 1.76E-05 | FEZF1-AS1 | chr7 | lncRNA |
| ENSG00000010319.7 | 32.8687081 | 1.24543085 | 0.27276392 | 4.56596628 | 4.97E-06 | 4.82E-05 | SEMA3G | chr3 | protein_coding |
| ENSG00000177337.10 | 10.9316917 | 1.61008068 | 0.51969857 | 3.09810486 | 0.00194762 | 0.00881609 | DLGAP1-AS1 | chr18 | lncRNA |
| ENSG00000163328.14 | 164.724548 | 1.13622701 | 0.1419264 | 8.00574803 | 1.19E-15 | 8.25E-14 | GPR155 | chr2 | protein_coding |
| ENSG00000225370.1 | 17.2128693 | 1.02473343 | 0.39385515 | 2.60180277 | 0.00927352 | 0.03291015 | ENSG00000225370 | chr19 | unprocessed_pseudogene |
| ENSG00000215045.9 | 6.12320646 | 2.09974251 | 0.78444552 | 2.6767219 | 0.00743463 | 0.02730688 | GRID2IP | chr7 | protein_coding |
| ENSG00000276075.3 | 30.2957388 | -1.3106043 | 0.31003077 | -4.2273361 | 2.36E-05 | 0.00019268 | ENSG00000276075 | chr16 | lncRNA |
| ENSG00000258884.3 | 33.9710605 | 1.00557135 | 0.28956867 | 3.47265247 | 0.00051534 | 0.00283331 | LINC02321 | chr14 | lncRNA |
| ENSG00000262712.1 | 65.3401467 | -1.0718703 | 0.20510692 | -5.2259101 | 1.73E-07 | 2.42E-06 | ENSG00000262712 | chr16 | lncRNA |
| ENSG00000260293.2 | 37.0600963 | -1.0801812 | 0.29619503 | -3.646858 | 0.00026547 | 0.00160358 | ENSG00000260293 | chr16 | lncRNA |
| ENSG00000198753.12 | 12.1983666 | 1.21590445 | 0.47848995 | 2.54112851 | 0.01104953 | 0.03794367 | PLXNB3 | chrX | protein_coding |
| ENSG00000232044.8 | 12.6567381 | 1.34829454 | 0.45706282 | 2.94991081 | 0.00317866 | 0.01337924 | SILC1 | chr2 | lncRNA |
| ENSG00000136002.21 | 4.60296517 | 2.98050511 | 0.96085709 | 3.10192342 | 0.00192268 | 0.00871999 | ARHGEF4 | chr2 | protein_coding |
| ENSG00000155893.13 | 20.4132547 | 2.10623693 | 0.39623765 | 5.31559015 | 1.06E-07 | 1.55E-06 | PXYLP1 | chr3 | protein_coding |
| ENSG00000107611.16 | 20.0367446 | 1.00873932 | 0.37879783 | 2.66300184 | 0.0077447 | 0.02822964 | CUBN | chr10 | protein_coding |
| ENSG00000129990.15 | 13.1015044 | 1.62370556 | 0.45722056 | 3.55125231 | 0.0003834 | 0.00219658 | SYT5 | chr19 | protein_coding |
| ENSG00000140519.14 | 11.3361775 | -1.2575626 | 0.48037694 | -2.6178662 | 0.00884815 | 0.03159176 | RHCG | chr15 | protein_coding |
| ENSG00000144893.13 | 7.76612694 | 1.49944072 | 0.57811594 | 2.59366782 | 0.00949582 | 0.03354119 | MED12L | chr3 | protein_coding |
| ENSG00000178075.20 | 59.5579739 | 1.09561078 | 0.23617174 | 4.63904258 | 3.50E-06 | 3.51E-05 | GRAMD1C | chr3 | protein_coding |
| ENSG00000289697.1 | 129.720871 | 1.0581767 | 0.14747235 | 7.17542461 | 7.21E-13 | 3.11E-11 | ENSG00000289697 | chr1 | protein_coding |
| ENSG00000185340.16 | 34.5588432 | 1.00416211 | 0.26505809 | 3.78846045 | 0.00015158 | 0.00098803 | GAS2L1 | chr22 | protein_coding |
| ENSG00000231233.3 | 9.78187724 | 1.6734487 | 0.5444815 | 3.07347208 | 0.00211583 | 0.00945186 | CFAP58-DT | chr10 | lncRNA |
| ENSG00000179456.12 | 22.459852 | 1.23497289 | 0.33404065 | 3.6970737 | 0.0002181 | 0.00135946 | ZBTB18 | chr1 | protein_coding |
| ENSG00000253368.4 | 21.5900297 | 1.88677383 | 0.39031123 | 4.83402395 | 1.34E-06 | 1.51E-05 | TRNP1 | chr1 | protein_coding |
| ENSG00000129353.15 | 282.554129 | 1.12836229 | 0.10916677 | 10.3361334 | 4.84E-25 | 1.23E-22 | SLC44A2 | chr19 | protein_coding |
| ENSG00000236136.1 | 9.33072704 | 2.06437752 | 0.56244112 | 3.67038871 | 0.00024218 | 0.00148373 | ADORA2BP1 | chr1 | processed_pseudogene |
| ENSG00000197093.11 | 9.47206294 | 1.74347411 | 0.56824313 | 3.06818335 | 0.00215364 | 0.00958022 | GAL3ST4 | chr7 | protein_coding |
| ENSG00000113263.14 | 12.0028732 | 2.90150942 | 0.58246163 | 4.98146019 | 6.31E-07 | 7.78E-06 | ITK | chr5 | protein_coding |
| ENSG00000133317.16 | 9.68913985 | 1.46037542 | 0.55242644 | 2.64356538 | 0.00820379 | 0.02965219 | LGALS12 | chr11 | protein_coding |
| ENSG00000105668.8 | 15.9843939 | 1.14138717 | 0.41828664 | 2.72872012 | 0.00635806 | 0.02393144 | UPK1A | chr19 | protein_coding |
| ENSG00000110436.13 | 13.8817083 | 1.89182414 | 0.48511919 | 3.89970995 | 9.63E-05 | 0.00065951 | SLC1A2 | chr11 | protein_coding |
| ENSG00000073792.16 | 42.2500543 | 1.30102449 | 0.30492085 | 4.26676128 | 1.98E-05 | 0.00016524 | IGF2BP2 | chr3 | protein_coding |
| ENSG00000171551.12 | 4.68433582 | 5.63216779 | 1.20661787 | 4.66773112 | 3.05E-06 | 3.12E-05 | ECEL1 | chr2 | protein_coding |
| ENSG00000185100.12 | 57.8542381 | 1.26197819 | 0.25724854 | 4.90567688 | 9.31E-07 | 1.09E-05 | ADSS1 | chr14 | protein_coding |
| ENSG00000273243.1 | 8.66285991 | -1.5744192 | 0.58592988 | -2.6870437 | 0.00720875 | 0.0265929 | ENSG00000273243 | chr22 | lncRNA |
| ENSG00000106665.16 | 18.6664482 | 1.2435793 | 0.39825997 | 3.12253154 | 0.00179303 | 0.00823101 | CLIP2 | chr7 | protein_coding |
| ENSG00000181790.13 | 6.26616214 | 1.99157099 | 0.71486134 | 2.78595426 | 0.00533704 | 0.02066684 | ADGRB1 | chr8 | protein_coding |
| ENSG00000205403.15 | 36.7956951 | 1.47527198 | 0.26522899 | 5.56225772 | 2.66E-08 | 4.46E-07 | CFI | chr4 | protein_coding |
| ENSG00000087116.17 | 5.1713773 | -2.3483778 | 0.844299 | -2.7814528 | 0.00541162 | 0.02089961 | ADAMTS2 | chr5 | protein_coding |
| ENSG00000179921.15 | 11.2564811 | -1.1637018 | 0.47297731 | -2.4603756 | 0.01387917 | 0.04570535 | GPBAR1 | chr2 | protein_coding |
| ENSG00000203721.8 | 4.42272014 | 2.93531866 | 0.93661305 | 3.13397157 | 0.00172458 | 0.00794962 | LINC00862 | chr1 | lncRNA |
| ENSG00000167995.17 | 5440.86091 | -1.2047068 | 0.12571673 | -9.5827083 | 9.45E-22 | 1.62E-19 | BEST1 | chr11 | protein_coding |
| ENSG00000182459.5 | 235.415475 | 1.31319555 | 0.12072865 | 10.8772483 | 1.48E-27 | 4.96E-25 | TEX19 | chr17 | protein_coding |
| ENSG00000229525.1 | 11.2979099 | 2.39222548 | 0.5330303 | 4.48797282 | 7.19E-06 | 6.69E-05 | DNPEP-AS1 | chr2 | lncRNA |
| ENSG00000276509.1 | 14.7187572 | 1.11484607 | 0.43252293 | 2.57754213 | 0.00995057 | 0.0349545 | ENSG00000276509 | chr1 | lncRNA |
| ENSG00000178053.21 | 130.774706 | 1.02910198 | 0.1436214 | 7.16538026 | 7.76E-13 | 3.32E-11 | MLF1 | chr3 | protein_coding |
| ENSG00000151117.9 | 132.76764 | 1.0267525 | 0.1511726 | 6.79192192 | 1.11E-11 | 3.85E-10 | TMEM86A | chr11 | protein_coding |
| ENSG00000227252.2 | 83.3923233 | 1.14739682 | 0.18392846 | 6.23827763 | 4.42E-10 | 1.10E-08 | COPS8-DT | chr2 | lncRNA |
| ENSG00000285330.1 | 71.2070626 | 1.9518759 | 0.22254957 | 8.77052191 | 1.78E-18 | 1.98E-16 | ENSG00000285330 | chr4 | protein_coding |
| ENSG00000136717.15 | 149.488823 | -1.133936 | 0.17812926 | -6.3658045 | 1.94E-10 | 5.19E-09 | BIN1 | chr2 | protein_coding |
| ENSG00000279805.1 | 13.832961 | 1.50698956 | 0.44192929 | 3.41002421 | 0.00064957 | 0.00343962 | ENSG00000279805 | chr22 | TEC |
| ENSG00000187720.15 | 21.1094526 | 1.01169766 | 0.38255186 | 2.64460261 | 0.00817869 | 0.02958676 | THSD4 | chr15 | protein_coding |
| ENSG00000088899.16 | 95.2347442 | 1.01230661 | 0.20629841 | 4.90700153 | 9.25E-07 | 1.08E-05 | LZTS3 | chr20 | protein_coding |
| ENSG00000111087.10 | 68.4737912 | 1.61742845 | 0.226539 | 7.13973503 | 9.35E-13 | 3.96E-11 | GLI1 | chr12 | protein_coding |
| ENSG00000275743.1 | 6.32726227 | 1.64652129 | 0.64995169 | 2.53329795 | 0.01129949 | 0.03866366 | TRBV14 | chr7 | TR_V_gene |
| ENSG00000121964.15 | 24.5157616 | 1.24061291 | 0.35486286 | 3.49603476 | 0.00047223 | 0.0026326 | GTDC1 | chr2 | protein_coding |
| ENSG00000105672.15 | 23.632397 | -1.0881468 | 0.34940764 | -3.1142616 | 0.00184406 | 0.00843187 | ETV2 | chr19 | protein_coding |
| ENSG00000165905.18 | 37.2184578 | 1.04741695 | 0.26527993 | 3.94834607 | 7.87E-05 | 0.00055049 | LARGE2 | chr11 | protein_coding |
| ENSG00000286468.1 | 20.549953 | 1.21996493 | 0.37176853 | 3.2815175 | 0.0010325 | 0.00510855 | ENSG00000286468 | chr5 | lncRNA |
| ENSG00000184368.16 | 8.54940117 | 2.03986698 | 0.58059439 | 3.51341147 | 0.00044239 | 0.00248795 | MAP7D2 | chrX | protein_coding |
| ENSG00000054219.11 | 21.2477342 | 1.01537608 | 0.34102541 | 2.9774206 | 0.00290685 | 0.01237536 | LY75 | chr2 | protein_coding |
| ENSG00000080298.16 | 25.7295904 | 1.0827083 | 0.33174976 | 3.26362947 | 0.00109995 | 0.00539422 | RFX3 | chr9 | protein_coding |
| ENSG00000281491.1 | 6.93677562 | -1.6750413 | 0.65169455 | -2.5702859 | 0.01016146 | 0.03554744 | DNAJB5-DT | chr9 | lncRNA |
| ENSG00000142233.14 | 27.2960446 | 1.02842209 | 0.30611471 | 3.3595971 | 0.00078056 | 0.00401541 | NTN5 | chr19 | protein_coding |
| ENSG00000275552.1 | 12.1068212 | -1.3225804 | 0.45277853 | -2.9210317 | 0.00348874 | 0.0144562 | ENSG00000275552 | chr14 | lncRNA |
| ENSG00000197594.14 | 10.4650139 | 1.62147744 | 0.50615595 | 3.20351355 | 0.00135762 | 0.00648168 | ENPP1 | chr6 | protein_coding |
| ENSG00000261396.1 | 8.49120218 | -1.8915448 | 0.59975953 | -3.1538387 | 0.00161138 | 0.0075065 | ENSG00000261396 | chr16 | lncRNA |
| ENSG00000162645.13 | 56.491393 | 1.57337281 | 0.27727027 | 5.67450961 | 1.39E-08 | 2.45E-07 | GBP2 | chr1 | protein_coding |
| ENSG00000136830.12 | 180.509354 | 1.32950702 | 0.15021586 | 8.85064316 | 8.70E-19 | 1.01E-16 | NIBAN2 | chr9 | protein_coding |
| ENSG00000146776.15 | 8.55836031 | 1.67460501 | 0.56015991 | 2.98951241 | 0.00279423 | 0.01195371 | ATXN7L1 | chr7 | protein_coding |
| ENSG00000075673.12 | 29.4284623 | 1.02350781 | 0.31346711 | 3.26512027 | 0.00109418 | 0.00537302 | ATP12A | chr13 | protein_coding |
| ENSG00000120738.8 | 186.622631 | 1.36914433 | 0.17801092 | 7.69135005 | 1.46E-14 | 8.52E-13 | EGR1 | chr5 | protein_coding |
| ENSG00000163884.4 | 9.0213955 | 1.65388813 | 0.533042 | 3.10273514 | 0.00191741 | 0.00870359 | KLF15 | chr3 | protein_coding |
| ENSG00000235172.9 | 8.65743491 | 2.34799135 | 0.63911565 | 3.67381293 | 0.00023896 | 0.00146739 | LINC01366 | chr5 | lncRNA |
| ENSG00000171476.23 | 23.0757457 | 2.50099734 | 0.37592437 | 6.65292679 | 2.87E-11 | 9.23E-10 | HOPX | chr4 | protein_coding |
| ENSG00000261645.7 | 9.23401534 | 1.39979536 | 0.56060183 | 2.49695112 | 0.01252662 | 0.04199407 | DISC1FP1 | chr11 | lncRNA |
| ENSG00000170921.16 | 5.08843629 | 1.96805195 | 0.79109612 | 2.48775327 | 0.01285529 | 0.04292959 | TANC2 | chr17 | protein_coding |
| ENSG00000129474.17 | 43.5216993 | 1.06615045 | 0.25174189 | 4.23509353 | 2.28E-05 | 0.0001868 | AJUBA | chr14 | protein_coding |
| ENSG00000279364.1 | 59.3986346 | -1.0031147 | 0.24695446 | -4.0619422 | 4.87E-05 | 0.00036203 | ENSG00000279364 | chr15 | TEC |
| ENSG00000089250.20 | 10.505336 | 1.5626294 | 0.6216903 | 2.5135174 | 0.01195339 | 0.04042286 | NOS1 | chr12 | protein_coding |
| ENSG00000142920.18 | 8.06099099 | 1.68182208 | 0.58042239 | 2.89758307 | 0.0037605 | 0.01540689 | AZIN2 | chr1 | protein_coding |
| ENSG00000105605.8 | 30.1348889 | 2.93105495 | 0.41123723 | 7.12740665 | 1.02E-12 | 4.31E-11 | CACNG7 | chr19 | protein_coding |
| ENSG00000165959.12 | 47.3523076 | 1.05985619 | 0.25413758 | 4.17040324 | 3.04E-05 | 0.00023989 | CLMN | chr14 | protein_coding |
| ENSG00000231789.4 | 8.75699412 | 1.39545834 | 0.56546241 | 2.46781804 | 0.01359394 | 0.0449554 | PIK3CD-AS2 | chr1 | lncRNA |
| ENSG00000291338.1 | 90.2462862 | 1.4348982 | 0.17961163 | 7.98889353 | 1.36E-15 | 9.33E-14 | ENSG00000291338 | chr6 | TEC |
| ENSG00000169991.11 | 103.483447 | 1.0987517 | 0.17088075 | 6.42993254 | 1.28E-10 | 3.54E-09 | IFFO2 | chr1 | protein_coding |
| ENSG00000272808.4 | 7.45465667 | 1.6272808 | 0.65440458 | 2.48665864 | 0.01289491 | 0.04303526 | GCAWKR | chr15 | lncRNA |
| ENSG00000255521.3 | 48.2060981 | 1.22037434 | 0.33627228 | 3.62912561 | 0.00028438 | 0.00169889 | CD44-DT | chr11 | lncRNA |
| ENSG00000132965.10 | 118.866389 | 1.03699397 | 0.15433092 | 6.71928851 | 1.83E-11 | 6.05E-10 | ALOX5AP | chr13 | protein_coding |
| ENSG00000064042.18 | 41.0075096 | 1.04202209 | 0.2642764 | 3.9429252 | 8.05E-05 | 0.00056177 | LIMCH1 | chr4 | protein_coding |
| ENSG00000166016.6 | 42.6755407 | 1.30155669 | 0.25827067 | 5.0395064 | 4.67E-07 | 5.91E-06 | ABTB2 | chr11 | protein_coding |
| ENSG00000223764.2 | 6.40319432 | 1.68352932 | 0.65492253 | 2.57057781 | 0.0101529 | 0.03553362 | LINC02593 | chr1 | lncRNA |
| ENSG00000246526.3 | 17.6962095 | -1.2864694 | 0.37988148 | -3.386502 | 0.0007079 | 0.00369563 | LINC02481 | chr4 | lncRNA |
| ENSG00000231023.9 | 24.1815075 | 1.25096457 | 0.33046316 | 3.78548878 | 0.00015341 | 0.00099899 | LINC00326 | chr6 | lncRNA |
| ENSG00000100362.13 | 6.58707473 | 2.4409839 | 0.71224076 | 3.42718929 | 0.00060986 | 0.00326457 | PVALB | chr22 | protein_coding |
| ENSG00000260615.1 | 16.1890316 | 1.31652797 | 0.40008801 | 3.29059591 | 0.00099975 | 0.00496982 | RPL23AP97 | chr13 | processed_pseudogene |
| ENSG00000122861.16 | 63.128842 | 1.33402114 | 0.22251806 | 5.99511394 | 2.03E-09 | 4.31E-08 | PLAU | chr10 | protein_coding |
| ENSG00000263235.1 | 40.1175447 | -1.2475508 | 0.26386719 | -4.7279496 | 2.27E-06 | 2.40E-05 | ENSG00000263235 | chr16 | lncRNA |
| ENSG00000102445.20 | 5.66334036 | 2.19348274 | 0.75641111 | 2.89985525 | 0.00373335 | 0.01530159 | RUBCNL | chr13 | protein_coding |
| ENSG00000179674.4 | 12.9449573 | 2.42142633 | 0.57416599 | 4.21729324 | 2.47E-05 | 0.00020008 | ARL14 | chr3 | protein_coding |
| ENSG00000020577.14 | 20.401205 | 1.05501119 | 0.36401915 | 2.89822992 | 0.00375275 | 0.01537813 | SAMD4A | chr14 | protein_coding |
| ENSG00000177455.15 | 12.8517324 | 1.92210554 | 0.56459403 | 3.40440284 | 0.00066309 | 0.00350241 | CD19 | chr16 | protein_coding |
| ENSG00000091986.16 | 181.667434 | 1.10442809 | 0.13104269 | 8.4280023 | 3.52E-17 | 3.13E-15 | CCDC80 | chr3 | protein_coding |
| ENSG00000169715.15 | 20.0771244 | -1.4507833 | 0.46172498 | -3.142094 | 0.00167744 | 0.00776456 | MT1E | chr16 | protein_coding |
| ENSG00000122574.12 | 208.929248 | 1.34578586 | 0.12522772 | 10.746709 | 6.14E-27 | 1.94E-24 | WIPF3 | chr7 | protein_coding |
| ENSG00000154146.13 | 25.9024058 | 1.04034526 | 0.31280332 | 3.32587668 | 0.00088141 | 0.00444371 | NRGN | chr11 | protein_coding |
| ENSG00000023171.20 | 24.0615113 | 1.88018362 | 0.34351512 | 5.47336504 | 4.42E-08 | 7.09E-07 | GRAMD1B | chr11 | protein_coding |
| ENSG00000152463.15 | 19.2456683 | 1.21922373 | 0.39049266 | 3.12227055 | 0.00179462 | 0.00823473 | OLAH | chr10 | protein_coding |
| ENSG00000287264.1 | 15.6935527 | 1.19900087 | 0.42887188 | 2.79570871 | 0.00517861 | 0.02019034 | ENSG00000287264 | chr11 | lncRNA |
| ENSG00000116983.13 | 195.981464 | 1.94123434 | 0.14566594 | 13.3266182 | 1.62E-40 | 1.63E-37 | HPCAL4 | chr1 | protein_coding |
| ENSG00000125148.7 | 18.6035217 | -2.1166913 | 0.58463101 | -3.6205594 | 0.00029397 | 0.00174676 | MT2A | chr16 | protein_coding |
| ENSG00000105767.3 | 36.7878761 | 1.71251243 | 0.30358088 | 5.64104176 | 1.69E-08 | 2.94E-07 | CADM4 | chr19 | protein_coding |
| ENSG00000132702.13 | 17.3487005 | 1.20302227 | 0.38000581 | 3.16579964 | 0.00154657 | 0.00725745 | HAPLN2 | chr1 | protein_coding |
| ENSG00000167191.12 | 57.0034945 | 1.34901388 | 0.27629968 | 4.88243004 | 1.05E-06 | 1.21E-05 | GPRC5B | chr16 | protein_coding |
| ENSG00000078579.9 | 26.3406646 | 1.09236361 | 0.32537542 | 3.35724068 | 0.00078725 | 0.00404486 | FGF20 | chr8 | protein_coding |
| ENSG00000149557.14 | 107.708927 | 1.68036261 | 0.17489201 | 9.60800085 | 7.40E-22 | 1.31E-19 | FEZ1 | chr11 | protein_coding |
| ENSG00000132026.15 | 298.553761 | 1.7708409 | 0.12608983 | 14.0442805 | 8.35E-45 | 1.47E-41 | RTBDN | chr19 | protein_coding |
| ENSG00000248309.9 | 69.3762507 | 1.2744425 | 0.24262251 | 5.25277922 | 1.50E-07 | 2.12E-06 | MEF2C-AS1 | chr5 | lncRNA |
| ENSG00000273783.2 | 46.8548996 | 1.03028331 | 0.25867839 | 3.98287355 | 6.81E-05 | 0.00048579 | GTF2A1-AS1 | chr14 | lncRNA |
| ENSG00000233081.1 | 7.50102407 | 2.66346201 | 0.68505987 | 3.88792592 | 0.0001011 | 0.00068722 | ENSG00000233081 | chr9 | lncRNA |
| ENSG00000144824.21 | 101.816633 | 1.17033602 | 0.17670346 | 6.62316395 | 3.52E-11 | 1.10E-09 | PHLDB2 | chr3 | protein_coding |
| ENSG00000115594.12 | 46.2683908 | 1.158924 | 0.24288673 | 4.77145873 | 1.83E-06 | 1.99E-05 | IL1R1 | chr2 | protein_coding |
| ENSG00000258675.2 | 5.93778352 | 2.2515928 | 0.73653664 | 3.0570004 | 0.00223564 | 0.00989908 | LINC02308 | chr14 | lncRNA |
| ENSG00000235643.2 | 5.8483482 | 2.46631268 | 0.7845179 | 3.14373028 | 0.00166809 | 0.00773208 | LINC01647 | chr1 | lncRNA |
| ENSG00000225062.2 | 3.22774744 | 2.86302003 | 1.12058157 | 2.5549412 | 0.01062057 | 0.0368059 | CATIP-AS1 | chr2 | lncRNA |
| ENSG00000197249.14 | 36.5373014 | 1.17539532 | 0.28277527 | 4.15664116 | 3.23E-05 | 0.00025262 | SERPINA1 | chr14 | protein_coding |
| ENSG00000130821.17 | 132.007979 | 1.08089052 | 0.13774017 | 7.84731496 | 4.25E-15 | 2.70E-13 | SLC6A8 | chrX | protein_coding |
| ENSG00000176845.13 | 14.4197538 | 2.57918522 | 0.50527162 | 5.10455194 | 3.32E-07 | 4.36E-06 | METRNL | chr17 | protein_coding |
| ENSG00000290056.1 | 48.9105962 | 1.7690489 | 0.26634818 | 6.6418659 | 3.10E-11 | 9.86E-10 | ENSG00000290056 | chr17 | lncRNA |
| ENSG00000266970.3 | 35.7376116 | 1.00830298 | 0.26452819 | 3.8117033 | 0.00013801 | 0.00091139 | SOCS3-DT | chr17 | lncRNA |
| ENSG00000259049.1 | 19.8318411 | -1.0035419 | 0.34576277 | -2.9024 | 0.00370315 | 0.01520144 | ENSG00000259049 | chr14 | lncRNA |
| ENSG00000270547.6 | 3.27581575 | 2.9068748 | 1.07867391 | 2.69485965 | 0.00704183 | 0.02610484 | LINC01235 | chr9 | lncRNA |
| ENSG00000153721.19 | 14.4998006 | 1.74417297 | 0.43619283 | 3.99862828 | 6.37E-05 | 0.00045813 | CNKSR3 | chr6 | protein_coding |
| ENSG00000135063.20 | 13.091562 | 1.27179744 | 0.44732657 | 2.84310732 | 0.0044676 | 0.01783324 | ENTREP1 | chr9 | protein_coding |
| ENSG00000109667.12 | 15.2741413 | -1.1811996 | 0.416647 | -2.8350128 | 0.00458238 | 0.01822938 | SLC2A9 | chr4 | protein_coding |
| ENSG00000120332.17 | 7.21783021 | 1.75813667 | 0.64112426 | 2.74227131 | 0.00610159 | 0.02311445 | TNN | chr1 | protein_coding |
| ENSG00000196503.5 | 7.24743552 | -1.4806734 | 0.61090445 | -2.4237397 | 0.01536161 | 0.04976216 | ARL9 | chr4 | protein_coding |
| ENSG00000110811.20 | 59.257257 | 1.60640389 | 0.21592169 | 7.43975228 | 1.01E-13 | 5.09E-12 | P3H3 | chr12 | protein_coding |
| ENSG00000179141.9 | 22.6964429 | -1.0711972 | 0.32990404 | -3.2469964 | 0.0011663 | 0.00568264 | MTUS2-AS1 | chr13 | lncRNA |
| ENSG00000181652.20 | 38.8703006 | 1.01032434 | 0.35979053 | 2.80809041 | 0.00498362 | 0.01953114 | ATG9B | chr7 | protein_coding |
| ENSG00000162599.18 | 40.945434 | 1.45378083 | 0.26934194 | 5.39752871 | 6.76E-08 | 1.03E-06 | NFIA | chr1 | protein_coding |
| ENSG00000115257.15 | 137.748024 | 1.17646836 | 0.17805319 | 6.60739835 | 3.91E-11 | 1.22E-09 | PCSK4 | chr19 | protein_coding |
| ENSG00000270959.2 | 17.2890569 | 1.63575354 | 0.39406735 | 4.15094913 | 3.31E-05 | 0.00025832 | LPP-AS2 | chr3 | lncRNA |
| ENSG00000185215.11 | 91.9934886 | 1.19270646 | 0.17720115 | 6.73080556 | 1.69E-11 | 5.66E-10 | TNFAIP2 | chr14 | protein_coding |
| ENSG00000174885.13 | 14.5631885 | 1.05851692 | 0.42621868 | 2.48350661 | 0.01300959 | 0.0433352 | NLRP6 | chr11 | protein_coding |
| ENSG00000136449.16 | 50.4930724 | 1.10714162 | 0.2389948 | 4.63249248 | 3.61E-06 | 3.62E-05 | MYCBPAP | chr17 | protein_coding |
| ENSG00000050730.16 | 116.731269 | 1.09352601 | 0.19266005 | 5.67593552 | 1.38E-08 | 2.44E-07 | TNIP3 | chr4 | protein_coding |
| ENSG00000169093.16 | 8.17001996 | 1.56535557 | 0.60110379 | 2.60413523 | 0.00921064 | 0.03271451 | ASMTL | chrX | protein_coding |
| ENSG00000167535.8 | 289.720721 | 1.14713079 | 0.11700318 | 9.80427016 | 1.08E-22 | 2.13E-20 | CACNB3 | chr12 | protein_coding |
| ENSG00000263986.1 | 56.4162497 | -1.0007716 | 0.22333141 | -4.4811053 | 7.43E-06 | 6.89E-05 | ENSG00000263986 | chr17 | lncRNA |
| ENSG00000168389.18 | 19.5009341 | 1.00365343 | 0.36690877 | 2.73543047 | 0.00622987 | 0.02350342 | MFSD2A | chr1 | protein_coding |
| ENSG00000287166.1 | 8.4390624 | 2.03631796 | 0.60346427 | 3.37438033 | 0.00073982 | 0.00383876 | ENSG00000287166 | chr8 | lncRNA |
| ENSG00000240350.3 | 13.1599583 | 1.41457241 | 0.43515064 | 3.25076489 | 0.00115095 | 0.00561693 | SOCAR | chr2 | lncRNA |
| ENSG00000172828.13 | 14.8832876 | 1.376523 | 0.4430397 | 3.10699694 | 0.00188998 | 0.00859572 | CES3 | chr16 | protein_coding |
| ENSG00000187688.15 | 18.0519228 | 1.55660396 | 0.39814114 | 3.90967877 | 9.24E-05 | 0.00063514 | TRPV2 | chr17 | protein_coding |
| ENSG00000166681.14 | 12.6934712 | 1.80122263 | 0.48478139 | 3.71553585 | 0.00020277 | 0.00127523 | BEX3 | chrX | protein_coding |
| ENSG00000248795.1 | 8.1931354 | 1.57467844 | 0.58970273 | 2.67029191 | 0.00757853 | 0.02773409 | ENSG00000248795 | chr4 | processed_pseudogene |
| ENSG00000188649.16 | 19.6908148 | 1.00064569 | 0.35138744 | 2.84769911 | 0.00440365 | 0.01763132 | CC2D2B | chr10 | protein_coding |
| ENSG00000133138.20 | 16.2398253 | 1.05605518 | 0.38988929 | 2.7086027 | 0.00675672 | 0.025185 | TBC1D8B | chrX | protein_coding |
| ENSG00000264548.1 | 25.2376561 | 1.1896741 | 0.33286375 | 3.57405726 | 0.00035149 | 0.00204202 | ENSG00000264548 | chr17 | lncRNA |
| ENSG00000196739.15 | 137.007284 | 1.54477574 | 0.17890849 | 8.63444641 | 5.90E-18 | 5.74E-16 | COL27A1 | chr9 | protein_coding |
| ENSG00000111261.14 | 21.0104413 | 1.05555852 | 0.35354912 | 2.9856064 | 0.00283017 | 0.01208057 | MANSC1 | chr12 | protein_coding |
| ENSG00000166317.12 | 26.5134522 | 1.35652395 | 0.33818186 | 4.01122623 | 6.04E-05 | 0.00043733 | SYNPO2L | chr10 | protein_coding |
| ENSG00000248809.6 | 4.53647738 | 2.30086778 | 0.89829755 | 2.56136487 | 0.01042618 | 0.03630505 | LINC01095 | chr4 | lncRNA |
| ENSG00000145358.6 | 17.7252792 | 1.83683583 | 0.45767895 | 4.01337191 | 5.99E-05 | 0.00043397 | DDIT4L | chr4 | protein_coding |
| ENSG00000123384.14 | 46.8030199 | 1.6580183 | 0.25413338 | 6.52420522 | 6.84E-11 | 2.02E-09 | LRP1 | chr12 | protein_coding |
| ENSG00000135423.13 | 29.0736675 | 1.04010005 | 0.3100576 | 3.35453818 | 0.00079498 | 0.00407431 | GLS2 | chr12 | protein_coding |
| ENSG00000137094.16 | 171.794422 | 1.05644426 | 0.13500847 | 7.82502226 | 5.08E-15 | 3.18E-13 | DNAJB5 | chr9 | protein_coding |
| ENSG00000125409.13 | 5.04009273 | 1.96660498 | 0.78983765 | 2.48988507 | 0.01277844 | 0.04272026 | TEKT3 | chr17 | protein_coding |
| ENSG00000214049.8 | 4.5206601 | 2.96902426 | 0.95087915 | 3.12239914 | 0.00179384 | 0.00823293 | UCA1 | chr19 | lncRNA |
| ENSG00000288681.1 | 26.9475717 | -1.2204261 | 0.45599024 | -2.6764304 | 0.0074411 | 0.02732542 | ENSG00000288681 | chr11 | protein_coding |
| ENSG00000224429.10 | 27.9697062 | 1.02659567 | 0.31662541 | 3.24230348 | 0.00118568 | 0.00576376 | LINC00539 | chr13 | lncRNA |
| ENSG00000168298.7 | 37.8708084 | 1.28268091 | 0.27227068 | 4.71105039 | 2.46E-06 | 2.59E-05 | H1-4 | chr6 | protein_coding |
| ENSG00000187583.11 | 31.958632 | 1.26826652 | 0.34119591 | 3.71712114 | 0.00020151 | 0.00126839 | PLEKHN1 | chr1 | protein_coding |
| ENSG00000179604.10 | 56.3555716 | 1.1182208 | 0.22914538 | 4.87996214 | 1.06E-06 | 1.22E-05 | CDC42EP4 | chr17 | protein_coding |
| ENSG00000198723.11 | 8.71772871 | 1.93827401 | 0.65889857 | 2.941688 | 0.00326429 | 0.01369275 | SAXO5 | chr19 | protein_coding |
| ENSG00000291133.1 | 45.6136333 | 1.27610681 | 0.25035191 | 5.09725212 | 3.45E-07 | 4.51E-06 | ENSG00000291133 | chr7 | lncRNA |
| ENSG00000105967.16 | 11.5954964 | 1.39730875 | 0.50026698 | 2.7931261 | 0.00522013 | 0.020311 | TFEC | chr7 | protein_coding |
| ENSG00000289497.2 | 9.71519654 | 2.55806621 | 0.59743637 | 4.28173831 | 1.85E-05 | 0.00015554 | ENSG00000289497 | chr5 | lncRNA |
| ENSG00000152229.18 | 212.229707 | 1.95323272 | 0.16465554 | 11.8625387 | 1.85E-32 | 1.00E-29 | PSTPIP2 | chr18 | protein_coding |
| ENSG00000160188.10 | 23.6122633 | 1.29549161 | 0.33304916 | 3.88979102 | 0.00010033 | 0.00068284 | RSPH1 | chr21 | protein_coding |
| ENSG00000267125.2 | 13.7608258 | -1.2650276 | 0.43882585 | -2.8827553 | 0.00394213 | 0.01603282 | ENSG00000267125 | chr19 | lncRNA |
| ENSG00000288018.3 | 27.9579808 | 1.2910837 | 0.34547298 | 3.73714811 | 0.00018612 | 0.00118411 | ENSG00000288018 | chr11 | lncRNA |
| ENSG00000168676.11 | 33.8701458 | 2.09933144 | 0.31999317 | 6.56055076 | 5.36E-11 | 1.63E-09 | KCTD19 | chr16 | protein_coding |
| ENSG00000179111.9 | 28.0431183 | 2.16407653 | 0.33881898 | 6.38711711 | 1.69E-10 | 4.59E-09 | HES7 | chr17 | protein_coding |
| ENSG00000255397.1 | 8.01565392 | 1.66561228 | 0.59881372 | 2.7815199 | 0.0054105 | 0.02089961 | NASPP1 | chr8 | processed_pseudogene |
| ENSG00000122417.16 | 18.8662512 | 1.31980827 | 0.36794136 | 3.58700713 | 0.0003345 | 0.00195728 | ODF2L | chr1 | protein_coding |
| ENSG00000100385.15 | 1367.20829 | 1.37704289 | 0.27167164 | 5.06877681 | 4.00E-07 | 5.16E-06 | IL2RB | chr22 | protein_coding |
| ENSG00000105376.5 | 238.193333 | 1.62674386 | 0.14722616 | 11.049285 | 2.21E-28 | 8.18E-26 | ICAM5 | chr19 | protein_coding |
| ENSG00000160471.13 | 17.776009 | 1.61422323 | 0.39295837 | 4.10787338 | 3.99E-05 | 0.00030459 | COX6B2 | chr19 | protein_coding |
| ENSG00000170289.13 | 25.2077394 | -1.0164894 | 0.31683436 | -3.2082676 | 0.00133537 | 0.00639138 | CNGB3 | chr8 | protein_coding |
| ENSG00000146755.11 | 32.1286634 | 1.00644653 | 0.31154308 | 3.23052121 | 0.00123565 | 0.00597777 | TRIM50 | chr7 | protein_coding |
| ENSG00000234807.8 | 5.84910451 | 2.44179399 | 0.7532227 | 3.24179555 | 0.00118779 | 0.00577271 | LINC01135 | chr1 | lncRNA |
| ENSG00000123358.20 | 29.0271059 | 1.54470077 | 0.33535245 | 4.60620094 | 4.10E-06 | 4.06E-05 | NR4A1 | chr12 | protein_coding |
| ENSG00000167889.13 | 7.41091766 | 1.79583256 | 0.60715758 | 2.95777011 | 0.00309873 | 0.01309194 | MGAT5B | chr17 | protein_coding |
| ENSG00000126353.3 | 16.9991401 | 1.07214686 | 0.4039815 | 2.65395041 | 0.00795555 | 0.02890837 | CCR7 | chr17 | protein_coding |
| ENSG00000225889.10 | 14.5075198 | 1.41017312 | 0.40955977 | 3.44314366 | 0.00057499 | 0.00311343 | ENSG00000225889 | chr2 | lncRNA |
| ENSG00000287707.1 | 39.322102 | -1.0223628 | 0.25010437 | -4.0877448 | 4.36E-05 | 0.00032937 | ENSG00000287707 | chr3 | lncRNA |
| ENSG00000128872.10 | 23.2220107 | 1.26230875 | 0.36372919 | 3.47046311 | 0.00051956 | 0.00285131 | TMOD2 | chr15 | protein_coding |
| ENSG00000186868.18 | 38.4303868 | 1.48754894 | 0.3348576 | 4.44233291 | 8.90E-06 | 8.09E-05 | MAPT | chr17 | protein_coding |
| ENSG00000159496.15 | 13.0726219 | -1.0927807 | 0.449949 | -2.4286767 | 0.01515404 | 0.04921274 | RGL4 | chr22 | protein_coding |
| ENSG00000042781.14 | 52.3488464 | 1.08878625 | 0.22672578 | 4.80221632 | 1.57E-06 | 1.74E-05 | USH2A | chr1 | protein_coding |
| ENSG00000140678.17 | 84.743081 | 1.07281602 | 0.23075127 | 4.64923124 | 3.33E-06 | 3.37E-05 | ITGAX | chr16 | protein_coding |
| ENSG00000065361.17 | 32.4545415 | 1.58380676 | 0.30159657 | 5.25140836 | 1.51E-07 | 2.13E-06 | ERBB3 | chr12 | protein_coding |
| ENSG00000186439.15 | 11.512989 | 1.60841548 | 0.50397242 | 3.19147518 | 0.00141548 | 0.00671694 | TRDN | chr6 | protein_coding |
| ENSG00000273445.1 | 1292.7781 | 1.05177392 | 0.06480257 | 16.2304356 | 3.07E-59 | 8.11E-56 | ENSG00000273445 | chr2 | lncRNA |
| ENSG00000099617.4 | 5.39261414 | -1.9906084 | 0.78283422 | -2.5428225 | 0.01099611 | 0.03780326 | EFNA2 | chr19 | protein_coding |
| ENSG00000162576.17 | 18.6243411 | 1.15523418 | 0.39020218 | 2.96060411 | 0.00307036 | 0.01298508 | MXRA8 | chr1 | protein_coding |
| ENSG00000166589.13 | 3.4503906 | 4.41672045 | 1.25082744 | 3.53103898 | 0.00041393 | 0.00234413 | CDH16 | chr16 | protein_coding |
| ENSG00000218631.1 | 8.86895245 | 1.60166233 | 0.62555771 | 2.56037503 | 0.01045593 | 0.03639085 | ENSG00000218631 | chr6 | processed_pseudogene |
| ENSG00000291112.1 | 68.0105523 | 1.9379356 | 0.20885909 | 9.27867492 | 1.72E-20 | 2.53E-18 | ENSG00000291112 | chr6 | lncRNA |
| ENSG00000131724.11 | 120.272745 | 1.16560811 | 0.1509398 | 7.72233776 | 1.14E-14 | 6.85E-13 | IL13RA1 | chrX | protein_coding |
| ENSG00000162078.12 | 6.67447627 | 2.063259 | 0.72212647 | 2.85719895 | 0.00427398 | 0.01718709 | ZG16B | chr16 | protein_coding |
| ENSG00000198353.8 | 20.5983682 | 1.41769548 | 0.35504396 | 3.99301395 | 6.52E-05 | 0.000468 | HOXC4 | chr12 | protein_coding |
| ENSG00000180815.15 | 8.31450144 | -1.3982297 | 0.54916662 | -2.5460937 | 0.01089359 | 0.03751192 | MAP3K15 | chrX | protein_coding |
| ENSG00000253633.2 | 20.4932766 | 1.82666448 | 0.41165153 | 4.43740479 | 9.10E-06 | 8.27E-05 | LINC03047 | chr8 | lncRNA |
| ENSG00000108821.14 | 418.053798 | 1.17613248 | 0.12963707 | 9.072501 | 1.16E-19 | 1.53E-17 | COL1A1 | chr17 | protein_coding |
| ENSG00000230303.6 | 9.12657193 | 1.56506558 | 0.54344822 | 2.87987987 | 0.00397827 | 0.01615177 | ENSG00000230303 | chr9 | lncRNA |
| ENSG00000001617.12 | 5.69955447 | 1.98340023 | 0.77535885 | 2.55804167 | 0.01052635 | 0.03657542 | SEMA3F | chr3 | protein_coding |
| ENSG00000146054.18 | 16.9805396 | 1.49527096 | 0.43588285 | 3.43044227 | 0.0006026 | 0.00323223 | TRIM7 | chr5 | protein_coding |
| ENSG00000255337.1 | 4.84512533 | 2.12875375 | 0.77841227 | 2.73473818 | 0.00624299 | 0.02354869 | TMEM123-DT | chr11 | lncRNA |
| ENSG00000259702.3 | 5.34564929 | 2.07640921 | 0.77166626 | 2.69081247 | 0.00712782 | 0.02633787 | ENSG00000259702 | chr15 | lncRNA |
| ENSG00000153291.16 | 20.8004536 | 1.31609056 | 0.40338658 | 3.26260377 | 0.00110394 | 0.00541127 | SLC25A27 | chr6 | protein_coding |
| ENSG00000154102.11 | 5.58451709 | 2.16658328 | 0.75304036 | 2.87711441 | 0.0040133 | 0.01626585 | C16orf74 | chr16 | protein_coding |
| ENSG00000155629.15 | 13.5005369 | 1.25805102 | 0.46170365 | 2.724802 | 0.00643401 | 0.02415699 | PIK3AP1 | chr10 | protein_coding |
| ENSG00000122223.13 | 7.02378821 | 1.84121609 | 0.64177656 | 2.86893633 | 0.00411855 | 0.01663175 | CD244 | chr1 | protein_coding |
| ENSG00000262155.2 | 8.8787368 | 1.63410903 | 0.54789067 | 2.9825458 | 0.00285862 | 0.01218723 | LINC02175 | chr16 | lncRNA |
| ENSG00000259687.4 | 13.392827 | 1.2566666 | 0.46372101 | 2.70996262 | 0.00672908 | 0.02509084 | LINC01220 | chr14 | lncRNA |
| ENSG00000233633.2 | 6.10406346 | 1.91064025 | 0.67074988 | 2.84851373 | 0.0043924 | 0.01759625 | ENSG00000233633 | chr2 | lncRNA |
| ENSG00000125089.18 | 176.468741 | -1.3510921 | 0.16724808 | -8.0783714 | 6.56E-16 | 4.88E-14 | SH3TC1 | chr4 | protein_coding |
| ENSG00000106789.13 | 247.394625 | 1.16635761 | 0.11865418 | 9.82989057 | 8.37E-23 | 1.67E-20 | CORO2A | chr9 | protein_coding |
| ENSG00000000938.13 | 26.3324196 | 1.10979249 | 0.328781 | 3.37547634 | 0.00073688 | 0.00382706 | FGR | chr1 | protein_coding |
| ENSG00000293062.1 | 15.6812566 | 1.00301802 | 0.40347729 | 2.48593427 | 0.01292118 | 0.04309511 | ENSG00000293062 | chr21 | lncRNA |
| ENSG00000290387.1 | 47.8022396 | 1.03916771 | 0.22983793 | 4.52130644 | 6.15E-06 | 5.82E-05 | SORD2P | chr15 | lncRNA |
| ENSG00000101194.18 | 11.7737033 | 1.91624804 | 0.60790636 | 3.15220925 | 0.0016204 | 0.0075402 | SLC17A9 | chr20 | protein_coding |
| ENSG00000272971.1 | 20.8160495 | 2.20677589 | 0.40794063 | 5.40955154 | 6.32E-08 | 9.78E-07 | ENSG00000272971 | chr1 | lncRNA |
| ENSG00000214274.10 | 133.87788 | 1.10879887 | 0.15310461 | 7.24209965 | 4.42E-13 | 1.98E-11 | ANG | chr14 | protein_coding |
| ENSG00000108825.18 | 28.6587441 | 1.34330306 | 0.29297885 | 4.5849831 | 4.54E-06 | 4.46E-05 | PTGES3L-AARSD1 | chr17 | protein_coding |
| ENSG00000293477.1 | 4.27359162 | 2.19011634 | 0.8560554 | 2.55838156 | 0.01051606 | 0.0365457 | CRYZL2P | chr1 | lncRNA |
| ENSG00000021645.20 | 9.90597343 | 1.34981033 | 0.53293125 | 2.53280387 | 0.01131542 | 0.03869311 | NRXN3 | chr14 | protein_coding |
| ENSG00000212123.4 | 376.152872 | -1.0407306 | 0.11341178 | -9.1765652 | 4.45E-20 | 6.22E-18 | PRR22 | chr19 | protein_coding |
| ENSG00000198915.12 | 4.9581025 | 1.95106642 | 0.79224478 | 2.46270657 | 0.01378927 | 0.04545185 | RASGEF1A | chr10 | protein_coding |
| ENSG00000211747.3 | 18.824582 | 1.3687614 | 0.40791642 | 3.35549476 | 0.00079223 | 0.00406257 | TRBV20-1 | chr7 | TR_V_gene |
| ENSG00000166793.13 | 21.4368292 | 2.66627741 | 0.4843237 | 5.50515578 | 3.69E-08 | 6.02E-07 | YPEL4 | chr11 | protein_coding |
| ENSG00000133069.17 | 107.023098 | 1.60349907 | 0.20207081 | 7.93533244 | 2.10E-15 | 1.41E-13 | TMCC2 | chr1 | protein_coding |
| ENSG00000285016.1 | 14.8214763 | 1.18394569 | 0.41845852 | 2.82930235 | 0.00466496 | 0.01848362 | ENSG00000285016 | chr2 | lncRNA |
| ENSG00000110881.12 | 522.491896 | 1.3443861 | 0.19493535 | 6.8965743 | 5.33E-12 | 1.98E-10 | ASIC1 | chr12 | protein_coding |
| ENSG00000253123.4 | 216.783304 | 1.13340078 | 0.12158131 | 9.32216258 | 1.14E-20 | 1.72E-18 | ENSG00000253123 | chr8 | lncRNA |
| ENSG00000166173.11 | 32.0822509 | 1.01006884 | 0.27510255 | 3.67160844 | 0.00024103 | 0.00147753 | LARP6 | chr15 | protein_coding |
| ENSG00000109705.8 | 15.9944893 | 1.46685251 | 0.44333435 | 3.3086823 | 0.00093736 | 0.00468947 | NKX3-2 | chr4 | protein_coding |
| ENSG00000291036.1 | 52.6623675 | 1.3882017 | 0.29073672 | 4.77477257 | 1.80E-06 | 1.97E-05 | ENSG00000291036 | chr6 | lncRNA |
| ENSG00000170786.13 | 28.6338192 | 1.62040116 | 0.33598115 | 4.8228931 | 1.41E-06 | 1.58E-05 | SDR16C5 | chr8 | protein_coding |
| ENSG00000007350.18 | 61.7534763 | 1.62400741 | 0.2147097 | 7.56373579 | 3.92E-14 | 2.16E-12 | TKTL1 | chrX | protein_coding |
| ENSG00000152527.14 | 61.7539021 | 1.01150854 | 0.21367598 | 4.73384299 | 2.20E-06 | 2.34E-05 | PLEKHH2 | chr2 | protein_coding |
| ENSG00000226425.1 | 8.26740404 | 1.68052758 | 0.61651499 | 2.72585032 | 0.00641361 | 0.02410612 | CYP26C1-DT | chr10 | lncRNA |
| ENSG00000166920.13 | 320.693862 | 2.25610026 | 0.26030697 | 8.66707589 | 4.43E-18 | 4.42E-16 | C15orf48 | chr15 | protein_coding |
| ENSG00000166046.11 | 18.7194492 | 2.36223317 | 0.44314667 | 5.33058995 | 9.79E-08 | 1.45E-06 | TCP11L2 | chr12 | protein_coding |
| ENSG00000198945.8 | 50.6358488 | 1.51799626 | 0.24045638 | 6.3129798 | 2.74E-10 | 7.10E-09 | L3MBTL3 | chr6 | protein_coding |
| ENSG00000287743.1 | 22.1432465 | 1.01422398 | 0.34499107 | 2.93985575 | 0.00328365 | 0.01376304 | ENSG00000287743 | chr1 | lncRNA |
| ENSG00000189238.6 | 17.1249437 | -1.1830121 | 0.38370784 | -3.0831064 | 0.00204852 | 0.00919004 | LINC00943 | chr12 | lncRNA |
| ENSG00000092200.13 | 28.3483896 | 1.30061689 | 0.300632 | 4.32627567 | 1.52E-05 | 0.00013063 | RPGRIP1 | chr14 | protein_coding |
| ENSG00000185818.8 | 4.0556916 | 2.08173855 | 0.85803023 | 2.42618324 | 0.01525856 | 0.0495065 | NAT8L | chr4 | protein_coding |
| ENSG00000117971.12 | 28.6829868 | 1.12891989 | 0.29480012 | 3.82944181 | 0.00012843 | 0.00085429 | CHRNB4 | chr15 | protein_coding |
| ENSG00000268055.2 | 4.50669333 | 2.27046883 | 0.84673351 | 2.68144439 | 0.00733051 | 0.02698077 | ENSG00000268055 | chr19 | lncRNA |
| ENSG00000139973.18 | 54.9167656 | 1.31144543 | 0.21907359 | 5.98632379 | 2.15E-09 | 4.53E-08 | SYT16 | chr14 | protein_coding |
| ENSG00000164089.9 | 141.193312 | 1.02846984 | 0.13878879 | 7.41032331 | 1.26E-13 | 6.21E-12 | ETNPPL | chr4 | protein_coding |
| ENSG00000256948.1 | 52.1195343 | 1.09041038 | 0.22392547 | 4.86952366 | 1.12E-06 | 1.28E-05 | IQSEC3-AS2 | chr12 | lncRNA |
| ENSG00000174938.15 | 151.996354 | 1.09271211 | 0.20078901 | 5.44209124 | 5.27E-08 | 8.28E-07 | SEZ6L2 | chr16 | protein_coding |
| ENSG00000235641.5 | 4.90420991 | 3.55308712 | 1.08255394 | 3.28213402 | 0.00103025 | 0.00509978 | LINC00484 | chr9 | lncRNA |
| ENSG00000153563.17 | 21.052018 | 1.41151244 | 0.36721849 | 3.8437946 | 0.00012115 | 0.00080989 | CD8A | chr2 | protein_coding |
| ENSG00000164683.19 | 26.3837149 | 1.11468632 | 0.32014538 | 3.48181298 | 0.00049803 | 0.00275897 | HEY1 | chr8 | protein_coding |
| ENSG00000206052.11 | 24.8320156 | 1.48501817 | 0.33214809 | 4.47095194 | 7.79E-06 | 7.19E-05 | DOK6 | chr18 | protein_coding |
| ENSG00000213626.13 | 35.1996399 | 1.28665881 | 0.28642042 | 4.49220345 | 7.05E-06 | 6.57E-05 | LBH | chr2 | protein_coding |
| ENSG00000213062.5 | 45.5979738 | 1.05576237 | 0.27742781 | 3.80553911 | 0.0001415 | 0.00092916 | ENSG00000213062 | chr1 | lncRNA |
| ENSG00000268199.2 | 16.8659999 | -1.0038367 | 0.40880043 | -2.4555668 | 0.01406626 | 0.04619896 | ENSG00000268199 | chr19 | lncRNA |
| ENSG00000247728.2 | 14.7738203 | 1.0793867 | 0.4039495 | 2.67208328 | 0.00753819 | 0.02762478 | ARHGAP11B-DT | chr15 | lncRNA |
| ENSG00000107796.13 | 147.110193 | 1.10796319 | 0.15049425 | 7.36216287 | 1.81E-13 | 8.68E-12 | ACTA2 | chr10 | protein_coding |
| ENSG00000176907.5 | 11.2706294 | 2.07079828 | 0.50869598 | 4.07079744 | 4.69E-05 | 0.00035126 | TCIM | chr8 | protein_coding |
| ENSG00000106123.12 | 61.0613374 | 1.04546029 | 0.22430914 | 4.66080118 | 3.15E-06 | 3.21E-05 | EPHB6 | chr7 | protein_coding |
| ENSG00000231292.6 | 4.60406778 | 2.32187134 | 0.85205574 | 2.72502281 | 0.0064297 | 0.02414513 | IGKV1OR2-108 | chr2 | IG_V_gene |
| ENSG00000118898.16 | 73.9168933 | 1.80722953 | 0.24496871 | 7.37738914 | 1.61E-13 | 7.85E-12 | PPL | chr16 | protein_coding |
| ENSG00000164707.16 | 24.1907066 | 1.09683357 | 0.32745635 | 3.34955658 | 0.00080941 | 0.00413559 | SLC13A4 | chr7 | protein_coding |
| ENSG00000237412.7 | 4.75455474 | 4.8989547 | 1.20454242 | 4.06706696 | 4.76E-05 | 0.00035567 | PRSS56 | chr2 | protein_coding |
| ENSG00000230023.2 | 7.82125558 | 1.88348561 | 0.59286603 | 3.17691605 | 0.0014885 | 0.0070224 | LINC02800 | chr1 | lncRNA |
| ENSG00000291158.1 | 40.1352166 | 1.14624296 | 0.26580367 | 4.312367 | 1.62E-05 | 0.00013794 | LINC00869 | chr1 | lncRNA |
| ENSG00000258761.1 | 15.2525967 | -1.1269032 | 0.42198423 | -2.6704866 | 0.00757414 | 0.02772282 | ENSG00000258761 | chr15 | lncRNA |
| ENSG00000143994.14 | 115.878371 | 1.16240903 | 0.15879064 | 7.32038754 | 2.47E-13 | 1.15E-11 | ABHD1 | chr2 | protein_coding |
| ENSG00000259577.2 | 69.8574758 | 1.47310405 | 0.19846394 | 7.42252738 | 1.15E-13 | 5.73E-12 | CERNA1 | chr15 | lncRNA |
| ENSG00000189325.7 | 9.84419012 | 1.39607666 | 0.52272474 | 2.67076827 | 0.00756779 | 0.02770437 | BNIP5 | chr6 | protein_coding |
| ENSG00000064270.13 | 15.0591611 | 1.81444574 | 0.4421906 | 4.10331146 | 4.07E-05 | 0.00030974 | ATP2C2 | chr16 | protein_coding |
| ENSG00000115255.12 | 117.255978 | 1.23178835 | 0.38879605 | 3.16821213 | 0.0015338 | 0.0072071 | REEP6 | chr19 | protein_coding |
| ENSG00000124466.9 | 67.7496709 | 1.2200951 | 0.24555539 | 4.96871653 | 6.74E-07 | 8.25E-06 | LYPD3 | chr19 | protein_coding |
| ENSG00000137474.23 | 8.00907403 | 1.93305288 | 0.58410737 | 3.30941359 | 0.00093492 | 0.00467835 | MYO7A | chr11 | protein_coding |
| ENSG00000171992.13 | 198.368055 | 1.04721712 | 0.11789013 | 8.8829927 | 6.51E-19 | 7.63E-17 | SYNPO | chr5 | protein_coding |
| ENSG00000141096.6 | 11.1058814 | 1.54639233 | 0.51654602 | 2.99371646 | 0.00275602 | 0.01181663 | DPEP3 | chr16 | protein_coding |
| ENSG00000260121.1 | 22.3212462 | -1.1606472 | 0.34931306 | -3.3226562 | 0.00089165 | 0.00449057 | ENSG00000260121 | chr16 | lncRNA |
| ENSG00000152642.11 | 74.9771759 | 1.22464405 | 0.20203162 | 6.06164548 | 1.35E-09 | 2.96E-08 | GPD1L | chr3 | protein_coding |
| ENSG00000280649.2 | 27.376526 | 1.41038491 | 0.33756299 | 4.17813847 | 2.94E-05 | 0.00023336 | ENSG00000280649 | chr1 | TEC |
| ENSG00000071282.12 | 2.79664131 | 3.29594153 | 1.29639677 | 2.5423864 | 0.01100984 | 0.03783814 | LMCD1 | chr3 | protein_coding |
| ENSG00000176402.6 | 8.60977455 | 1.80792905 | 0.57036785 | 3.16975976 | 0.00152565 | 0.00717361 | GJC3 | chr7 | protein_coding |
| ENSG00000143036.17 | 6.0207908 | 1.87031965 | 0.74340076 | 2.51589688 | 0.01187299 | 0.04019652 | SLC44A3 | chr1 | protein_coding |
| ENSG00000187166.1 | 3.63438892 | 3.70595593 | 1.21545671 | 3.04902338 | 0.00229587 | 0.01013176 | H1-7 | chr12 | protein_coding |
| ENSG00000182676.5 | 3.93374462 | -2.1695829 | 0.86036452 | -2.521702 | 0.01167886 | 0.03969797 | PPP1R27 | chr17 | protein_coding |
| ENSG00000273274.2 | 22.5256755 | 1.0504872 | 0.34096577 | 3.08091685 | 0.00206364 | 0.00925003 | ZBTB8B | chr1 | protein_coding |
| ENSG00000163449.11 | 32.0842306 | 1.3847891 | 0.28522747 | 4.85503414 | 1.20E-06 | 1.37E-05 | TMEM169 | chr2 | protein_coding |
| ENSG00000273284.1 | 28.1207381 | 1.00880217 | 0.32801922 | 3.0754362 | 0.00210195 | 0.00940178 | ENSG00000273284 | chr18 | lncRNA |
| ENSG00000184261.5 | 42.0223156 | -1.1466508 | 0.24574281 | -4.6660603 | 3.07E-06 | 3.14E-05 | KCNK12 | chr2 | protein_coding |
| ENSG00000117266.16 | 40.7369236 | 1.32681945 | 0.26784855 | 4.95361818 | 7.28E-07 | 8.84E-06 | CDK18 | chr1 | protein_coding |
| ENSG00000246863.2 | 15.2458179 | 1.16621586 | 0.44865601 | 2.59935417 | 0.00933994 | 0.03309024 | GPR176-DT | chr15 | lncRNA |
| ENSG00000172538.7 | 11.9430926 | 1.23685226 | 0.49612645 | 2.4930182 | 0.01266624 | 0.04237869 | FAM170B | chr10 | protein_coding |
| ENSG00000170004.19 | 17.7959343 | 1.30263862 | 0.41214072 | 3.16066466 | 0.0015741 | 0.00736858 | CHD3 | chr17 | protein_coding |
| ENSG00000165029.17 | 44.3742891 | 1.01907371 | 0.2561188 | 3.97891021 | 6.92E-05 | 0.00049246 | ABCA1 | chr9 | protein_coding |
| ENSG00000152953.13 | 16.6697948 | 1.01165779 | 0.39793606 | 2.54226217 | 0.01101375 | 0.03784543 | STK32B | chr4 | protein_coding |
| ENSG00000259803.8 | 24.1581214 | 1.80524207 | 0.33932324 | 5.32012507 | 1.04E-07 | 1.52E-06 | SLC22A31 | chr16 | protein_coding |
| ENSG00000149177.15 | 15.3230475 | 1.07478011 | 0.44220775 | 2.43048684 | 0.01507855 | 0.0490053 | PTPRJ | chr11 | protein_coding |
| ENSG00000238121.7 | 10.8352549 | 1.41804579 | 0.52651276 | 2.69327904 | 0.0070753 | 0.02619674 | LINC00426 | chr13 | lncRNA |
| ENSG00000143126.8 | 117.805302 | 1.02035301 | 0.16005885 | 6.37486148 | 1.83E-10 | 4.94E-09 | CELSR2 | chr1 | protein_coding |
| ENSG00000105707.15 | 4.5872504 | 3.46426404 | 1.01642281 | 3.40829033 | 0.00065371 | 0.00345548 | HPN | chr19 | protein_coding |
| ENSG00000167107.13 | 387.403266 | 1.23091468 | 0.10576687 | 11.6379986 | 2.64E-31 | 1.21E-28 | ACSF2 | chr17 | protein_coding |
| ENSG00000186994.12 | 55.4485155 | 1.75019911 | 0.25746329 | 6.79785877 | 1.06E-11 | 3.71E-10 | KANK3 | chr19 | protein_coding |
| ENSG00000204060.8 | 17.4785474 | 1.76904501 | 0.42002597 | 4.2117515 | 2.53E-05 | 0.00020434 | FOXO6 | chr1 | protein_coding |
| ENSG00000167861.16 | 14.4757665 | 1.96980797 | 0.47022134 | 4.18910803 | 2.80E-05 | 0.00022337 | HID1 | chr17 | protein_coding |
| ENSG00000289141.2 | 23.5330482 | 1.04141659 | 0.33630201 | 3.09667075 | 0.00195707 | 0.00884558 | ENSG00000289141 | chr1 | lncRNA |
| ENSG00000105251.11 | 9.76653905 | 1.49047072 | 0.53440386 | 2.78903434 | 0.00528655 | 0.02050892 | SHD | chr19 | protein_coding |
| ENSG00000189143.10 | 13.9163383 | 1.44093793 | 0.48904564 | 2.94642833 | 0.00321467 | 0.01351147 | CLDN4 | chr7 | protein_coding |
| ENSG00000153982.11 | 170.470851 | 1.20138987 | 0.14741573 | 8.14967232 | 3.65E-16 | 2.81E-14 | GDPD1 | chr17 | protein_coding |
| ENSG00000049089.15 | 71.7950658 | 1.67898976 | 0.19973597 | 8.40604598 | 4.24E-17 | 3.73E-15 | COL9A2 | chr1 | protein_coding |
| ENSG00000142583.18 | 3.89619013 | 2.32402978 | 0.95725494 | 2.4278065 | 0.01519045 | 0.04930822 | SLC2A5 | chr1 | protein_coding |
| ENSG00000173702.7 | 13.2677123 | 1.81368013 | 0.51684178 | 3.50915926 | 0.00044953 | 0.00252376 | MUC13 | chr3 | protein_coding |
| ENSG00000145198.15 | 106.308392 | 1.30534993 | 0.17842155 | 7.31610031 | 2.55E-13 | 1.18E-11 | VWA5B2 | chr3 | protein_coding |
| ENSG00000187833.8 | 69.8767852 | 1.62937507 | 0.21430661 | 7.60300887 | 2.89E-14 | 1.64E-12 | C2orf78 | chr2 | protein_coding |
| ENSG00000257660.5 | 11.9215769 | 1.20198667 | 0.46529139 | 2.58329877 | 0.00978605 | 0.03443386 | ADCY6-DT | chr12 | lncRNA |
| ENSG00000170293.9 | 9.82826436 | 1.6116239 | 0.51124848 | 3.15232995 | 0.00161973 | 0.00753875 | CMTM8 | chr3 | protein_coding |
| ENSG00000289332.2 | 12.521514 | 1.93522633 | 0.48826625 | 3.96346528 | 7.39E-05 | 0.00052034 | ENSG00000289332 | chr13 | lncRNA |
| ENSG00000104783.15 | 25.3153149 | 1.61276784 | 0.44797378 | 3.60013895 | 0.00031805 | 0.00187297 | KCNN4 | chr19 | protein_coding |
| ENSG00000288045.1 | 22.8049536 | 1.10300096 | 0.34776203 | 3.17171191 | 0.00151543 | 0.00713033 | ENSG00000288045 | chr14 | lncRNA |
| ENSG00000272970.5 | 7.71097043 | 1.45656807 | 0.57108194 | 2.55054132 | 0.01075558 | 0.03715787 | ENSG00000272970 | chr3 | lncRNA |
| ENSG00000254475.1 | 5.74669177 | -2.3411734 | 0.75860416 | -3.0861595 | 0.0020276 | 0.0091172 | OR2AT1P | chr11 | unprocessed_pseudogene |
| ENSG00000258512.2 | 5.38302429 | 1.87465334 | 0.7705817 | 2.43277687 | 0.01498353 | 0.0487415 | LINC00239 | chr14 | lncRNA |
| ENSG00000185432.12 | 368.122274 | 1.29338927 | 0.09915596 | 13.0439889 | 6.88E-39 | 6.05E-36 | TMT1A | chr12 | protein_coding |
| ENSG00000198932.13 | 21.6125081 | 1.92845273 | 0.38206405 | 5.04745925 | 4.48E-07 | 5.70E-06 | GPRASP1 | chrX | protein_coding |
| ENSG00000184640.20 | 60.8214268 | 1.23061993 | 0.25606887 | 4.80581626 | 1.54E-06 | 1.71E-05 | SEPTIN9 | chr17 | protein_coding |
| ENSG00000230701.2 | 10.5433971 | -1.8627216 | 0.53546666 | -3.4786882 | 0.00050387 | 0.00278549 | FBXW4P1 | chr22 | processed_pseudogene |
| ENSG00000250669.1 | 9.30602416 | 1.58824483 | 0.52430498 | 3.02923849 | 0.00245171 | 0.01071203 | ENSG00000250669 | chr5 | processed_pseudogene |
| ENSG00000227963.2 | 18.3088083 | -1.1288268 | 0.36438425 | -3.0979022 | 0.00194896 | 0.00882023 | RBM15-AS1 | chr1 | lncRNA |
| ENSG00000204682.9 | 11.3549702 | 2.04605337 | 0.56997218 | 3.58974253 | 0.000331 | 0.00194086 | MIR1915HG | chr10 | lncRNA |
| ENSG00000168899.5 | 67.3812924 | 1.02849356 | 0.20109968 | 5.11434714 | 3.15E-07 | 4.16E-06 | VAMP5 | chr2 | protein_coding |
| ENSG00000105538.10 | 43.3364635 | 1.20317427 | 0.25001847 | 4.81234146 | 1.49E-06 | 1.66E-05 | RASIP1 | chr19 | protein_coding |
| ENSG00000258818.4 | 144.891685 | 1.21992554 | 0.15687389 | 7.77647275 | 7.46E-15 | 4.56E-13 | RNASE4 | chr14 | protein_coding |
| ENSG00000178947.9 | 6.75993393 | 1.59885558 | 0.65339191 | 2.44700855 | 0.01440474 | 0.04708359 | SMIM10L2A | chrX | protein_coding |
| ENSG00000185519.9 | 16.337288 | 1.22840622 | 0.39188117 | 3.13463957 | 0.00172065 | 0.00793814 | FAM131C | chr1 | protein_coding |
| ENSG00000138435.16 | 87.559199 | 1.25225762 | 0.20172195 | 6.20784011 | 5.37E-10 | 1.31E-08 | CHRNA1 | chr2 | protein_coding |
| ENSG00000184588.18 | 74.3084722 | 1.82075707 | 0.21511358 | 8.46416607 | 2.58E-17 | 2.33E-15 | PDE4B | chr1 | protein_coding |
| ENSG00000178033.6 | 61.759897 | 1.18507564 | 0.21104281 | 5.61533304 | 1.96E-08 | 3.39E-07 | CALHM5 | chr6 | protein_coding |
| ENSG00000099365.11 | 17.500338 | 1.66440191 | 0.40919437 | 4.06750929 | 4.75E-05 | 0.00035542 | STX1B | chr16 | protein_coding |
| ENSG00000239282.8 | 13.0032707 | 1.58080467 | 0.50292317 | 3.14323291 | 0.00167093 | 0.0077412 | CASTOR1 | chr22 | protein_coding |
| ENSG00000211829.9 | 12.7989976 | 1.08112066 | 0.43824422 | 2.46693649 | 0.01362745 | 0.04505917 | TRDC | chr14 | TR_C_gene |
| ENSG00000287207.1 | 15.4549832 | 1.66536577 | 0.46053508 | 3.61615403 | 0.00029901 | 0.00177275 | ENSG00000287207 | chr3 | lncRNA |
| ENSG00000228288.9 | 12.1570215 | 1.33807456 | 0.48068333 | 2.78369246 | 0.0053744 | 0.020781 | PCAT6 | chr1 | lncRNA |
| ENSG00000167772.12 | 6.71079053 | 3.60760233 | 0.87270554 | 4.13381392 | 3.57E-05 | 0.00027622 | ANGPTL4 | chr19 | protein_coding |
| ENSG00000253868.4 | 4.30403775 | 2.16601149 | 0.86004891 | 2.51847478 | 0.01178643 | 0.03998637 | FER1L6-AS2 | chr8 | lncRNA |
| ENSG00000127241.18 | 24.1766671 | 1.6795104 | 0.41999188 | 3.99891161 | 6.36E-05 | 0.00045773 | MASP1 | chr3 | protein_coding |
| ENSG00000162148.11 | 31.455665 | 1.10945467 | 0.30534628 | 3.63343108 | 0.00027968 | 0.00167695 | SAXO4 | chr11 | protein_coding |
| ENSG00000291234.1 | 15.9637111 | 1.30303297 | 0.41608494 | 3.13165138 | 0.00173826 | 0.00800396 | ENSG00000291234 | chr1 | lncRNA |
| ENSG00000237949.3 | 7.48491997 | 2.62434028 | 0.72347222 | 3.62742371 | 0.00028626 | 0.00170819 | LINC00844 | chr10 | lncRNA |
| ENSG00000289602.1 | 10.9632227 | 1.43556801 | 0.48632341 | 2.95187931 | 0.00315846 | 0.01332031 | ENSG00000289602 | chr1 | lncRNA |
| ENSG00000174804.4 | 82.3037298 | 1.0169352 | 0.23349706 | 4.35523768 | 1.33E-05 | 0.00011615 | FZD4 | chr11 | protein_coding |
| ENSG00000135097.7 | 6.89545062 | 2.52717772 | 0.77519347 | 3.26006063 | 0.00111388 | 0.00545369 | MSI1 | chr12 | protein_coding |
| ENSG00000135362.14 | 14.379108 | 1.11462412 | 0.45116134 | 2.47056656 | 0.01348992 | 0.04468841 | PRR5L | chr11 | protein_coding |
| ENSG00000165795.25 | 66.0140187 | 1.15470873 | 0.20646029 | 5.59288523 | 2.23E-08 | 3.80E-07 | NDRG2 | chr14 | protein_coding |
| ENSG00000161682.15 | 47.2907923 | 1.00280645 | 0.23491247 | 4.26885147 | 1.96E-05 | 0.00016376 | FAM171A2 | chr17 | protein_coding |
| ENSG00000286452.1 | 6.11954395 | 1.89206645 | 0.72241303 | 2.61909237 | 0.00881641 | 0.03149441 | ENSG00000286452 | chr6 | lncRNA |
| ENSG00000187398.12 | 5.74137335 | 1.98161211 | 0.71117366 | 2.78639696 | 0.00532976 | 0.0206462 | LUZP2 | chr11 | protein_coding |
| ENSG00000148600.15 | 6.06600142 | -2.1806454 | 0.88161089 | -2.4734783 | 0.01338049 | 0.04437464 | CDHR1 | chr10 | protein_coding |
| ENSG00000284747.1 | 30.9557887 | -1.1441006 | 0.30166233 | -3.7926531 | 0.00014905 | 0.0009739 | ENSG00000284747 | chr1 | lncRNA |
| ENSG00000278964.1 | 8.55549841 | 1.44431078 | 0.56502762 | 2.55617732 | 0.01058292 | 0.03672363 | ENSG00000278964 | chr17 | TEC |
| ENSG00000244255.5 | 45.1243599 | 1.27423777 | 0.24455003 | 5.21054015 | 1.88E-07 | 2.60E-06 | ENSG00000244255 | chr6 | protein_coding |
| ENSG00000167261.14 | 21.9885249 | 2.03082664 | 0.44919006 | 4.52108548 | 6.15E-06 | 5.83E-05 | DPEP2 | chr16 | protein_coding |
| ENSG00000226310.2 | 8.34542833 | 1.39012444 | 0.55172568 | 2.51959352 | 0.01174904 | 0.03988516 | ENSG00000226310 | chrX | lncRNA |
| ENSG00000100196.11 | 13.55414 | 2.01267155 | 0.46698149 | 4.30996002 | 1.63E-05 | 0.00013934 | KDELR3 | chr22 | protein_coding |
| ENSG00000168060.16 | 10.6268356 | 1.20852383 | 0.49632128 | 2.43496277 | 0.01489332 | 0.0484854 | NAALADL1 | chr11 | protein_coding |
| ENSG00000205517.13 | 121.788491 | 1.99392883 | 0.16850189 | 11.8332725 | 2.63E-32 | 1.32E-29 | RGL3 | chr19 | protein_coding |
| ENSG00000129170.10 | 24.3051045 | 1.73258637 | 0.39635709 | 4.37127631 | 1.24E-05 | 0.00010879 | CSRP3 | chr11 | protein_coding |
| ENSG00000107551.21 | 69.5441271 | 1.68199567 | 0.20769596 | 8.0983553 | 5.57E-16 | 4.19E-14 | RASSF4 | chr10 | protein_coding |
| ENSG00000293413.1 | 279.269607 | 1.03417602 | 0.11491865 | 8.99920105 | 2.27E-19 | 2.89E-17 | ENSG00000293413 | chr20 | lncRNA |
| ENSG00000113758.14 | 69.1492072 | 1.504137 | 0.23635758 | 6.36381963 | 1.97E-10 | 5.25E-09 | DBN1 | chr5 | protein_coding |
| ENSG00000139194.8 | 11.3936814 | 2.09010485 | 0.56191594 | 3.71960413 | 0.00019954 | 0.00125824 | RBP5 | chr12 | protein_coding |
| ENSG00000126583.12 | 15.327099 | 1.40290524 | 0.44025524 | 3.1865725 | 0.00143969 | 0.0068211 | PRKCG | chr19 | protein_coding |
| ENSG00000196950.14 | 104.515584 | 1.25261011 | 0.17614556 | 7.11122151 | 1.15E-12 | 4.81E-11 | SLC39A10 | chr2 | protein_coding |
| ENSG00000182791.5 | 15.2371833 | 1.2604145 | 0.42574406 | 2.96049814 | 0.00307142 | 0.01298694 | CCDC87 | chr11 | protein_coding |
| ENSG00000173762.8 | 7.48232862 | 2.44884334 | 0.69580524 | 3.51943793 | 0.00043246 | 0.00243926 | CD7 | chr17 | protein_coding |
| ENSG00000286403.1 | 13.4457702 | 1.75228505 | 0.47713009 | 3.67255196 | 0.00024014 | 0.00147358 | ENSG00000286403 | chr5 | lncRNA |
| ENSG00000213190.4 | 178.856708 | 1.02839913 | 0.12680016 | 8.1103931 | 5.05E-16 | 3.82E-14 | MLLT11 | chr1 | protein_coding |
| ENSG00000188026.13 | 49.942032 | 1.36298354 | 0.24119461 | 5.65097006 | 1.60E-08 | 2.79E-07 | RILPL1 | chr12 | protein_coding |
| ENSG00000231050.3 | 15.6162957 | 1.30069304 | 0.42234551 | 3.07968953 | 0.00207216 | 0.00928625 | GNB1-DT | chr1 | lncRNA |
| ENSG00000231226.1 | 7.46133753 | 2.66235017 | 0.69540631 | 3.82848146 | 0.00012894 | 0.00085736 | TRIM31-AS1 | chr6 | lncRNA |
| ENSG00000269514.3 | 15.4149779 | 1.05666899 | 0.40034097 | 2.63942258 | 0.00830474 | 0.02996064 | ENSG00000269514 | chr12 | lncRNA |
| ENSG00000141540.11 | 7.10192968 | 1.55330474 | 0.61664463 | 2.51896255 | 0.01177012 | 0.03993743 | TTYH2 | chr17 | protein_coding |
| ENSG00000160307.10 | 2.5730032 | 4.76221633 | 1.40275102 | 3.39491204 | 0.00068651 | 0.00360356 | S100B | chr21 | protein_coding |
| ENSG00000128218.8 | 22.4057504 | 1.48410826 | 0.34618836 | 4.28699639 | 1.81E-05 | 0.00015257 | VPREB3 | chr22 | protein_coding |
| ENSG00000165171.11 | 30.9448127 | 1.46141028 | 0.29386735 | 4.97302703 | 6.59E-07 | 8.09E-06 | METTL27 | chr7 | protein_coding |
| ENSG00000287862.1 | 26.2298972 | 1.09572058 | 0.3260015 | 3.36109065 | 0.00077635 | 0.00399863 | ENSG00000287862 | chr5 | lncRNA |
| ENSG00000136999.5 | 7.93998515 | 2.03917146 | 0.62520099 | 3.26162545 | 0.00110775 | 0.00542746 | CCN3 | chr8 | protein_coding |
| ENSG00000229891.4 | 25.9744226 | 1.20588158 | 0.33559354 | 3.59328013 | 0.00032654 | 0.00191711 | LINC01315 | chr22 | lncRNA |
| ENSG00000147443.13 | 23.1239888 | 1.4927455 | 0.35469531 | 4.20852901 | 2.57E-05 | 0.00020689 | DOK2 | chr8 | protein_coding |
| ENSG00000176723.10 | 18.0827967 | 1.32592253 | 0.39272067 | 3.37624835 | 0.00073482 | 0.00381822 | ZNF843 | chr16 | protein_coding |
| ENSG00000233999.3 | 222.358728 | 1.40368576 | 0.11661822 | 12.0365908 | 2.28E-33 | 1.38E-30 | IGKV3OR2-268 | chr2 | IG_V_gene |
| ENSG00000064195.7 | 10.8244635 | -1.247287 | 0.51506794 | -2.421597 | 0.01545247 | 0.04998202 | DLX3 | chr17 | protein_coding |
| ENSG00000081985.14 | 12.5375421 | 1.25222014 | 0.4468019 | 2.80262939 | 0.00506879 | 0.01981341 | IL12RB2 | chr1 | protein_coding |
| ENSG00000273456.2 | 16.4902368 | 1.2268195 | 0.43163008 | 2.84229379 | 0.00447902 | 0.01786193 | ENSG00000273456 | chr2 | lncRNA |
| ENSG00000088280.21 | 230.388914 | 1.24804503 | 0.1337806 | 9.32904309 | 1.07E-20 | 1.62E-18 | ASAP3 | chr1 | protein_coding |
| ENSG00000223552.3 | 22.6397934 | 1.19154429 | 0.34904279 | 3.41374846 | 0.00064076 | 0.00340404 | CCR5AS | chr3 | lncRNA |
| ENSG00000171017.11 | 9.51466587 | 1.6375436 | 0.59444414 | 2.75474766 | 0.00587374 | 0.02240405 | LRRC8E | chr19 | protein_coding |
| ENSG00000217716.3 | 36.2476134 | -1.1138453 | 0.40110134 | -2.7769673 | 0.00548687 | 0.02113856 | RPS10P3 | chr9 | processed_pseudogene |
| ENSG00000107281.10 | 16.4128121 | 1.04226712 | 0.41519052 | 2.51033457 | 0.01206168 | 0.04073036 | NPDC1 | chr9 | protein_coding |
| ENSG00000105971.15 | 148.004366 | 1.17010971 | 0.17804527 | 6.57197846 | 4.97E-11 | 1.52E-09 | CAV2 | chr7 | protein_coding |
| ENSG00000291212.1 | 44.3059656 | 1.19405005 | 0.25170048 | 4.74393233 | 2.10E-06 | 2.24E-05 | ENSG00000291212 | chr15 | lncRNA |
| ENSG00000253526.1 | 3.43250973 | 2.97516749 | 1.07101606 | 2.77789252 | 0.00547127 | 0.02108992 | ENSG00000253526 | chr8 | lncRNA |
| ENSG00000167123.20 | 433.907672 | 1.00900112 | 0.1122009 | 8.99280797 | 2.41E-19 | 3.04E-17 | CERCAM | chr9 | protein_coding |
| ENSG00000248905.10 | 66.0355251 | 1.32356686 | 0.24779119 | 5.34146052 | 9.22E-08 | 1.37E-06 | FMN1 | chr15 | protein_coding |
| ENSG00000206337.12 | 196.086421 | 1.04532621 | 0.12965262 | 8.06251485 | 7.47E-16 | 5.46E-14 | HCP5 | chr6 | lncRNA |
| ENSG00000187908.20 | 73.1625351 | 3.18027554 | 0.27264418 | 11.6645641 | 1.93E-31 | 9.07E-29 | DMBT1 | chr10 | protein_coding |
| ENSG00000285201.1 | 4.41003722 | 2.261788 | 0.87408767 | 2.58759857 | 0.00966475 | 0.0340752 | ENSG00000285201 | chr1 | lncRNA |
| ENSG00000108387.16 | 21.3887701 | 1.52861949 | 0.37665303 | 4.05842876 | 4.94E-05 | 0.00036674 | SEPTIN4 | chr17 | protein_coding |
| ENSG00000010310.9 | 78.3066317 | 2.39278173 | 0.2789562 | 8.57762534 | 9.69E-18 | 9.17E-16 | GIPR | chr19 | protein_coding |
| ENSG00000273771.1 | 16.4488177 | 1.51677704 | 0.44457023 | 3.41178271 | 0.0006454 | 0.00342351 | ENSG00000273771 | chr15 | lncRNA |
| ENSG00000104055.17 | 7.24627668 | 1.87065884 | 0.65559133 | 2.85339166 | 0.00432553 | 0.01736792 | TGM5 | chr15 | protein_coding |
| ENSG00000130307.12 | 15.1932853 | 1.08696546 | 0.44374458 | 2.44952953 | 0.0143043 | 0.04682052 | USHBP1 | chr19 | protein_coding |
| ENSG00000136425.14 | 13.1995119 | 1.50092001 | 0.49207058 | 3.05021283 | 0.00228679 | 0.01010227 | CIB2 | chr15 | protein_coding |
| ENSG00000288931.1 | 23.448761 | 1.4155619 | 0.34391453 | 4.11602815 | 3.85E-05 | 0.00029527 | ENSG00000288931 | chr14 | lncRNA |
| ENSG00000076826.10 | 77.7777614 | 1.233604 | 0.18763533 | 6.57447614 | 4.88E-11 | 1.50E-09 | CAMSAP3 | chr19 | protein_coding |
| ENSG00000147647.13 | 101.997147 | -1.0611358 | 0.15596043 | -6.8038784 | 1.02E-11 | 3.57E-10 | DPYS | chr8 | protein_coding |
| ENSG00000090539.16 | 197.472227 | 1.1534094 | 0.15226236 | 7.57514462 | 3.59E-14 | 2.00E-12 | CHRD | chr3 | protein_coding |
| ENSG00000290592.1 | 4.64694351 | 2.99829414 | 0.98582126 | 3.0414176 | 0.00235467 | 0.01036504 | ENSG00000290592 | chr1 | lncRNA |
| ENSG00000159337.7 | 7.57831742 | 2.90370814 | 0.72307618 | 4.01577072 | 5.93E-05 | 0.00043042 | PLA2G4D | chr15 | protein_coding |
| ENSG00000129159.9 | 19.7105444 | 1.18331377 | 0.39227132 | 3.01656971 | 0.00255652 | 0.01110106 | KCNC1 | chr11 | protein_coding |
| ENSG00000009724.18 | 55.3794807 | -1.3656364 | 0.21766299 | -6.2740863 | 3.52E-10 | 8.92E-09 | MASP2 | chr1 | protein_coding |
| ENSG00000066185.13 | 39.9514618 | 1.17084707 | 0.25326069 | 4.62309036 | 3.78E-06 | 3.77E-05 | ZMYND12 | chr1 | protein_coding |
| ENSG00000102409.10 | 160.52217 | 1.17568873 | 0.13883447 | 8.4682769 | 2.49E-17 | 2.26E-15 | BEX4 | chrX | protein_coding |
| ENSG00000006377.11 | 30.8391667 | 1.03885193 | 0.29857988 | 3.47930991 | 0.00050271 | 0.00278121 | DLX6 | chr7 | protein_coding |
| ENSG00000287926.1 | 8.28015717 | -1.4071144 | 0.56303358 | -2.499166 | 0.0124486 | 0.04178296 | ENSG00000287926 | chr15 | lncRNA |
| ENSG00000171860.5 | 28.9437223 | 1.04389448 | 0.31744546 | 3.28842148 | 0.00100751 | 0.00500248 | C3AR1 | chr12 | protein_coding |
| ENSG00000284626.1 | 27.762511 | 1.02864423 | 0.29168439 | 3.52656595 | 0.00042099 | 0.00238089 | ENSG00000284626 | chr15 | protein_coding |
| ENSG00000100300.18 | 47.9463344 | 1.14004254 | 0.23202903 | 4.91336166 | 8.95E-07 | 1.05E-05 | TSPO | chr22 | protein_coding |
| ENSG00000137868.19 | 20.1258914 | 1.12338085 | 0.41466741 | 2.70911293 | 0.00674634 | 0.02515075 | STRA6 | chr15 | protein_coding |
| ENSG00000198933.11 | 169.420827 | 1.16136055 | 0.33990177 | 3.41675341 | 0.00063373 | 0.00336924 | TBKBP1 | chr17 | protein_coding |
| ENSG00000264569.2 | 27.2291245 | 1.34135536 | 0.31786429 | 4.21989957 | 2.44E-05 | 0.0001982 | DCXR-DT | chr17 | lncRNA |
| ENSG00000100427.16 | 16.2479693 | -1.0351295 | 0.4183653 | -2.4742242 | 0.01335259 | 0.0443169 | MLC1 | chr22 | protein_coding |
| ENSG00000288885.2 | 14.8018295 | 1.38144044 | 0.44492156 | 3.10490784 | 0.00190338 | 0.00864582 | ENSG00000288885 | chr21 | lncRNA |
| ENSG00000229278.4 | 9.12126867 | 1.47773703 | 0.55045598 | 2.68456893 | 0.00726234 | 0.0267672 | ENSG00000229278 | chr10 | lncRNA |
| ENSG00000166578.10 | 17.9458615 | 1.20815559 | 0.39174615 | 3.08402673 | 0.00204219 | 0.00916361 | IQCD | chr12 | protein_coding |
| ENSG00000204929.13 | 5.89591971 | 1.83855098 | 0.7136717 | 2.57618591 | 0.00998969 | 0.0350744 | LINC02934 | chr2 | lncRNA |
| ENSG00000229236.3 | 12.7041966 | 1.20649985 | 0.45073643 | 2.67673029 | 0.00743445 | 0.02730688 | TTTY10 | chrY | lncRNA |
| ENSG00000177807.10 | 35.2582806 | 1.28958541 | 0.27377909 | 4.71031372 | 2.47E-06 | 2.60E-05 | KCNJ10 | chr1 | protein_coding |
| ENSG00000100292.18 | 5050.07403 | -1.639788 | 0.06033364 | -27.178667 | 1.16E-162 | 2.45E-158 | HMOX1 | chr22 | protein_coding |
| ENSG00000289500.2 | 5.4909069 | 1.720405 | 0.70799244 | 2.42997649 | 0.0150998 | 0.04905925 | ENSG00000289500 | chr3 | lncRNA |
| ENSG00000270076.1 | 37.3196415 | -1.2209361 | 0.27638337 | -4.4175455 | 9.98E-06 | 8.96E-05 | ENSG00000270076 | chr8 | lncRNA |
| ENSG00000139182.15 | 406.408403 | 1.11602837 | 0.10825093 | 10.3096426 | 6.37E-25 | 1.58E-22 | CLSTN3 | chr12 | protein_coding |
| ENSG00000166828.3 | 154.210457 | -1.932047 | 0.15822109 | -12.211059 | 2.71E-34 | 1.98E-31 | SCNN1G | chr16 | protein_coding |
| ENSG00000100298.16 | 57.008864 | 2.47162352 | 0.26917651 | 9.18216634 | 4.23E-20 | 5.99E-18 | APOBEC3H | chr22 | protein_coding |
| ENSG00000160179.19 | 75.0775652 | 1.2233872 | 0.19974675 | 6.12469127 | 9.09E-10 | 2.09E-08 | ABCG1 | chr21 | protein_coding |
| ENSG00000144821.11 | 55.6845689 | 1.0079693 | 0.2070373 | 4.86853956 | 1.12E-06 | 1.29E-05 | MYH15 | chr3 | protein_coding |
| ENSG00000124507.11 | 90.0068678 | 1.11293846 | 0.19967873 | 5.57364549 | 2.49E-08 | 4.21E-07 | PACSIN1 | chr6 | protein_coding |
| ENSG00000099953.10 | 25.2345417 | 1.04929192 | 0.3189946 | 3.28937208 | 0.00100411 | 0.00498913 | MMP11 | chr22 | protein_coding |
| ENSG00000259868.3 | 8.44173161 | 1.75617566 | 0.66996169 | 2.62130757 | 0.00875932 | 0.03133821 | ENSG00000259868 | chr14 | lncRNA |
| ENSG00000269190.6 | 40.3014676 | 1.24786166 | 0.31912266 | 3.91028852 | 9.22E-05 | 0.00063389 | FBXO17 | chr19 | protein_coding |
| ENSG00000170915.9 | 103.750739 | 1.07707702 | 0.21195083 | 5.08173068 | 3.74E-07 | 4.84E-06 | PAQR8 | chr6 | protein_coding |
| ENSG00000259207.10 | 35.6754622 | 1.30241089 | 0.26326788 | 4.94709376 | 7.53E-07 | 9.10E-06 | ITGB3 | chr17 | protein_coding |
| ENSG00000272483.1 | 9.49295183 | -1.461628 | 0.51443516 | -2.8412287 | 0.00449401 | 0.01791493 | ENSG00000272483 | chr3 | lncRNA |
| ENSG00000183625.16 | 59.4176835 | 1.2516362 | 0.21735534 | 5.75847907 | 8.49E-09 | 1.56E-07 | CCR3 | chr3 | protein_coding |
| ENSG00000254726.3 | 22.2820248 | 1.00383859 | 0.33579354 | 2.98945182 | 0.00279479 | 0.01195371 | MEX3A | chr1 | protein_coding |
| ENSG00000170537.13 | 10.3880214 | 1.42100252 | 0.49536851 | 2.86857663 | 0.00412323 | 0.0166443 | TMC7 | chr16 | protein_coding |
| ENSG00000259772.8 | 4.1350752 | 2.45292909 | 0.92113764 | 2.66293438 | 0.00774625 | 0.02823043 | LINC03034 | chr15 | lncRNA |
| ENSG00000196517.13 | 566.119079 | -1.108049 | 0.19476642 | -5.6891176 | 1.28E-08 | 2.27E-07 | SLC6A9 | chr1 | protein_coding |
| ENSG00000124313.19 | 25.1839074 | 1.03061363 | 0.34888755 | 2.95399949 | 0.00313685 | 0.01324237 | IQSEC2 | chrX | protein_coding |
| ENSG00000186854.11 | 7.11486859 | 1.84457513 | 0.62509808 | 2.95085711 | 0.00316893 | 0.01335209 | TRABD2A | chr2 | protein_coding |
| ENSG00000111801.16 | 58.814871 | 1.08205155 | 0.2410995 | 4.4879875 | 7.19E-06 | 6.69E-05 | BTN3A3 | chr6 | protein_coding |
| ENSG00000134240.12 | 6.91624889 | 2.33388695 | 0.68330541 | 3.41558388 | 0.00063645 | 0.00338289 | HMGCS2 | chr1 | protein_coding |
| ENSG00000181004.10 | 96.16891 | 1.02117499 | 0.19517628 | 5.2320651 | 1.68E-07 | 2.35E-06 | BBS12 | chr4 | protein_coding |
| ENSG00000035664.11 | 17.2523813 | 1.09421491 | 0.39495806 | 2.77045844 | 0.00559774 | 0.02147939 | DAPK2 | chr15 | protein_coding |
| ENSG00000086619.15 | 51.479461 | 1.01303473 | 0.25109692 | 4.03443716 | 5.47E-05 | 0.00040137 | ERO1B | chr1 | protein_coding |
| ENSG00000204681.11 | 63.3422259 | 1.13561982 | 0.21640454 | 5.24767087 | 1.54E-07 | 2.18E-06 | GABBR1 | chr6 | protein_coding |
| ENSG00000124243.19 | 72.8856666 | 1.10156692 | 0.21331567 | 5.16402237 | 2.42E-07 | 3.26E-06 | BCAS4 | chr20 | protein_coding |
| ENSG00000183690.13 | 17.7491416 | 1.29641431 | 0.37561625 | 3.45143292 | 0.00055762 | 0.00303179 | EFHC2 | chrX | protein_coding |
| ENSG00000197757.8 | 11.4344469 | 1.9922634 | 0.54156204 | 3.67873531 | 0.00023439 | 0.00144356 | HOXC6 | chr12 | protein_coding |
| ENSG00000290803.1 | 19.5612375 | 1.21894358 | 0.41312246 | 2.95056238 | 0.00317196 | 0.01336122 | ENSG00000290803 | chr4 | lncRNA |
| ENSG00000114626.18 | 281.179732 | 1.01083912 | 0.12775284 | 7.91245885 | 2.52E-15 | 1.66E-13 | ABTB1 | chr3 | protein_coding |
| ENSG00000161642.18 | 164.478977 | 1.12982073 | 0.15620034 | 7.23315159 | 4.72E-13 | 2.11E-11 | ZNF385A | chr12 | protein_coding |
| ENSG00000167646.14 | 21.0168076 | 1.19239566 | 0.37283322 | 3.19820126 | 0.00138288 | 0.00658145 | DNAAF3 | chr19 | protein_coding |
| ENSG00000142156.16 | 16.2836079 | 1.39165543 | 0.45249967 | 3.07548382 | 0.00210161 | 0.00940178 | COL6A1 | chr21 | protein_coding |
| ENSG00000251576.1 | 3.47084357 | 3.01569134 | 1.09840117 | 2.74552815 | 0.00604136 | 0.02291578 | LINC01267 | chr3 | lncRNA |
| ENSG00000105383.15 | 40.720289 | 1.36375282 | 0.25498992 | 5.34826175 | 8.88E-08 | 1.33E-06 | CD33 | chr19 | protein_coding |
| ENSG00000260879.1 | 40.0689703 | 1.19596261 | 0.25749355 | 4.6446313 | 3.41E-06 | 3.43E-05 | ENSG00000260879 | chr1 | lncRNA |
| ENSG00000109089.7 | 62.7856295 | 1.22690843 | 0.20569559 | 5.96468033 | 2.45E-09 | 5.13E-08 | CDR2L | chr17 | protein_coding |
| ENSG00000147573.17 | 52.3709669 | 1.18289471 | 0.29082402 | 4.06739 | 4.75E-05 | 0.00035542 | TRIM55 | chr8 | protein_coding |
| ENSG00000122735.16 | 19.2914064 | 1.93014763 | 0.44793185 | 4.30902076 | 1.64E-05 | 0.00013968 | DNAI1 | chr9 | protein_coding |
| ENSG00000140368.14 | 73.4559164 | 1.32347074 | 0.24399819 | 5.42410063 | 5.82E-08 | 9.07E-07 | PSTPIP1 | chr15 | protein_coding |
| ENSG00000206077.14 | 49.2626581 | 1.06082421 | 0.24957817 | 4.25046872 | 2.13E-05 | 0.00017599 | ZDHHC11B | chr5 | protein_coding |
| ENSG00000108984.16 | 188.889449 | 1.15103459 | 0.12808742 | 8.98632023 | 2.56E-19 | 3.17E-17 | MAP2K6 | chr17 | protein_coding |
| ENSG00000166268.11 | 62.5399989 | -1.0066018 | 0.21405144 | -4.7026165 | 2.57E-06 | 2.69E-05 | MYRFL | chr12 | protein_coding |
| ENSG00000074211.14 | 41.9471758 | 1.17699345 | 0.2612859 | 4.50461913 | 6.65E-06 | 6.24E-05 | PPP2R2C | chr4 | protein_coding |
| ENSG00000285876.1 | 5.56015568 | 1.91651833 | 0.72311994 | 2.65034638 | 0.00804093 | 0.02917843 | ENSG00000285876 | chr2 | lncRNA |
| ENSG00000104870.13 | 16.5034794 | 1.45719054 | 0.56178476 | 2.59385915 | 0.00949054 | 0.03353937 | FCGRT | chr19 | protein_coding |
| ENSG00000165912.16 | 91.556291 | 1.46462159 | 0.22321574 | 6.56146203 | 5.33E-11 | 1.62E-09 | PACSIN3 | chr11 | protein_coding |
| ENSG00000205593.12 | 21.9658514 | 1.36810202 | 0.35547796 | 3.84862685 | 0.00011878 | 0.00079585 | DENND6B | chr22 | protein_coding |
| ENSG00000108702.4 | 11.0700402 | 1.22062187 | 0.49665574 | 2.457682 | 0.01398369 | 0.04597068 | CCL1 | chr17 | protein_coding |
| ENSG00000101210.14 | 14.5541492 | 1.28692011 | 0.46789682 | 2.75043566 | 0.00595161 | 0.0226397 | EEF1A2 | chr20 | protein_coding |
| ENSG00000286535.1 | 34.579681 | 1.19553234 | 0.26386846 | 4.53078901 | 5.88E-06 | 5.61E-05 | ENSG00000286535 | chr8 | lncRNA |
| ENSG00000133321.11 | 58.1966292 | 1.63240733 | 0.28766381 | 5.67470534 | 1.39E-08 | 2.45E-07 | PLAAT4 | chr11 | protein_coding |
| ENSG00000007866.22 | 91.3591906 | 1.25476289 | 0.17942958 | 6.9930661 | 2.69E-12 | 1.05E-10 | TEAD3 | chr6 | protein_coding |
| ENSG00000157303.11 | 38.7298616 | 1.85928207 | 0.28422969 | 6.54147732 | 6.09E-11 | 1.82E-09 | SUSD3 | chr9 | protein_coding |
| ENSG00000286638.2 | 20.2740565 | 1.02183599 | 0.36186558 | 2.82379988 | 0.0047458 | 0.01875226 | ENSG00000286638 | chr5 | lncRNA |
| ENSG00000174450.13 | 89.848861 | 1.09099885 | 0.16855851 | 6.47252318 | 9.64E-11 | 2.76E-09 | GOLGA6L2 | chr15 | protein_coding |
| ENSG00000211638.2 | 4.09959176 | 3.27608038 | 1.06599791 | 3.07325216 | 0.00211739 | 0.00945683 | IGLV8-61 | chr22 | IG_V_gene |
| ENSG00000289173.2 | 10.1345019 | 1.44767951 | 0.57696757 | 2.50911765 | 0.01210332 | 0.04084482 | ENSG00000289173 | chr12 | lncRNA |
| ENSG00000285051.2 | 2.51049929 | 3.14257341 | 1.29532758 | 2.42608393 | 0.01526274 | 0.04951244 | SLC7A14-AS1 | chr3 | lncRNA |
| ENSG00000127863.16 | 3.65210735 | 3.0837637 | 1.04967033 | 2.9378402 | 0.00330507 | 0.01382264 | TNFRSF19 | chr13 | protein_coding |
| ENSG00000100767.17 | 10.6491564 | 2.05809766 | 0.55790393 | 3.68898216 | 0.00022515 | 0.00139684 | PAPLN | chr14 | protein_coding |
| ENSG00000006047.13 | 21.0103519 | 1.19058784 | 0.42030257 | 2.83269225 | 0.00461578 | 0.01833459 | YBX2 | chr17 | protein_coding |
| ENSG00000138772.13 | 9.77111322 | 1.49195774 | 0.52192403 | 2.85857263 | 0.00425552 | 0.01712264 | ANXA3 | chr4 | protein_coding |
| ENSG00000135643.5 | 12.7884204 | 1.13863571 | 0.4458891 | 2.55362982 | 0.01066065 | 0.0368903 | KCNMB4 | chr12 | protein_coding |
| ENSG00000119915.5 | 18.5682119 | 1.380396 | 0.39775946 | 3.47042912 | 0.00051963 | 0.00285131 | ELOVL3 | chr10 | protein_coding |
| ENSG00000140263.15 | 72.9977067 | 1.34582801 | 0.18650347 | 7.21610177 | 5.35E-13 | 2.37E-11 | SORD | chr15 | protein_coding |
| ENSG00000279278.1 | 5.74636412 | -1.7393159 | 0.71351411 | -2.4376756 | 0.01478203 | 0.04816766 | ENSG00000279278 | chr22 | lncRNA |
| ENSG00000259684.1 | 8.23067846 | -1.4995804 | 0.57773889 | -2.5956023 | 0.00944253 | 0.03339767 | ENSG00000259684 | chr15 | lncRNA |
| ENSG00000265972.6 | 1412.84951 | 1.84653325 | 0.18150053 | 10.1737074 | 2.60E-24 | 6.16E-22 | TXNIP | chr1 | protein_coding |
| ENSG00000232453.7 | 13.4595833 | 1.185504 | 0.45214666 | 2.6219457 | 0.00874294 | 0.0312955 | LINC02777 | chr1 | lncRNA |
| ENSG00000265055.2 | 26.0555678 | 1.44046256 | 0.32072737 | 4.4912368 | 7.08E-06 | 6.60E-05 | ENSG00000265055 | chr17 | lncRNA |
| ENSG00000172794.20 | 907.588036 | 1.2048122 | 0.07129709 | 16.8984761 | 4.62E-64 | 1.62E-60 | RAB37 | chr17 | protein_coding |
| ENSG00000105467.9 | 11.8720012 | 1.33754387 | 0.49875653 | 2.68175713 | 0.00732366 | 0.02696027 | SYNGR4 | chr19 | protein_coding |
| ENSG00000282021.1 | 4.93210451 | 1.91457354 | 0.78381199 | 2.44264386 | 0.01458012 | 0.04760523 | ENSG00000282021 | chr8 | lncRNA |
| ENSG00000019505.8 | 19.6395006 | 1.60008221 | 0.36195105 | 4.42071438 | 9.84E-06 | 8.85E-05 | SYT13 | chr11 | protein_coding |
| ENSG00000183287.15 | 7.44695814 | 1.6977151 | 0.66470514 | 2.55408752 | 0.01064665 | 0.03686601 | CCBE1 | chr18 | protein_coding |
| ENSG00000228218.1 | 18.4410228 | -1.1403951 | 0.35483257 | -3.2138965 | 0.00130947 | 0.00628307 | ATF4P3 | chr17 | processed_pseudogene |
| ENSG00000269296.1 | 9.5076622 | 1.24878517 | 0.50841694 | 2.4562226 | 0.01404062 | 0.04612908 | ENSG00000269296 | chr19 | lncRNA |
| ENSG00000186891.14 | 10.5559416 | 1.26824718 | 0.49266692 | 2.57424873 | 0.0100458 | 0.03522454 | TNFRSF18 | chr1 | protein_coding |
| ENSG00000187492.9 | 9.67796864 | 1.28640502 | 0.50330312 | 2.55592498 | 0.01059059 | 0.03673327 | CDHR4 | chr3 | protein_coding |
| ENSG00000116885.18 | 45.213251 | 1.1807802 | 0.27356273 | 4.3163051 | 1.59E-05 | 0.00013583 | OSCP1 | chr1 | protein_coding |
| ENSG00000182534.14 | 264.598534 | 1.47555504 | 0.12992535 | 11.3569449 | 6.85E-30 | 2.84E-27 | MXRA7 | chr17 | protein_coding |
| ENSG00000163576.18 | 20.405887 | 1.03108506 | 0.36385093 | 2.83381178 | 0.00459964 | 0.01828769 | EFHB | chr3 | protein_coding |
| ENSG00000185686.18 | 475.805074 | 1.0844677 | 0.08909182 | 12.1724721 | 4.36E-34 | 3.07E-31 | PRAME | chr22 | protein_coding |
| ENSG00000159761.15 | 43.0748717 | 1.19527365 | 0.26094801 | 4.5805049 | 4.64E-06 | 4.54E-05 | C16orf86 | chr16 | protein_coding |
| ENSG00000188487.12 | 54.7341552 | 1.00352372 | 0.23796842 | 4.21704581 | 2.48E-05 | 0.00020022 | INSC | chr11 | protein_coding |
| ENSG00000183773.16 | 14.2903076 | 1.21725899 | 0.4895169 | 2.48665367 | 0.01289509 | 0.04303526 | AIFM3 | chr22 | protein_coding |
| ENSG00000283440.1 | 75.0358954 | 1.0681762 | 0.21131219 | 5.05496731 | 4.30E-07 | 5.50E-06 | LINC01260 | chr20 | lncRNA |
| ENSG00000196372.13 | 136.194796 | 1.0317453 | 0.17136095 | 6.0208892 | 1.73E-09 | 3.74E-08 | ASB13 | chr10 | protein_coding |
|  |  |  |  |  |  |  |  |  |  |
