# Supplementary material for: Bone marrow stromal cells enhance multiple myeloma cells proliferation through regulating LncRNA OVAAL/ENPP1 axis
Source: Open Life Sci. 2026 May 14;21(1):20251322. doi: 10.1515/biol-2025-1322 (PMC13170762; doi:10.1515/biol-2025-1322)
Supplement: Supplementary file 2 — Supplementary Material [file j_biol-2025-1322_suppl_002.docx]

| Supplementary 2 Pathway enrichment analysis of upregulated differentially expressed mRNAs between U266 and co-cultured system. | | | | | | |
| --- | --- | --- | --- | --- | --- | --- |
| Term | ID | Input number | Background number | P-Value | Corrected P-Value | Input |
| Complement and coagulation cascades | hsa04610 | 8 | 79 | 0.000325 | 0.073048 | ITGAX\|SERPINA1\|F3\|CFI\|C3AR1\|MASP1\|PLAU\|CPB2 |
| Protein digestion and absorption | hsa04974 | 7 | 90 | 0.003585 | 0.403309 | COL13A1\|COL9A2\|CPB2\|COL27A1\|KCNN4\|COL1A1\|COL6A1 |
| Arrhythmogenic right ventricular cardiomyopathy (ARVC) | hsa05412 | 6 | 77 | 0.006825 | 0.441585 | ITGB3\|CACNB3\|TCF7L2\|CACNG8\|CACNA2D2\|CACNG7 |
| Pantothenate and CoA biosynthesis | hsa00770 | 3 | 19 | 0.00785 | 0.441585 | UPB1\|ENPP3\|ENPP1 |
| Cardiac muscle contraction | hsa04260 | 6 | 86 | 0.011516 | 0.441971 | TRDN\|CACNB3\|CACNG8\|CACNA2D2\|COX6B2\|CACNG7 |
| Riboflavin metabolism | hsa00740 | 2 | 8 | 0.012378 | 0.441971 | ENPP3\|ENPP1 |
| Hematopoietic cell lineage | hsa04640 | 6 | 97 | 0.019888 | 0.441971 | ITGB3\|CD33\|CD7\|CD8A\|IL1R1\|CD19 |
| Inflammatory mediator regulation of TRP channels | hsa04750 | 6 | 100 | 0.022744 | 0.441971 | ASIC1\|PRKCG\|MAP2K6\|PLA2G4D\|IL1R1\|TRPV2 |
| Viral protein interaction with cytokine and cytokine receptor | hsa04061 | 6 | 100 | 0.022744 | 0.441971 | IL2RB\|CCL1\|TNFRSF1B\|CCR3\|CCR5\|CCR7 |
| Cholesterol metabolism | hsa04979 | 4 | 50 | 0.023851 | 0.441971 | ANGPTL4\|TSPO\|ABCA1\|LRP1 |
| Hippo signaling pathway - multiple species | hsa04392 | 3 | 29 | 0.025221 | 0.441971 | TEAD3\|AJUBA\|RASSF4 |
| Amyotrophic lateral sclerosis (ALS) | hsa05014 | 4 | 51 | 0.025449 | 0.441971 | NOS1\|TNFRSF1B\|MAP2K6\|SLC1A2 |
| Nicotinate and nicotinamide metabolism | hsa00760 | 3 | 30 | 0.027581 | 0.441971 | ENPP3\|NT5C1A\|ENPP1 |
| Cytokine-cytokine receptor interaction | hsa04060 | 12 | 294 | 0.028802 | 0.441971 | IL1RN\|IL13RA1\|CCL1\|TNFRSF1B\|IL12RB2\|IL2RB\|TNFRSF18\|CCR3\|CCR5\|CCR7\|IL1R1\|TNFRSF19 |
| MAPK signaling pathway | hsa04010 | 12 | 295 | 0.029465 | 0.441971 | ERBB3\|MAP2K6\|NR4A1\|PRKCG\|CACNG8\|CACNB3\|FGF20\|MAPT\|CACNA2D2\|PLA2G4D\|IL1R1\|CACNG7 |
| Pyrimidine metabolism | hsa00240 | 4 | 57 | 0.036377 | 0.511557 | UPB1\|ENPP3\|NT5C1A\|ENPP1 |
| ECM-receptor interaction | hsa04512 | 5 | 86 | 0.040815 | 0.540194 | COL6A1\|ITGB3\|TNN\|COL9A2\|COL1A1 |
| Hypertrophic cardiomyopathy (HCM) | hsa05410 | 5 | 90 | 0.048074 | 0.573596 | CACNA2D2\|CACNG8\|ITGB3\|CACNB3\|CACNG7 |
| PI3K-Akt signaling pathway | hsa04151 | 13 | 354 | 0.048437 | 0.573596 | ERBB3\|ITGB3\|NR4A1\|PPP2R3A\|COL9A2\|IL2RB\|PPP2R2C\|PIK3AP1\|COL1A1\|COL6A1\|TNN\|FGF20\|CD19 |
| Oxytocin signaling pathway | hsa04921 | 7 | 153 | 0.051911 | 0.583994 | KCNJ4\|CACNB3\|PRKCG\|CACNG8\|CACNA2D2\|CACNG7\|PLA2G4D |
| Dilated cardiomyopathy (DCM) | hsa05414 | 5 | 96 | 0.060311 | 0.646187 | CACNA2D2\|CACNG8\|ITGB3\|CACNB3\|CACNG7 |
| Purine metabolism | hsa00230 | 6 | 130 | 0.066982 | 0.685043 | ENTPD2\|ENPP3\|ENPP1\|NT5C1A\|PDE4B\|PDE5A |
| Focal adhesion | hsa04510 | 8 | 199 | 0.072476 | 0.693711 | ITGB3\|TLN2\|COL9A2\|PRKCG\|COL1A1\|COL6A1\|TNN\|CAV2 |
| ABC transporters | hsa02010 | 3 | 45 | 0.076091 | 0.693711 | ABCG1\|ABCA2\|ABCA1 |
| Arginine biosynthesis | hsa00220 | 2 | 21 | 0.077079 | 0.693711 | NOS1\|GLS2 |
| Ether lipid metabolism | hsa00565 | 3 | 47 | 0.084278 | 0.729327 | GAL3ST1\|GDPD1\|PLA2G4D |
| Mannose type O-glycan biosynthesis | hsa00515 | 2 | 23 | 0.090278 | 0.752319 | LARGE2\|MGAT5B |
| Arginine and proline metabolism | hsa00330 | 3 | 50 | 0.097226 | 0.781283 | NOS1\|AZIN2\|CKB |
| D-Glutamine and D-glutamate metabolism | hsa00471 | 1 | 5 | 0.105414 | 0.784846 | GLS2 |
| Breast cancer | hsa05224 | 6 | 147 | 0.105531 | 0.784846 | FZD4\|TCF7L2\|FRAT1\|DLL4\|FGF20\|HEY1 |
| Adrenergic signaling in cardiomyocytes | hsa04261 | 6 | 149 | 0.110697 | 0.784846 | CACNB3\|PPP2R3A\|PPP2R2C\|CACNG8\|CACNA2D2\|CACNG7 |
| Human papillomavirus infection | hsa05165 | 11 | 330 | 0.111623 | 0.784846 | ITGB3\|FZD4\|HEY1\|PPP2R3A\|COL9A2\|PPP2R2C\|TCF7L2\|COL1A1\|COL6A1\|TNN\|HES7 |
| Hippo signaling pathway | hsa04390 | 6 | 154 | 0.124167 | 0.84659 | FZD4\|CCN2\|PPP2R2C\|TCF7L2\|TEAD3\|AJUBA |
| Salivary secretion | hsa04970 | 4 | 90 | 0.136599 | 0.884697 | NOS1\|KCNN4\|PRKCG\|DMBT1 |
| Pentose phosphate pathway | hsa00030 | 2 | 30 | 0.140748 | 0.884697 | TKTL1\|FBP1 |
| Th1 and Th2 cell differentiation | hsa04658 | 4 | 92 | 0.144608 | 0.884697 | IL2RB\|IL12RB2\|DLL4\|STAT5A |
| Long-term depression | hsa04730 | 3 | 60 | 0.145484 | 0.884697 | NOS1\|PRKCG\|PLA2G4D |
| Basal cell carcinoma | hsa05217 | 3 | 63 | 0.161258 | 0.917177 | GLI1\|FZD4\|TCF7L2 |
| Fructose and mannose metabolism | hsa00051 | 2 | 33 | 0.163889 | 0.917177 | SORD\|FBP1 |
| SNARE interactions in vesicular transport | hsa04130 | 2 | 34 | 0.171748 | 0.917177 | STX1B\|VAMP5 |
| Choline metabolism in cancer | hsa05231 | 4 | 99 | 0.174008 | 0.917177 | SLC44A2\|SLC44A3\|PRKCG\|PLA2G4D |
| AGE-RAGE signaling pathway in diabetic complications | hsa04933 | 4 | 100 | 0.178368 | 0.917177 | STAT5A\|EGR1\|F3\|COL1A1 |
| Starch and sucrose metabolism | hsa00500 | 2 | 36 | 0.187642 | 0.917177 | ENPP3\|ENPP1 |
| Alanine, aspartate and glutamate metabolism | hsa00250 | 2 | 36 | 0.187642 | 0.917177 | NAT8L\|GLS2 |
| Fc epsilon RI signaling pathway | hsa04664 | 3 | 68 | 0.188608 | 0.917177 | ALOX5AP\|MAP2K6\|PLA2G4D |
| Staphylococcus aureus infection | hsa05150 | 3 | 68 | 0.188608 | 0.917177 | C3AR1\|MASP1\|CFI |
| Primary immunodeficiency | hsa05340 | 2 | 37 | 0.195665 | 0.917177 | CD8A\|CD19 |
| African trypanosomiasis | hsa05143 | 2 | 37 | 0.195665 | 0.917177 | PRKCG\|APOL1 |
| Synthesis and degradation of ketone bodies | hsa00072 | 1 | 10 | 0.199774 | 0.91733 | HMGCS2 |
| Transcriptional misregulation in cancer | hsa05202 | 6 | 186 | 0.226785 | 0.952836 | MLF1\|ETV1\|IL2RB\|GZMB\|PLAU\|BCL6 |
| Gastric acid secretion | hsa04971 | 3 | 75 | 0.228665 | 0.952836 | SLC9A4\|KCNJ10\|PRKCG |
| PPAR signaling pathway | hsa03320 | 3 | 76 | 0.23452 | 0.952836 | FABP6\|HMGCS2\|ANGPTL4 |
| Chemokine signaling pathway | hsa04062 | 6 | 190 | 0.241239 | 0.952836 | CCL1\|ITK\|FGR\|CCR3\|CCR5\|CCR7 |
| Glutamatergic synapse | hsa04724 | 4 | 114 | 0.242797 | 0.952836 | PRKCG\|SLC1A2\|GLS2\|PLA2G4D |
| Thyroid hormone signaling pathway | hsa04919 | 4 | 119 | 0.267004 | 0.952836 | ITGB3\|DIO1\|PRKCG\|MED12L |
| Glycosaminoglycan biosynthesis - keratan sulfate | hsa00533 | 1 | 14 | 0.268082 | 0.952836 | B3GNT7 |
| Wnt signaling pathway | hsa04310 | 5 | 160 | 0.277145 | 0.952836 | FRAT1\|FZD4\|CTNND2\|PRKCG\|TCF7L2 |
| ErbB signaling pathway | hsa04012 | 3 | 85 | 0.288183 | 0.952836 | ERBB3\|STAT5A\|PRKCG |
| Proteoglycans in cancer | hsa05205 | 6 | 203 | 0.289933 | 0.952836 | ERBB3\|ITGB3\|FZD4\|PLAU\|PRKCG\|CAV2 |
| Platelet activation | hsa04611 | 4 | 124 | 0.291631 | 0.952836 | ITGB3\|PLA2G4D\|TLN2\|COL1A1 |
| Insulin secretion | hsa04911 | 3 | 86 | 0.294221 | 0.952836 | KCNN4\|KCNMB4\|PRKCG |
| Osteoclast differentiation | hsa04380 | 4 | 128 | 0.311552 | 0.952836 | SIRPG\|ITGB3\|MAP2K6\|IL1R1 |
| GABAergic synapse | hsa04727 | 3 | 89 | 0.312376 | 0.952836 | GABBR1\|PRKCG\|GLS2 |
| Selenocompound metabolism | hsa00450 | 1 | 17 | 0.315478 | 0.952836 | INMT |
| Morphine addiction | hsa05032 | 3 | 91 | 0.324501 | 0.952836 | GABBR1\|PDE4B\|PRKCG |
| mRNA surveillance pathway | hsa03015 | 3 | 91 | 0.324501 | 0.952836 | MSI1\|PPP2R3A\|PPP2R2C |
| Alzheimer disease | hsa05010 | 5 | 171 | 0.324596 | 0.952836 | NOS1\|COX6B2\|LRP1\|BACE2\|MAPT |
| Human immunodeficiency virus 1 infection | hsa05170 | 6 | 212 | 0.324793 | 0.952836 | TNFRSF1B\|APOBEC3H\|APOBEC3G\|PRKCG\|MAP2K6\|CCR5 |
| Fatty acid biosynthesis | hsa00061 | 1 | 18 | 0.330589 | 0.952836 | OLAH |
| Vascular smooth muscle contraction | hsa04270 | 4 | 132 | 0.331602 | 0.952836 | ACTA2\|KCNMB4\|PRKCG\|PLA2G4D |
| GnRH signaling pathway | hsa04912 | 3 | 93 | 0.336628 | 0.952836 | EGR1\|MAP2K6\|PLA2G4D |
| Fc gamma R-mediated phagocytosis | hsa04666 | 3 | 94 | 0.342688 | 0.952836 | PLA2G4D\|PRKCG\|ASAP3 |
| Amoebiasis | hsa05146 | 3 | 95 | 0.348744 | 0.952836 | PRKCG\|IL1R1\|COL1A1 |
| Human T-cell leukemia virus 1 infection | hsa05166 | 6 | 219 | 0.352311 | 0.952836 | IL2RB\|EGR1\|TLN2\|STAT5A\|TSPO\|IL1R1 |
| NOD-like receptor signaling pathway | hsa04621 | 5 | 178 | 0.355275 | 0.952836 | TXNIP\|GBP2\|PSTPIP1\|NLRP6\|TRPV2 |
| Apelin signaling pathway | hsa04371 | 4 | 137 | 0.356754 | 0.952836 | ACTA2\|NOS1\|EGR1\|CCN2 |
| Glycerophospholipid metabolism | hsa00564 | 3 | 97 | 0.36084 | 0.952836 | GPD1L\|ETNPPL\|PLA2G4D |
| Fluid shear stress and atherosclerosis | hsa05418 | 4 | 139 | 0.366821 | 0.952836 | ITGB3\|CAV2\|MAP2K6\|IL1R1 |
| Axon guidance | hsa04360 | 5 | 181 | 0.368474 | 0.952836 | PLXNB2\|PLXNB3\|EPHB6\|SEMA3G\|SEMA3F |
| VEGF signaling pathway | hsa04370 | 2 | 59 | 0.374584 | 0.952836 | PRKCG\|PLA2G4D |
| Human cytomegalovirus infection | hsa05163 | 6 | 225 | 0.376055 | 0.952836 | ITGB3\|PRKCG\|CCR3\|CCR5\|IL1R1\|MAP2K6 |
| Melanogenesis | hsa04916 | 3 | 101 | 0.384925 | 0.952836 | FZD4\|TCF7L2\|PRKCG |
| Pathways in cancer | hsa05200 | 13 | 530 | 0.384953 | 0.952836 | IL13RA1\|FZD4\|IL12RB2\|IL2RB\|PRKCG\|FRAT1\|STAT5A\|GLI1\|DLL4\|TCF7L2\|DAPK2\|FGF20\|HEY1 |
| Terpenoid backbone biosynthesis | hsa00900 | 1 | 22 | 0.387787 | 0.952836 | HMGCS2 |
| Proximal tubule bicarbonate reclamation | hsa04964 | 1 | 23 | 0.40131 | 0.952836 | GLS2 |
| Vitamin digestion and absorption | hsa04977 | 1 | 24 | 0.414536 | 0.952836 | CUBN |
| Parathyroid hormone synthesis, secretion and action | hsa04928 | 3 | 106 | 0.41473 | 0.952836 | PDE4B\|EGR1\|PRKCG |
| Gastric cancer | hsa05226 | 4 | 149 | 0.416931 | 0.952836 | FRAT1\|FZD4\|TCF7L2\|FGF20 |
| Th17 cell differentiation | hsa04659 | 3 | 107 | 0.42064 | 0.952836 | IL2RB\|STAT5A\|IL1R1 |
| alpha-Linolenic acid metabolism | hsa00592 | 1 | 25 | 0.427471 | 0.952836 | PLA2G4D |
| Non-small cell lung cancer | hsa05223 | 2 | 66 | 0.428929 | 0.952836 | STAT5A\|PRKCG |
| Acute myeloid leukemia | hsa05221 | 2 | 66 | 0.428929 | 0.952836 | STAT5A\|TCF7L2 |
| Cholinergic synapse | hsa04725 | 3 | 112 | 0.449875 | 0.952836 | CHRNB4\|PRKCG\|KCNJ4 |
| Leukocyte transendothelial migration | hsa04670 | 3 | 112 | 0.449875 | 0.952836 | CLDN4\|ITK\|PRKCG |
| Endocytosis | hsa04144 | 6 | 244 | 0.45116 | 0.952836 | IL2RB\|CCR5\|WIPF3\|IQSEC2\|CAV2\|ASAP3 |
| Biosynthesis of unsaturated fatty acids | hsa01040 | 1 | 27 | 0.452496 | 0.952836 | ELOVL3 |
| Fatty acid elongation | hsa00062 | 1 | 27 | 0.452496 | 0.952836 | ELOVL3 |
| Viral carcinogenesis | hsa05203 | 5 | 201 | 0.45599 | 0.952836 | HDAC11\|STAT5A\|CCR3\|CCR5\|HPN |
| Butanoate metabolism | hsa00650 | 1 | 28 | 0.464597 | 0.952836 | HMGCS2 |
| Adherens junction | hsa04520 | 2 | 72 | 0.473489 | 0.952836 | PTPRJ\|TCF7L2 |
| Linoleic acid metabolism | hsa00591 | 1 | 29 | 0.476433 | 0.952836 | PLA2G4D |
| Jak-STAT signaling pathway | hsa04630 | 4 | 162 | 0.480627 | 0.952836 | IL13RA1\|IL12RB2\|IL2RB\|STAT5A |
| Bacterial invasion of epithelial cells | hsa05100 | 2 | 74 | 0.487876 | 0.952836 | SEPTIN9\|CAV2 |
| Sphingolipid signaling pathway | hsa04071 | 3 | 119 | 0.489767 | 0.952836 | PPP2R3A\|PRKCG\|PPP2R2C |
| Rap1 signaling pathway | hsa04015 | 5 | 210 | 0.49442 | 0.952836 | PRKCG\|ITGB3\|TLN2\|MAP2K6\|FGF20 |
| AMPK signaling pathway | hsa04152 | 3 | 120 | 0.495353 | 0.952836 | PPP2R3A\|FBP1\|PPP2R2C |
| Hepatocellular carcinoma | hsa05225 | 4 | 168 | 0.509136 | 0.952836 | FRAT1\|FZD4\|TCF7L2\|PRKCG |
| Autophagy - other | hsa04136 | 1 | 32 | 0.510401 | 0.952836 | ATG9B |
| Synaptic vesicle cycle | hsa04721 | 2 | 78 | 0.515906 | 0.952836 | STX1B\|SLC1A2 |
| Tight junction | hsa04530 | 4 | 170 | 0.518486 | 0.952836 | CLDN4\|SYNPO\|MYH15\|PPP2R2C |
| beta-Alanine metabolism | hsa00410 | 1 | 33 | 0.52123 | 0.952836 | UPB1 |
| Apoptosis - multiple species | hsa04215 | 1 | 33 | 0.52123 | 0.952836 | SEPTIN4 |
| EGFR tyrosine kinase inhibitor resistance | hsa01521 | 2 | 79 | 0.522755 | 0.952836 | ERBB3\|PRKCG |
| Pentose and glucuronate interconversions | hsa00040 | 1 | 34 | 0.53182 | 0.952836 | SORD |
| Prion diseases | hsa05020 | 1 | 35 | 0.542177 | 0.952836 | EGR1 |
| B cell receptor signaling pathway | hsa04662 | 2 | 82 | 0.54291 | 0.952836 | PIK3AP1\|CD19 |
| Relaxin signaling pathway | hsa04926 | 3 | 130 | 0.54946 | 0.952836 | ACTA2\|NOS1\|COL1A1 |
| Taste transduction | hsa04742 | 2 | 83 | 0.549497 | 0.952836 | GABBR1\|ENTPD2 |
| Natural killer cell mediated cytotoxicity | hsa04650 | 3 | 131 | 0.554684 | 0.952836 | CD244\|PRKCG\|GZMB |
| Dopaminergic synapse | hsa04728 | 3 | 131 | 0.554684 | 0.952836 | PPP2R3A\|PRKCG\|PPP2R2C |
| Aldosterone-regulated sodium reabsorption | hsa04960 | 1 | 37 | 0.562214 | 0.952836 | PRKCG |
| Thyroid cancer | hsa05216 | 1 | 37 | 0.562214 | 0.952836 | TCF7L2 |
| Allograft rejection | hsa05330 | 1 | 38 | 0.571903 | 0.952836 | GZMB |
| Pyruvate metabolism | hsa00620 | 1 | 39 | 0.581378 | 0.952836 | LDHD |
| Gap junction | hsa04540 | 2 | 88 | 0.58143 | 0.952836 | TUBB4A\|PRKCG |
| Graft-versus-host disease | hsa05332 | 1 | 41 | 0.599708 | 0.952836 | GZMB |
| Bladder cancer | hsa05219 | 1 | 41 | 0.599708 | 0.952836 | DAPK2 |
| Fat digestion and absorption | hsa04975 | 1 | 41 | 0.599708 | 0.952836 | ABCA1 |
| Tryptophan metabolism | hsa00380 | 1 | 42 | 0.608572 | 0.952836 | INMT |
| Type I diabetes mellitus | hsa04940 | 1 | 43 | 0.61724 | 0.952836 | GZMB |
| TGF-beta signaling pathway | hsa04350 | 2 | 94 | 0.617529 | 0.952836 | THSD4\|CHRD |
| Calcium signaling pathway | hsa04020 | 4 | 193 | 0.619375 | 0.952836 | TRDN\|ERBB3\|PRKCG\|NOS1 |
| Carbohydrate digestion and absorption | hsa04973 | 1 | 44 | 0.625717 | 0.952836 | SLC2A5 |
| Cell adhesion molecules (CAMs) | hsa04514 | 3 | 146 | 0.628572 | 0.952836 | CD8A\|CLDN4\|NRXN3 |
| Circadian entrainment | hsa04713 | 2 | 97 | 0.634668 | 0.952836 | NOS1\|PRKCG |
| Prostate cancer | hsa05215 | 2 | 97 | 0.634668 | 0.952836 | TCF7L2\|PLAU |
| Pancreatic secretion | hsa04972 | 2 | 98 | 0.640247 | 0.952836 | CPB2\|PRKCG |
| Aldosterone synthesis and secretion | hsa04925 | 2 | 98 | 0.640247 | 0.952836 | NR4A1\|PRKCG |
| Hedgehog signaling pathway | hsa04340 | 1 | 47 | 0.650046 | 0.952836 | GLI1 |
| Sphingolipid metabolism | hsa00600 | 1 | 47 | 0.650046 | 0.952836 | GAL3ST1 |
| MicroRNAs in cancer | hsa05206 | 6 | 299 | 0.65024 | 0.952836 | ERBB3\|ITGB3\|PLAU\|PRKCG\|TNN\|GLS2 |
| NF-kappa B signaling pathway | hsa04064 | 2 | 100 | 0.651205 | 0.952836 | PLAU\|IL1R1 |
| Phagosome | hsa04145 | 3 | 152 | 0.655679 | 0.952836 | NOS1\|TUBB4A\|ITGB3 |
| Valine, leucine and isoleucine degradation | hsa00280 | 1 | 48 | 0.657801 | 0.952836 | HMGCS2 |
| Notch signaling pathway | hsa04330 | 1 | 48 | 0.657801 | 0.952836 | DLL4 |
| mTOR signaling pathway | hsa04150 | 3 | 153 | 0.660057 | 0.952836 | CASTOR1\|FZD4\|PRKCG |
| Malaria | hsa05144 | 1 | 49 | 0.665385 | 0.952836 | LRP1 |
| Ovarian steroidogenesis | hsa04913 | 1 | 49 | 0.665385 | 0.952836 | PLA2G4D |
| T cell receptor signaling pathway | hsa04660 | 2 | 103 | 0.667145 | 0.952836 | CD8A\|ITK |
| Cushing syndrome | hsa04934 | 3 | 155 | 0.668694 | 0.952836 | NR4A1\|FZD4\|TCF7L2 |
| Vibrio cholerae infection | hsa05110 | 1 | 50 | 0.672802 | 0.952836 | KDELR3 |
| N-Glycan biosynthesis | hsa00510 | 1 | 50 | 0.672802 | 0.952836 | MGAT5B |
| Endocrine and other factor-regulated calcium reabsorption | hsa04961 | 1 | 50 | 0.672802 | 0.952836 | PRKCG |
| Autoimmune thyroid disease | hsa05320 | 1 | 53 | 0.694086 | 0.952836 | GZMB |
| cAMP signaling pathway | hsa04024 | 4 | 214 | 0.699158 | 0.952836 | GABBR1\|GIPR\|GLI1\|PDE4B |
| Regulation of actin cytoskeleton | hsa04810 | 4 | 214 | 0.699158 | 0.952836 | ITGAX\|ARHGEF4\|ITGB3\|FGF20 |
| Hepatitis B | hsa05161 | 3 | 163 | 0.701645 | 0.952836 | STAT5A\|MAP2K6\|PRKCG |
| Pathogenic Escherichia coli infection | hsa05130 | 1 | 55 | 0.707504 | 0.952836 | TUBB4A |
| Legionellosis | hsa05134 | 1 | 55 | 0.707504 | 0.952836 | EEF1A2 |
| TNF signaling pathway | hsa04668 | 2 | 112 | 0.71147 | 0.952836 | TNFRSF1B\|MAP2K6 |
| Toxoplasmosis | hsa05145 | 2 | 113 | 0.716079 | 0.952836 | CCR5\|MAP2K6 |
| Fatty acid metabolism | hsa01212 | 1 | 57 | 0.720338 | 0.952836 | ELOVL3 |
| Serotonergic synapse | hsa04726 | 2 | 115 | 0.725114 | 0.952836 | PRKCG\|PLA2G4D |
| Endometrial cancer | hsa05213 | 1 | 58 | 0.726543 | 0.952836 | TCF7L2 |
| Carbon metabolism | hsa01200 | 2 | 117 | 0.733905 | 0.952836 | TKTL1\|FBP1 |
| Yersinia infection | hsa05135 | 2 | 121 | 0.750777 | 0.952836 | WIPF3\|MAP2K6 |
| Arachidonic acid metabolism | hsa00590 | 1 | 63 | 0.755574 | 0.952836 | PLA2G4D |
| Neuroactive ligand-receptor interaction | hsa04080 | 6 | 338 | 0.761501 | 0.952836 | C3AR1\|CHRNB4\|CHRNA1\|GABBR1\|GIPR\|TSPO |
| Cortisol synthesis and secretion | hsa04927 | 1 | 65 | 0.766309 | 0.952836 | NR4A1 |
| Mitophagy - animal | hsa04137 | 1 | 65 | 0.766309 | 0.952836 | ATG9B |
| Inflammatory bowel disease (IBD) | hsa05321 | 1 | 65 | 0.766309 | 0.952836 | IL12RB2 |
| Retinol metabolism | hsa00830 | 1 | 67 | 0.776576 | 0.952836 | SDR16C5 |
| Long-term potentiation | hsa04720 | 1 | 67 | 0.776576 | 0.952836 | PRKCG |
| Autophagy - animal | hsa04140 | 2 | 128 | 0.778102 | 0.952836 | ATG9B\|DAPK2 |
| Glycolysis / Gluconeogenesis | hsa00010 | 1 | 68 | 0.781539 | 0.952836 | FBP1 |
| Amphetamine addiction | hsa05031 | 1 | 68 | 0.781539 | 0.952836 | PRKCG |
| Kaposi sarcoma-associated herpesvirus infection | hsa05167 | 3 | 186 | 0.782499 | 0.952836 | CCR3\|CCR5\|MAP2K6 |
| Adipocytokine signaling pathway | hsa04920 | 1 | 69 | 0.786393 | 0.952836 | TNFRSF1B |
| Central carbon metabolism in cancer | hsa05230 | 1 | 69 | 0.786393 | 0.952836 | GLS2 |
| Prolactin signaling pathway | hsa04917 | 1 | 70 | 0.79114 | 0.952836 | STAT5A |
| RIG-I-like receptor signaling pathway | hsa04622 | 1 | 70 | 0.79114 | 0.952836 | TBKBP1 |
| Epithelial cell signaling in Helicobacter pylori infection | hsa05120 | 1 | 70 | 0.79114 | 0.952836 | PTPRZ1 |
| FoxO signaling pathway | hsa04068 | 2 | 132 | 0.792517 | 0.952836 | BCL6\|FOXO6 |
| Oxidative phosphorylation | hsa00190 | 2 | 133 | 0.79599 | 0.952836 | COX6B2\|ATP12A |
| Melanoma | hsa05218 | 1 | 72 | 0.800321 | 0.952836 | FGF20 |
| p53 signaling pathway | hsa04115 | 1 | 72 | 0.800321 | 0.952836 | ADGRB1 |
| Huntington disease | hsa05016 | 3 | 193 | 0.803235 | 0.952836 | DNAI1\|COX6B2\|HIP1 |
| Apoptosis | hsa04210 | 2 | 136 | 0.806105 | 0.952836 | SEPTIN4\|GZMB |
| Thyroid hormone synthesis | hsa04918 | 1 | 74 | 0.809101 | 0.952836 | PRKCG |
| Measles | hsa05162 | 2 | 138 | 0.8126 | 0.952836 | IL2RB\|STAT5A |
| Glioma | hsa05214 | 1 | 75 | 0.813346 | 0.952836 | PRKCG |
| Biosynthesis of amino acids | hsa01230 | 1 | 75 | 0.813346 | 0.952836 | TKTL1 |
| Chronic myeloid leukemia | hsa05220 | 1 | 76 | 0.817497 | 0.952836 | STAT5A |
| Antigen processing and presentation | hsa04612 | 1 | 77 | 0.821557 | 0.952836 | CD8A |
| Drug metabolism - other enzymes | hsa00983 | 1 | 79 | 0.829408 | 0.957009 | UPB1 |
| Metabolic pathways | hsa01100 | 27 | 1432 | 0.842506 | 0.967163 | INMT\|CKB\|UPB1\|FBP1\|GAL3ST1\|COX6B2\|AZIN2\|TKTL1\|OLAH\|MGAT5B\|NT5C1A\|LDHD\|PDE5A\|NOS1\|HMGCS2\|ENTPD2\|LARGE2\|ENPP3\|ATP12A\|SORD\|ENPP1\|NAT8L\|PLA2G4D\|ETNPPL\|ELOVL3\|PDE4B\|GLS2 |
| Colorectal cancer | hsa05210 | 1 | 86 | 0.854278 | 0.975698 | TCF7L2 |
| Hepatitis C | hsa05160 | 2 | 155 | 0.860426 | 0.976658 | CLDN4\|PPP2R2C |
| PD-L1 expression and PD-1 checkpoint pathway in cancer | hsa05235 | 1 | 89 | 0.8638 | 0.976658 | MAP2K6 |
| Necroptosis | hsa04217 | 2 | 162 | 0.876674 | 0.983041 | STAT5A\|PLA2G4D |
| cGMP-PKG signaling pathway | hsa04022 | 2 | 167 | 0.887198 | 0.983041 | KCNMB4\|PDE5A |
| Endocrine resistance | hsa01522 | 1 | 98 | 0.888811 | 0.983041 | DLL4 |
| Ras signaling pathway | hsa04014 | 3 | 232 | 0.890648 | 0.983041 | PRKCG\|FGF20\|PLA2G4D |
| Phosphatidylinositol signaling system | hsa04070 | 1 | 99 | 0.891291 | 0.983041 | PRKCG |
| Chagas disease (American trypanosomiasis) | hsa05142 | 1 | 103 | 0.900673 | 0.986062 | PPP2R2C |
| Toll-like receptor signaling pathway | hsa04620 | 1 | 104 | 0.90289 | 0.986062 | MAP2K6 |
| Glucagon signaling pathway | hsa04922 | 1 | 106 | 0.907177 | 0.986062 | FBP1 |
| HIF-1 signaling pathway | hsa04066 | 1 | 109 | 0.913258 | 0.987899 | PRKCG |
| Neurotrophin signaling pathway | hsa04722 | 1 | 119 | 0.930807 | 0.991497 | BEX3 |
| Lysosome | hsa04142 | 1 | 123 | 0.936794 | 0.991497 | ABCA2 |
| Epstein-Barr virus infection | hsa05169 | 2 | 201 | 0.939447 | 0.991497 | MAP2K6\|CD19 |
| Insulin signaling pathway | hsa04910 | 1 | 137 | 0.95397 | 0.991497 | FBP1 |
| Estrogen signaling pathway | hsa04915 | 1 | 138 | 0.955002 | 0.991497 | GABBR1 |
| Signaling pathways regulating pluripotency of stem cells | hsa04550 | 1 | 140 | 0.956997 | 0.991497 | FZD4 |
| Parkinson disease | hsa05012 | 1 | 142 | 0.958905 | 0.991497 | COX6B2 |
| Phospholipase D signaling pathway | hsa04072 | 1 | 148 | 0.964136 | 0.991497 | PLA2G4D |
| Retrograde endocannabinoid signaling | hsa04723 | 1 | 148 | 0.964136 | 0.991497 | PRKCG |
| Non-alcoholic fatty liver disease (NAFLD) | hsa04932 | 1 | 149 | 0.964941 | 0.991497 | COX6B2 |
| Cellular senescence | hsa04218 | 1 | 160 | 0.972695 | 0.991497 | MAP2K6 |
| RNA transport | hsa03013 | 1 | 165 | 0.975631 | 0.991497 | EEF1A2 |
| Protein processing in endoplasmic reticulum | hsa04141 | 1 | 165 | 0.975631 | 0.991497 | ERO1B |
| Tuberculosis | hsa05152 | 1 | 179 | 0.982284 | 0.991497 | ITGAX |
| Alcoholism | hsa05034 | 1 | 180 | 0.982684 | 0.991497 | HDAC11 |
| Thermogenesis | hsa04714 | 1 | 231 | 0.994608 | 0.999048 | COX6B2 |
| Herpes simplex virus 1 infection | hsa05168 | 1 | 492 | 0.999988 | 0.999988 | ITGB3 |
