# Supplementary material for: Bone marrow stromal cells enhance multiple myeloma cells proliferation through regulating LncRNA OVAAL/ENPP1 axis
Source: Open Life Sci. 2026 May 14;21(1):20251322. doi: 10.1515/biol-2025-1322 (PMC13170762; doi:10.1515/biol-2025-1322)
Supplement: Supplementary file 3 — Supplementary Material [file j_biol-2025-1322_suppl_003.docx]

| Supplementary 3 Pathway enrichment analysis of downregulated differentially expressed mRNAs between U266 and co-cultured system. | | | | | | |
| --- | --- | --- | --- | --- | --- | --- |
| Term | ID | Input number | Background number | P-Value | Corrected P-Value | Input |
| Mineral absorption | hsa04978 | 3 | 53 | 0.000125 | 0.006359 | MT1E\|MT2A\|HMOX1 |
| Melanogenesis | hsa04916 | 2 | 101 | 0.015417 | 0.357021 | WNT10A\|EDNRB |
| Pantothenate and CoA biosynthesis | hsa00770 | 1 | 19 | 0.03571 | 0.357021 | DPYS |
| Hepatocellular carcinoma | hsa05225 | 2 | 168 | 0.03979 | 0.357021 | WNT10A\|HMOX1 |
| beta-Alanine metabolism | hsa00410 | 1 | 33 | 0.061258 | 0.357021 | DPYS |
| Aldosterone-regulated sodium reabsorption | hsa04960 | 1 | 37 | 0.06844 | 0.357021 | SCNN1G |
| Ferroptosis | hsa04216 | 1 | 40 | 0.073793 | 0.357021 | HMOX1 |
| Pathways in cancer | hsa05200 | 3 | 530 | 0.075769 | 0.357021 | WNT10A\|EDNRB\|HMOX1 |
| Porphyrin and chlorophyll metabolism | hsa00860 | 1 | 42 | 0.077346 | 0.357021 | HMOX1 |
| Sphingolipid metabolism | hsa00600 | 1 | 47 | 0.086172 | 0.357021 | SGPP2 |
| Pyrimidine metabolism | hsa00240 | 1 | 57 | 0.103589 | 0.357021 | DPYS |
| Basal cell carcinoma | hsa05217 | 1 | 63 | 0.11389 | 0.357021 | WNT10A |
| Staphylococcus aureus infection | hsa05150 | 1 | 68 | 0.12239 | 0.357021 | MASP2 |
| Human papillomavirus infection | hsa05165 | 2 | 330 | 0.129053 | 0.357021 | ITGA8\|WNT10A |
| Arrhythmogenic right ventricular cardiomyopathy (ARVC) | hsa05412 | 1 | 77 | 0.137497 | 0.357021 | ITGA8 |
| Synaptic vesicle cycle | hsa04721 | 1 | 78 | 0.139161 | 0.357021 | SLC6A9 |
| Drug metabolism - other enzymes | hsa00983 | 1 | 79 | 0.140821 | 0.357021 | DPYS |
| Complement and coagulation cascades | hsa04610 | 1 | 79 | 0.140821 | 0.357021 | MASP2 |
| PI3K-Akt signaling pathway | hsa04151 | 2 | 354 | 0.144726 | 0.357021 | ITGA8\|EFNA2 |
| Taste transduction | hsa04742 | 1 | 83 | 0.147434 | 0.357021 | SCNN1G |
| ECM-receptor interaction | hsa04512 | 1 | 86 | 0.152362 | 0.357021 | ITGA8 |
| Hypertrophic cardiomyopathy (HCM) | hsa05410 | 1 | 90 | 0.158891 | 0.357021 | ITGA8 |
| Fc gamma R-mediated phagocytosis | hsa04666 | 1 | 94 | 0.165374 | 0.357021 | BIN1 |
| Dilated cardiomyopathy (DCM) | hsa05414 | 1 | 96 | 0.168598 | 0.357021 | ITGA8 |
| Inflammatory mediator regulation of TRP channels | hsa04750 | 1 | 100 | 0.17501 | 0.357021 | TRPV3 |
| HIF-1 signaling pathway | hsa04066 | 1 | 109 | 0.18927 | 0.366394 | HMOX1 |
| Sphingolipid signaling pathway | hsa04071 | 1 | 119 | 0.204845 | 0.366394 | SGPP2 |
| Relaxin signaling pathway | hsa04926 | 1 | 130 | 0.221654 | 0.366394 | EDNRB |
| Fluid shear stress and atherosclerosis | hsa05418 | 1 | 139 | 0.23516 | 0.366394 | HMOX1 |
| Signaling pathways regulating pluripotency of stem cells | hsa04550 | 1 | 140 | 0.236647 | 0.366394 | WNT10A |
| Cell adhesion molecules (CAMs) | hsa04514 | 1 | 146 | 0.245513 | 0.366394 | ITGA8 |
| Breast cancer | hsa05224 | 1 | 147 | 0.246981 | 0.366394 | WNT10A |
| Gastric cancer | hsa05226 | 1 | 149 | 0.249909 | 0.366394 | WNT10A |
| mTOR signaling pathway | hsa04150 | 1 | 153 | 0.255734 | 0.366394 | WNT10A |
| Hippo signaling pathway | hsa04390 | 1 | 154 | 0.257184 | 0.366394 | WNT10A |
| Cushing syndrome | hsa04934 | 1 | 155 | 0.258631 | 0.366394 | WNT10A |
| Wnt signaling pathway | hsa04310 | 1 | 160 | 0.265827 | 0.36641 | WNT10A |
| cGMP-PKG signaling pathway | hsa04022 | 1 | 167 | 0.275792 | 0.370142 | EDNRB |
| Axon guidance | hsa04360 | 1 | 181 | 0.295344 | 0.384764 | EFNA2 |
| Calcium signaling pathway | hsa04020 | 1 | 193 | 0.311709 | 0.384764 | EDNRB |
| Focal adhesion | hsa04510 | 1 | 199 | 0.319758 | 0.384764 | ITGA8 |
| Proteoglycans in cancer | hsa05205 | 1 | 203 | 0.325075 | 0.384764 | WNT10A |
| Rap1 signaling pathway | hsa04015 | 1 | 210 | 0.334287 | 0.384764 | EFNA2 |
| Regulation of actin cytoskeleton | hsa04810 | 1 | 214 | 0.339498 | 0.384764 | ITGA8 |
| cAMP signaling pathway | hsa04024 | 1 | 214 | 0.339498 | 0.384764 | CNGB3 |
| Ras signaling pathway | hsa04014 | 1 | 232 | 0.36248 | 0.40188 | EFNA2 |
| Endocytosis | hsa04144 | 1 | 244 | 0.377384 | 0.409502 | BIN1 |
| MAPK signaling pathway | hsa04010 | 1 | 295 | 0.437173 | 0.459654 | EFNA2 |
| MicroRNAs in cancer | hsa05206 | 1 | 299 | 0.441628 | 0.459654 | HMOX1 |
| Neuroactive ligand-receptor interaction | hsa04080 | 1 | 338 | 0.483373 | 0.493041 | EDNRB |
| Metabolic pathways | hsa01100 | 2 | 1432 | 0.78812 | 0.78812 | DPYS\|HMOX1 |
